# Supplementary material for: An altruistic rhizo-microbiome strategy in crop-rotation systems for sustainable management of soil-borne diseases
Source: Plant Commun. 2025 Sep 3;6(10):101502. doi: 10.1016/j.xplc.2025.101502 (PMC12546766; doi:10.1016/j.xplc.2025.101502)
Supplement: Document S1. Figures S1–S34, Tables S1–S9, and supplemental methods [file mmc1.pdf]

Plant Communications, Volume *6*

Supplemental information

An altruistic rhizo-microbiome strategy in crop-rotation systems for sustainable management of soil-borne diseases

Jiaqing Wu, Yixiang Liu, Huanjie Yu, Fuyuan Fan, Xiahong He, Youyong Zhu, Yang Dong, Min Yang, and Shusheng Zhu

Supplemental Methods

Meta-analysis

Literatures were searched in Google Scholar and CNKI using "garlic" and "crop rotation" as the primary keywords. Papers were selected according to the following criteria (Zhang et al., 2022): (a) the experimental type must be a field or pot experiment; (b) the experimental research parameters must include crop yield; (c) the mean, standard deviation (or standard error) and sample size must be available or calculable. For each selected study, the original data were collected directly from the tables and texts. If the data appeared in the form of graphics, the required data were obtained online using WebPlotDigitizer (https://apps.automeris.io/wpd/index.zh_CN.html). If there is no standard error (SE), SE is calculated as SE=1/4×mean (Dynarski et al., 2018). If the data provided in the literature is the standard error (SE), the standard deviation (SD) can be converted using the following equation:

$$SD=SE\sqrt{n}, \quad ①$$

where n = number of replicates.

According to the above screening criteria, a total of 21 articles were finally obtained (Supplemental Table 2), and 70 experimental data were used for meta-analysis. The garlic planting group was used as the treatment group, and any treatment without garlic, including fallow or wheat planting (winter wheat planting time is similar to garlic) or continuous planting as the control group, the average yield, standard deviation and number of replicates of the subsequent crops in each study were extracted to calculate the effect size lnR (Zheng et al., 2019):

$$\ln R=\ln(Y_e/Y_c), \quad ②$$

Y_e is the yield of the experimental group; Y_c is the yield of the control group. The yield units of field trials are uniformly converted to kg hm^{-2}, and the yield units of pot trials are uniformly converted to g $plant^{-1}$.

The mean and variance of lnR were analyzed by MetaWin 3.0.14 software (http://www.metawinsoft.com). The random effect size and variance were calculated, and the global effect size was further calculated using MetaWin 3.0.14 software with a confidence interval (CI) of 95%. The significance level was reached when the 95% CI crossed the zero line.

Due to differences in geographical factors, climatic conditions, field management measures, and

soil fertility among the studies, there was a large variability in the research results, so a random effects model was selected for analysis.

This study used the heterogeneity between groups ($Q_{Between}$) to test the difference in the effect size of the same indicator between different subgroups and to analyze the heterogeneity between groups. If $p < 0.05$, the heterogeneity between groups is considered significant; otherwise, it is considered insignificant (Nie et al., 2023).

The data were tested for publication bias using Egger's method. If the Egger's regression result $p > 0.05$, the data were considered reliable (Nie et al., 2023).

Plant materials

Garlic (*Allium sativum*, purple-skinned variety) used in this study was sourced from a local market in Yunnan. The pepper (*Capsicum annuum*) used in the indoor and field experiments was six-color pickled pepper F1 (Guangxi Hengxian Zilong Seed Industry Co., Ltd.), the tobacco was *Nicotiana benthamiana*, and the potato was purple potato.

Collection of garlic-conditioned soil

Prior to sowing, the garlic was subjected to surface disinfection using a 1.5% sodium hypochlorite solution. To facilitate the collection of conditioned soil for subsequent experiments, a root bag method was employed. The procedure involved placing a nylon root bag (upper diameter of 11 cm and height 10 cm) inside each plastic flower pot (inner diameter of 13.5 cm and height of 14.5 cm). Each bag was filled with 600 g of soil, while an additional 1.2 kg of soil was packed into the gap between the bag and the pot. This setup created two distinct compartments: the interior of the bag, which constituted the rhizosphere, and the area between the bag and the pot, representing the root circumference. The root bag allowed the penetration of small molecular compounds while preventing root intrusion (Supplemental Figure 34).

The planting trial was conducted over two seasons. In the first season, garlic-conditioned soil was collected to evaluate its effects on the growth of three Solanaceous crops (pepper, tobacco, potato) and the incidence of *Phytophthora* blight in peppers. The second season focused on further assessment the impacts of garlic-conditioned soil with varying planting density on the blight as well as the composition of soil microbial communities (Ding et al., 2018; Guo et al., 2019).

One to five cloves of garlic were sown in each bag, while soil treated similarly but without any planted crops served as a control. After 30 days of regular watering, the garlic rhizosphere soil and control soil were collected separately. Each replicate consisted of four pots, which were subsequently combined into a single composite sample for analysis.

Inoculation of *Phytophthora capsici* on the stem of pepper

Method for inoculating *P. capsici* by scratching the base of pepper seedling stems: a sterilized blade was used to create a wound on the epidermis of the stem base of the pepper seedling, approximately 7 mm in length and located close to the soil. A 7 mm diameter cake was then applied to the incision surface, followed by a layer of absorbent cotton. 1 mL of sterile water was injected into the absorbent cotton to moisten it (Yu et al., 2024).

Pepper metabolite extraction and analysis

The changes in metabolites of pepper were measured following previous report with some modifications (Liu et al., 2023). The leaves of pepper treated with garlic soil and garlic soil-filtered (CK) were ground in liquid nitrogen, respectively, which were used for metabolite extraction. 60 mg samples were weighed and ultrasonically extracted at 37°C for 30 min with 1 mL of extraction solution prepared by methanol, chloroform and sterilized deionized water in a volume ratio of 5:2:2. Then the homogenates were centrifuged for 3 min (1600 g, 4°C). After that, the supernatant was transferred and dried using a SpeedVac (Christ, Germany) at 25°C. A total of 80 μL of 20 $mg \cdot mL^{-1}$ methoxyamine hydrochloride solution dissolved in pyridine and 40 μL of N-methyl-N-(trimethylsilyl)-trifluoroacetamide were added to the dried samples in two steps, and reacted for 90 min, 30°C and 30 min, 37°C respectively for derivatization. Finally, each sample was centrifuged for 3 min (1600 g, 4°C) and stored at 4°C for metabolite analysis. Based on a previously reported method, gas chromatography−mass spectrometry (GC−MS; QP2010 Ultra, Shimadzu, Kyoto, Japan) was used to analyse the metabolites of pepper. The model of chromatographic column was SH-Rxi-5Sil MS and 30.0 m × 0.25 mm × 0.25 μm. The offline data was first converted to abf format using Analysis Base File Converter, and then processed by MSDIAL for peak search, peak alignment, and identification of metabolite ion peaks. Based on a fold change (FC) >1.5 or <0.67 and $p < 0.05$, the differentially accumulated metabolites (DAMs) were screened (Liu et al., 2023).

Isolation, identification and functional verification of garlic-conditioned **soil microorganisms**

5 grams of garlic-conditioned soil samples from different densities were placed into 50 mL sterile centrifuge tubes. Then, 45 mL of sterile water was added, and the mixture was shaken at 120 rpm for 30 minutes. The solution was allowed to stand for 15 minutes to create a soil suspension, which was subsequently diluted 100-fold with sterile water. A volume of 50 μL of this suspension was spread evenly onto the surface of various Bengal red solid culture media and incubated at 28°C in a constant temperature incubator. Single colonies were selected from the corresponding plates for purification. Following the method of Visagie et al., 2014, single colonies were observed on both sides of PDA medium for colony shape, color, texture, and colony margins. Conidia and sporangia morphology were examined under a light microscope. PCR amplification was conducted using ITS and β-tubulin (*TUB2*) primers. The amplified products were purified and subjected to Sanger sequencing. The sequencing results were compared against the GenBank database via BLAST analysis. A phylogenetic tree was constructed with MEGA11 software using the neighbor-joining method. The molecular and morphological identification results were integrated to determine the pathogen's species.

An antagonistic test was conducted between isolated fungi and *P. capsici*. Different fungi were activated, and a 7 mm diameter *P. capsici* plug was inoculated at the center of the PDA medium. The plugs of the test strains were then inoculated 22 mm away from the *P. capsici* plugs. As a control, *P. capsici* was inoculated only in the center of the plate. Each strain was replicated four times. Inhibition rate (%) = (average diameter of control treatment − average diameter of experimental treatment)/(average diameter of control treatment) × 100.

Induced resistance test for pepper disease. The isolated fungi plug was placed in liquid PDA medium and cultured at 26°C for 5-7 days until the hyphae covered the medium. It was then broken down using a juicer and diluted five times with water for subsequent use. The base of the pepper stem was inoculated with the blight; the fungal fermentation liquid was applied once prior to inoculation and again following inoculation. The length of the lesions was measured 7 days post-inoculation. Three pepper plants were inoculated per pot, with each pot serving as a replicate, and four replicates were included for each treatment. Additionally, 24 hours after fungal treatment, the aboveground parts of pepper seedlings were harvested to detect salicylic acid pathway resistance genes *(PAL* and *PR1c*; primer sequences provided in Supplemental Table 9), as described by Diao

et al. (2019), Zhao et al. (2022), and Zhang et al. (2020). Salicylic acid of pepper seedlings were quantified according to Luo et al. (2025).

Three *Penicillium* strains (*P. allii* YNAU-Q-6, *P. ochrochloron* YNAU-P-4, and *P. brevicompactum* YNAU-Q-9) were evaluated using 5-mm mycelial plugs from 7-day-old PDA cultures. Garlic seedlings and uniform cloves (30-35 mm) were surface-sterilized with 1% NaClO (3 min), then triple-rinsed. Standardized 5-mm wounds were inoculated with 5-mm fungal plugs, with PDA-only and non-wounded controls. Incubation at 28°C for 3 days allowed daily disease assessment. Rot severity was observed by lesion diameter. In addition, the same method was used to detect the effect of *Penicillium* on the disease resistance genes of garlic seedlings (*PAL* and *PR1c*; primer sequences provided in Supplemental Table 9), as described by Tuan et al. (2010) and Anisimova et al. (2021).

GC-MS conditions

GC conditions: HP-5MS chromatography column (30.0m × 0.25mm × 0.25 μm). Starting column temperature: 40 °C, after heating up to 80 °C at 3.0/min, raise the temperature to 270 °C at 5.0 °C/min and maintain it for 10.0 minutes. The carrier gas is helium, with a sample inlet temperature of 250.0 °C and a column box temperature of 40.0 °C. The injection method is direct injection without splitting.

MS conditions: EI ionization source, ion source temperature 230 °C, interface temperature 250 °C, scanning range m/z 35-500, acquisition method Scan, scanning interval 0.30s. The identification of volatile components in roots was carried out by searching the retention time in the NIST14/ NIST14s spectrogram library. Garlic sulfide compounds were determined by comparing the characteristic fragment ions with the characteristic fragment ions of sulfide compound standards (Wu et al., 2024).

Effect of DADS and H_2O_2 treatment for the growth of *P. allii*, *C. destructans* and *P. capsici*

10 blocks of *P. allii, C. destructans and P. capsici* were added to 100 mL of potato glucose liquid culture medium and placed on a shaking table for pre-incubating at 140 rpm and 28°C for 120 hours. The culture medium was supplemented DADS with final concentrations of0, 13.7, 27.3, 54.7, 109.4, 683.7 μM or H_2O_2 with final concentrations of 85.9, 1717.1, 4292.9μM. After being subjected to continuous shaking cultivation at 28 °C for 72 hours. Collecting the mycelial balls and dry them in an oven for weight (Wu et al., 2024).

Detection of Thiamine Utilization by Soil Microorganisms

Five grams of soil from different treatments (CK, Z3, D3, and H3) were weighed, and 45 mL of ddH_2O was added. The mixture was shaken at 150 rpm for 30 min to prepare a soil suspension. The suspension was filtered through filter paper, and the filtrate was centrifuged at 3000 rpm and 4°C for 10 min, with the supernatant discarded. The pellet was resuspended in 20 mL of 296.5 μM thiamine solution and shaken at 150 rpm for 24 h. The mixture was reacted with the test solution for 10 min before and after the 24 h shaking period. The OD_{704} values were measured before and after fermentation using a microplate reader (Li et al., 2022). Thiamine utilization efficiency was calculated using the following formula:

$$\frac{\text{Initial absorbance value} - \text{Absorbance value after 24 hours}}{\text{Initial absorbance value} - \text{Blank absorbance value}} \times 100\%$$

Supplemental Table 1 Fungi and Bacteria analysis of similarities (ANOSIM) in garlic treatment

		Garlic VS CK		Z1 VS CK		Z2 VS CK		Z3 VS CK		Z5 VS CK	
	ANOSIM	R	*p*-value	R	*p*-value	R	*p*-value	R	*p*-value	R	*p*-value
Genus	Fungi	0.3056	0.043	0.1042	0.065	0.2604	0.040	0.3646	0.040	0.8854	0.040
	Bacteria	0.0881	0.249	-0.1563	0.86	-0.125	0.767	-0.0625	0.625	0.03125	0.346
ASV	Fungi	0.3388	0.011	0.2360	0.023	0.1680	0.028	0.4960	0.015	0.9560	0.015
	Bacteria	0.1550	0.164	-0.2292	0.949	-0.0625	0.617	-0.0313	0.535	0.3542	0.115

Note: Z1, Z2, Z3, and Z5 represent 1, 2, 3, and 5 garlic plants per pot, respectively. CK represents control group.

Supplemental Table 2 Fungi analysis of similarities (ANOSIM) in DADS treatment

	DADS VS CK		D1 VS CK		D2 VS CK		D3 VS CK		D4 VS CK	
ANOSIM	R	*p*-value	R	*p*-value	R	*p*-value	R	*p*-value	R	*p*-value
Genus	0.4896	0.021	0.0625	0.240	0.0313	0.290	0.0521	0.205	-0.0417	0.623
ASV	0.4063	0.023	0.0360	0.325	0.1520	0.064	0.1240	0.080	-0.0440	0.617

Note: D1, D2, D3, and D4 represent soil DADS concentrations of 13.7, 27.3, 54.7, and 109.4 μmol kg^{-1}, respectively. CK represents control group.

Supplemental Table 3 Fungi analysis of similarities (ANOSIM) in H_2O_2 treatment

	H_2O_2 VS CK		H1 VS CK		H2 VS CK		H3 VS CK		H4 VS CK	
ANOSIM	R	*p*-value	R	*p*-value	R	*p*-value	R	*p*-value	R	*p*-value
Genus	0.6223	0.005	0.1354	0.092	0.1771	0.040	0.0833	0.178	0.1667	0.069
ASV	0.6040	0.002	0.1560	0.057	0.2160	0.027	-0.0160	0.522	0.2040	0.015

Note: H1, H2, H3, and H4 represent soil H_2O_2 concentrations of 85.9, 171.7, 343.4, and 686.9 μmol kg^{-1}, respectively. CK represents control group.

Supplemental Table 4 Volatile components in root exudates of garlic seedlings

Compound Name	Relative peak area%
Diallyl disulfide	44.39418
Dibutyl phthalate	17.38317
1,2-Benzenedicarboxylic acid, bis(2-methylpropyl) ester	8.397252
Oxalic acid, cyclohexylmethyl octadecyl ester	5.223487
Heptacosyl heptafluorobutyrate	4.849501
Sulfurous acid, di(cyclohexylmethyl) ester	3.538899
Bis(2-ethylhexyl) phthalate	3.292318
Sulfurous acid, cyclohexylmethyl octadecyl ester	2.401355
1,2-Benzenedicarboxylic acid, bis(2-methylpropyl) ester	2.222368
Octacosyl pentafluoropropionate	2.130469
Butanoic acid, ethyl ester	1.981014
Phenol, 2,2'-methylenebis[6-(1,1-dimethylethyl)-4-methyl-	1.108266
Propanoic acid, 2-methyl-, ethyl ester	0.699461
5,5,8a-Trimethyldecalin-1-one	0.564236
Sulfurous acid, cyclohexylmethyl pentadecyl ester	0.485706
Heptacosyl heptafluorobutyrate	0.443981
13-Tetradecen-1-ol acetate	0.410959
Heneicosanoic acid, methyl ester	0.240738
erythro-7,8-Bromochlorodisparlure	0.232643

Supplemental Table 5 Volatile compounds in field-collected garlic rhizosphere soil

Compound Name	Relative peak area%
Diallyl disulphide	39.67674
1-Allyl-2-isopropyldisulfane	23.56737
.alpha.-Pinene	9.053534
Disulfide, dipropyl	8.152234
Naphthalene, decahydro-, trans-	2.664853
o-Xylene	2.377539
3-Undecene, 6-methyl-, (E)-	1.990223
3-Hydroxy-3-methylvaleric acid	1.535884
Ethanone, 1-cyclohexyl-	1.349859
Decyl heptyl ether	1.211857
Caryophyllene oxide	1.15235
.beta.-Longipinene	0.957287
Caryophyllene	0.931622
Benzene, 1,4-diethyl-	0.857209
Phenol, 2,2'-methylenebis[6-(1,1-dimethylethyl)-4-methyl-	0.834565
Aciphyllene	0.834091
2H-2,4a-Methanonaphthalene, 1,3,4,5,6,7-hexahydro-1,1,5,5-tetramethyl-, (2S)-	0.763848
Sulfurous acid, 2-ethylhexyl nonyl ester	0.574802
2H,8H-Benzo[1,2-b:5,4-b']dipyran-10-propanoic acid, 5-methoxy-2,2,8,8-tetramethyl-, methyl ester	0.558611
trans-Decalin, 2-methyl-	0.533582
1,2,4-Methenoazulene, decahydro-1,5,5,8a-tetramethyl-, [1S-(1.alpha.,2.alpha.,3a.beta.,4.alpha.,8a.beta.,9R*)]-	0.421932

Supplemental Table 6 Volatile compounds in pot-grown garlic-conditioned soil (Z3 treatment)

Compound Name	Relative peak area%
Diallyl disulfide	56.89708
Dibutyl phthalate	13.42136
2-Ethylhexyl salicylate	11.32036
1,2-Benzenedicarboxylic acid, bis(2-methylpropyl) ester	7.717764
Homosalate	5.15351
2,2,4-Trimethyl-1,3-pentanediol diisobutyrate	1.797997
Sulfurous acid, 2-pentyl undecyl ester	1.739104
Eicosane	0.884293
Heptadecane	0.860879
Cedrol	0.207662

Supplemental Table 7 Meta-analysis data extraction literature

Number	Literature
1	**Yang, J.B., Sun, L.H., Li, Z.M., Guo, W.C., and Guo, D.Q.** (2021). Effects of different varieties and different stubble on *Pinellia ternata* yield. Bull. Agric. Sci. Technol. **0**:181-183. http://tongxun.aiijournal.com/CN/Y2021/V0/I1/181.
2	**Yang, R.P., Mo, Y.L., Liu, C.M., Wang, Y.Q., Ma, J.X., Zhang, Y., Li, H., and Zhang, X.** (2016). The effects of cattle manure and garlic rotation on soil under continuous cropping of watermelon (*Citrullus lanatus* L.). PLoS One **11**:e0156515. https://doi.org/10.1371/journal.pone.0156515.
3	**Dhillon, N.K., Kaur, S., Sidhu, H.S., and Anupam** (2019). Management of root knot nematode opting garlic crop in vegetable based cropping systems. Indian J. Hortic. **76**: 472-478. https://doi.org/10.5958/0974-0112.2019.00075.6.
4	**Ding, H.Y., Ali, A., and Cheng, Z.H.** (2018). Dynamics of a soil fungal community in a three-year green garlic/cucumber crop rotation system in Northwest China. Sustainability **10**:1391. https://doi:10.3390/su10051391.
5	**Li, Z.S., Xiang, Y.H., Jiang, J.Q., Tang, L.W., Teng, Z., and Yan, Q.F.** (2019). A study on the effects of different previous crops on yield and quality of flue-cured tobacco in Panzhihua Area. J. Xichang Univ. (Nat. Sci. Ed.) **33**:10-12. https://doi.org/10.16104/j.issn.1673-1891.2019.01.003.
6	**Liu, W.X., F. X.Y, Zhang F.Y., He, Q,L., Chen, L., Li K., and Wu. J.H.** (2021). Effects of different preceding crops and seed coating agent dosage on peanut diseases, pests yield. Crops **37**:199-204. https://doi.org/10.16035/j.issn.1001-7283.2021.06.032.
7	**Zhang, G.W., Wang, X.Q., Yang, C.Q., Shu, H.M., and Liu, R.X.** (2021). Effects of rotational pattern and fertilization application on soybean yield under straws returning of preceding crop. Chin. J. Eco-Agric. **29**:1493-1501. https://doi.org/10.13930/j.cnki.cjea.210084.
8	**Zhou, Q., Zhang, P., Wang, Z.Q., Wang, L.X., Wang, S.B., Yang, W.T., Yang, B.J., and Huang, G.Q.** (2023). Winter crop rotation intensification to increase rice yield, soil carbon, and microbial diversity. Heliyon **9**:e12903. https://doi.org/10.1016/j.heliyon.2023.e12903.
9	**Wang, T., Chen, H., Zhou, W., Chen, Y., Fu, Y., Yang, Z.P., Liu, Q., Yue, X.P., Deng, F., Nkrumah, M., et al.** (2022). Garlic–rice system increases net economic benefits and reduces greenhouse gas emission intensity. Agric. Ecosyst. Environ.326:107778. https://doi.org/10.1016/j.agee.2021.107778.

Number	Literature
10	**Huang, G.R., Zhang, Z.** (2007) The effect of tobacco rotation with different crops on the growth, development, and yield of tobacco. **6**:30-31. https://doi.org/10./j.cnki.52—1065/s.2007.06.014.
11	**Jia, W.Y., Liang, Y.L. Bai, C.H., Zhu, Y.L., Peng, Q., Lin, X.J., and Chen, C.** (2011). Effects of different proceding crops on growth physiology and quality of hot pepper. Agr. Res. Arid Areas.**29**:151-156.
12	**Chuan, YC., Zhang L.M., Jiao Y.G., Luo, L.F., Fang, Y.T., Liao, J.J., JI, S.G., Zhu, S.S., and Yang. M.** (2016). Control effects of tobacco and garlic rotation on tobacco black shank and a preliminary study on the inhibition mechanism. Acta Tab. Sin. **22**:55-62. https://doi.org/10.16472/j.chinatobacco.2016.200
13	**Tang, B., Zhang, X.Z., and Yang, X.B.** (2015). Effects of tobacco garlic crop rotation and intercropping on tobacco yield and rhizosphere soil phosphorus fractions. Chin. J. Appl. Ecol. **26**:1977-1984. https://pubmed.ncbi.nlm.nih.gov/26710622/.
14	**Miao, Q.S, Wang, D.S., Wei, Y.G., Huang, S.H, and Zhang, Y.Y.** (2021). Effects of intercropping and rotation on the growth of continuous crop eggplant and the incidence of *Verticillium* wilt. Chin. J. Veg. Sci. **34**:33-40. https://doi.org/10.16861/j.cnki.zggc.2021.0319.
15	**Jia, W.Y., Liang, Y.L. Bai, C.H., Zhu, Y.L., Peng, Q., Lin, X.J., Peng, Q., and Chen, C.** (2010). Effect of fore crops on growth yield and quality of hot pepper. J. Northwest A&F Univ. **38**:119-130 (Nat. Sci. Ed.). https://doi.org/10.13207/j.cnki.jnwafu.2010.05.024.
16	**Shi, G.H., Liang, Y.L., Yao, X.W., Zeng, R., and Mu, L.** (2013). Effects of Actinomycetes on yields and qualities of tomato and pepper under different fore Crops. Bull. Soil Water Conserv. 33:275-279. https://doi.org/10.13961/j.cnki.stbctb.2013.01.060.
17	**Hao, W.L., Liang, Y.L, Zhu, Y.L., Wu, X., Lin, X.J., and Luo, A.R.** (2011). Production efficiency and soil nutrient characteristics in food--vegetable rotation systems. Bull. Soil Water Conserv. **31**:46-51. https://doi.org/10.13961/j.cnki.stbctb.2011.02.031.
18	**Lee, H.U., Kim, C.H., and Nam, K.W.** (1991) Suppression of Phytophthora blight incidence of red pepper by cropping system. Plant Pathol. J. **7**: 147-152.
19	**Chen, Y.L., Lin, Z.Q., Tuo, Y.Y., Ding, Y.R., Li, H.L., and Wang, Y.** (2022). Effects

Number	Literature
	of tobacco garlic crop rotation on fungal community structure of susceptible tobacco soil. Southwest China J. Agri. Sci. **35**:972-980. https://doi.org/10.16213/j.cnki.scjas.2022.4.030.
20	**Yang, X.B., Li, T.X., Zhang, X.Z., Chen, D.R., Liang, Y.J., and Zhang, C.H**. (2016). Effects of tobacco garlic crop rotation and tobacco garlic crop intercropping on soil microbial groups in tobacco fields. Soils. **48**:698-704. https://doi.org/10.13758/j.cnki.tr.2016.04.012.
21	**Zhang, T., Chen, A.Q., Liu, J., Liu, H.B., Lei, B.K., Zhai, L., Zhang, D., and Wang, H.Y.** (2017). Cropping systems affect paddy soil organic carbon and total nitrogen stocks (in rice-garlic and rice-fava systems) in temperate region of southern China. Sci. Total Environ. **609**:1640-1649. https://doi.org/10.1016/j.scitotenv.2017.06.226.

Supplemental Table 8 The primers of three microorganisms for RT-primer

Species	Gene name	Sequence 5' to 3'
Penicillium allii	*GST*	TTTGAGCTTCGTTGCTTGGC
		CAGTCGGTCTCTCCTGCATC
	CYP628	TCGTTCGCCTCAGATCATCG
		TGCACATCAGTTTCCCTCGT
	FSP1	ATCAACCCATCGCCAACCTT
		ACTGGCTGAACTCCCTTTCG
	18S (reference gene)	GATGAAGAACGCAGCGAAAT
		TTGAAATGACGCTCGAACAG
Cylindrocarpon destructans	*ECHS1*	CAGCCTGGATGCTGTCTGTA
		CTTTTAGGGTCCCCCATCTC
	GST	AACCCAACTCGCCAAGTCAA
		CAGCCACTTCTCCAGGTGAG
	CHS1	TCATGTTCGATCCCTGGCAC
		TGCTGACCGAAGGGAGTTTC
	CYP53A1	TGTCTTACGGCCAAGACCAC
		ACAATTCTCAGCATCCCGCA
	18S (reference gene)	GATGAAGAACGCAGCGAAAT
		TTGAAATGACGCTCGAACAG
Phytophthora capsici	*SOD2*	TTCTCGCATCGCCTCTTCCG
		TTAGGAGCCAGGTTCGTCCA
	bglX	GTTCTCAATGGTGGCTTCGC
		ATCGGGTGTCCTCCGTTTTC
	ABC1	CAACACTCGCACACACAACA
		ATTTCACGCTTGGAGGGTCC
	CDC6	ATTGAACGCACAACAACCCG
		CGCGTGCTTGATAATGCGAA
	Tublin B (reference gene)	CCAGCTTCAGCCTTCACTTC
		CATCCCAATCCTGATCCTGT

Supplemental Table 9 The primers of pepper and garlic for RT-qPCR

Species	Gene name	Sequence 5' to 3'
Pepper	*PAL*	CAACAGCAACATCACCCCATGTTTGC
		GCTGCAACTCGAAAAATCCACCAC
	PR1c	AAATGCAGCACGTTCAGCA
		CGTTCCCACTTCCCCAAA
	Actin-1 (reference gene)	GTCCTCTTCCAACCATCCAT
		TACTTTCTCTCTGGTGGTGC
Garlic	*PAL*	GAAGTCGTTCACCAGCTCGGAGAAC
		GACGTGTCAAGGAAAAAGCCGTG
	PR1c	GGCGGTCCTTATGGTGAAA
		GCCAGGGTCACATGTGTTA
	GAPDH (reference gene)	AGGCTGGTGCTGATTACG
		AGGTCTGAAGTGTATGAAGTATGG

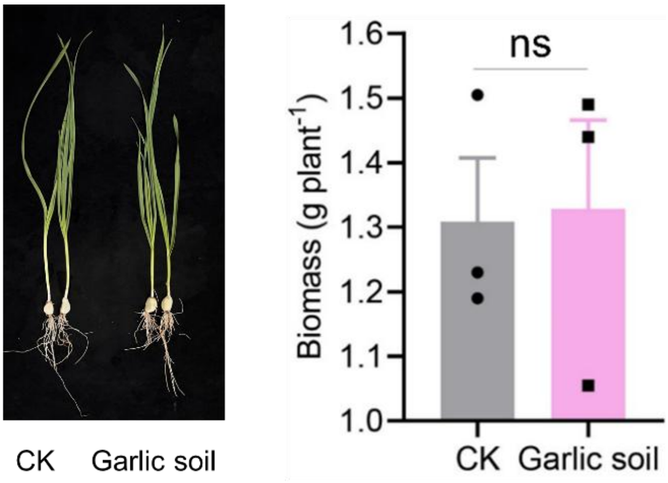

Supplemental Figure 1 Effect of garlic-conditioned soil on the growth of garlic.

CK soil represents the blank treatment without garlic planting, and Garlic soil represents the garlic-conditioned soil treatment. Data are expressed as mean ± standard error ($n = 3$). Data are expressed as mean ± SE. ns represents no significant difference. (Independent sample t test, $p < 0.05$)

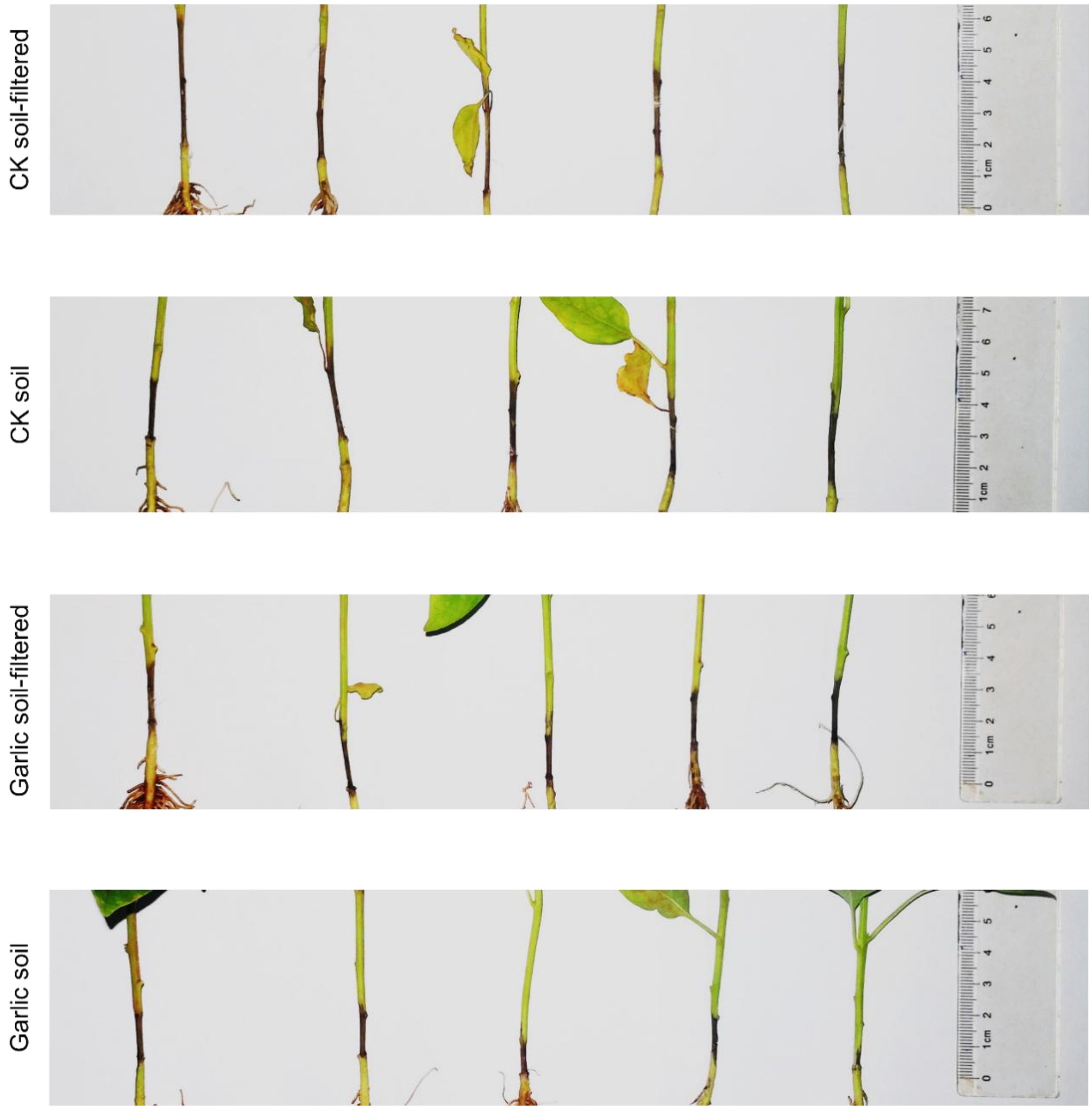

Supplemental Figure 2 Effects of garlic soil suspension and control soil suspension on the expansion of pepper blight lesions.

CK soil represents the blank treatment without garlic planting, and garlic soil represents the garlic-conditioned soil treatment. The soil suspensions were filtered through a 0.22 μm filter to remove microorganisms.

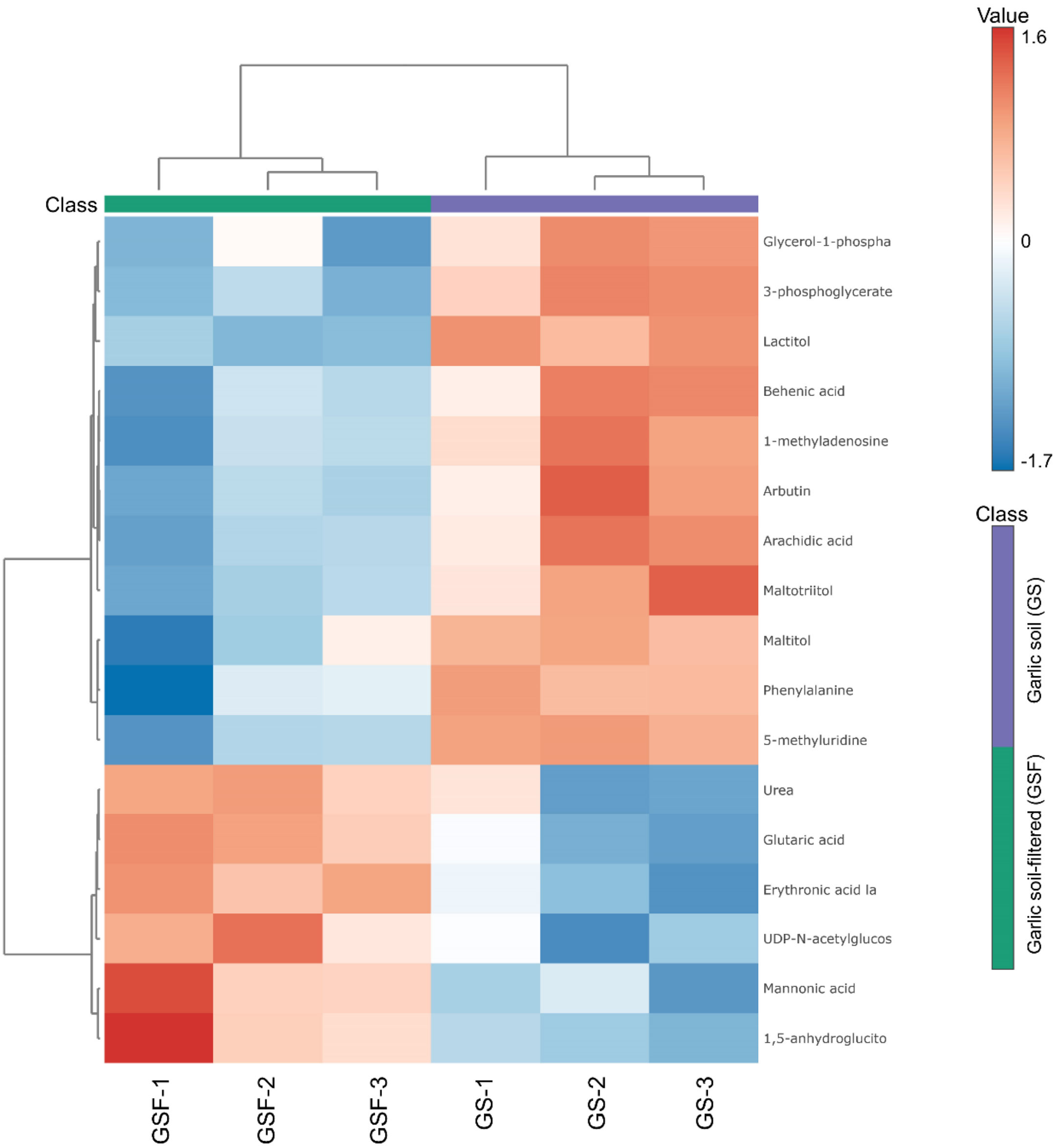

Supplemental Figure 3 Heat map of differential metabolites in the shoots of pepper after exposured to filtered and unfiltered garlic soil suspension.

GSF represents for filtered garlic-conditioned soil suspension treatment, and GS represents for unfiltered garlic-conditioned soil suspension treatment.

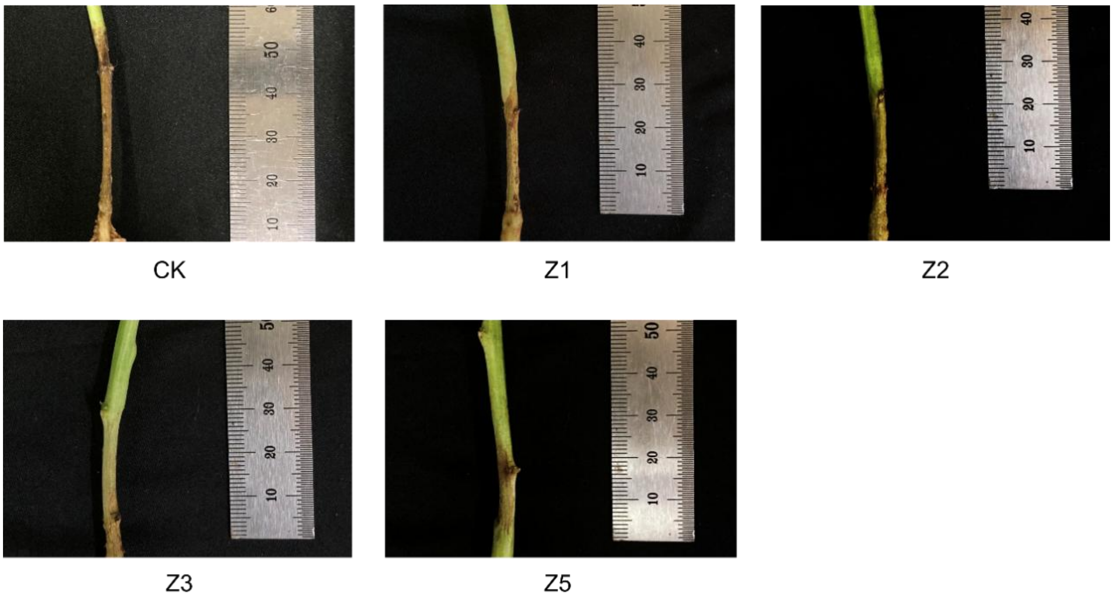

Supplemental Figure 4 Effects of soil suspension from the conditioned **soil with different garlic densities on the lesion expansion of pepper *Phytophthora* blight on stem.**

Z1, Z2, Z3, and Z5 represent 1, 2, 3, and 5 garlic plants per pot, respectively. CK represents control group

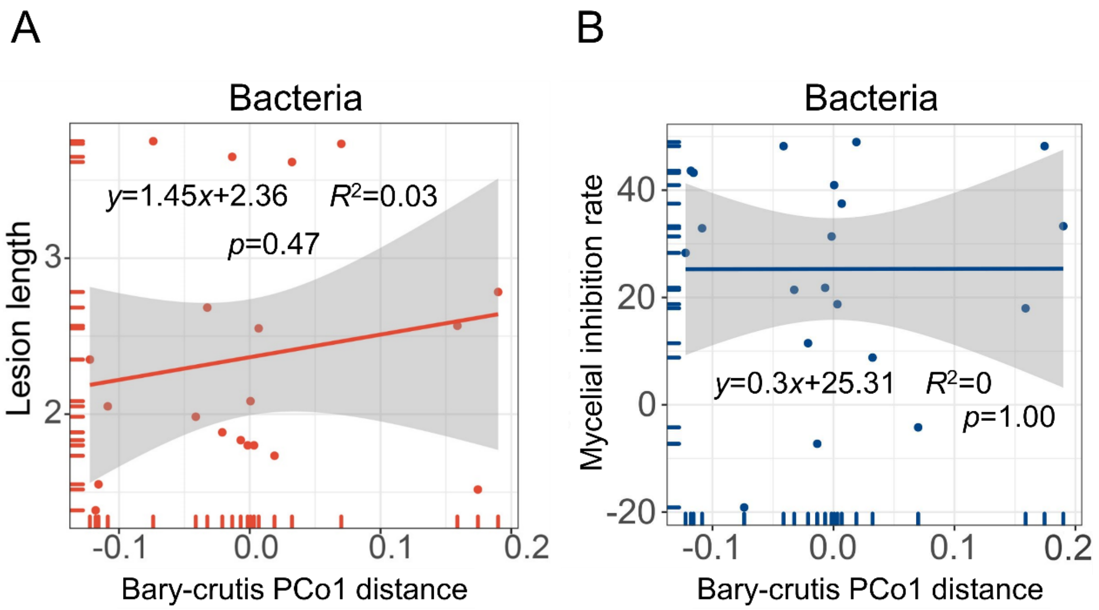

Supplemental Figure 5 Correlation analysis between bacterial community structure (PCo1 axis) at the genus level and lesion length (A) or mycelial inhibition rate (B).

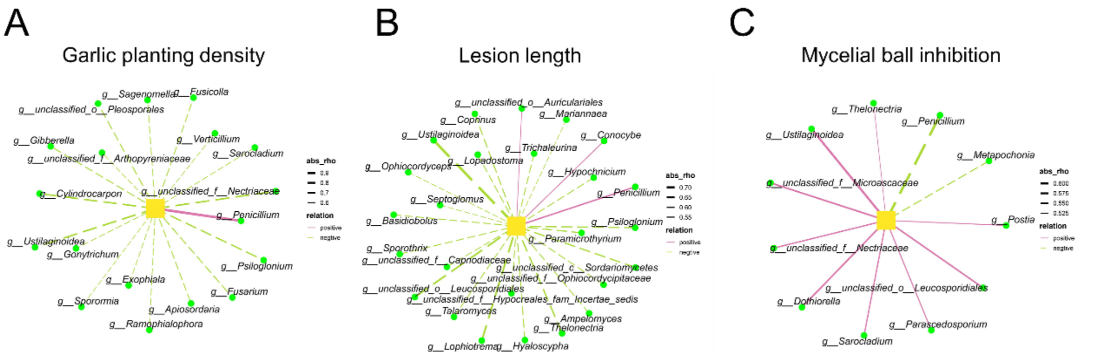

Supplemental Figure 6 Correlation analysis between garlic-conditioned soil fungi and planting density, pepper blight lesion length and *P. capsici* mycelial inhibition.

(A) Correlation analysis between garlic-conditioned soil microorganisms and planting density.

(B) Correlation analysis of garlic-conditioned soil microorganisms and *P. capsici* mycelial inhibition.

(C) Correlation analysis between garlic-conditioned soil microorganisms and pepper *Phytophthora* blight lesion length. The green dashed line represents a negative correlation, and the pink solid line represents a positive correlation

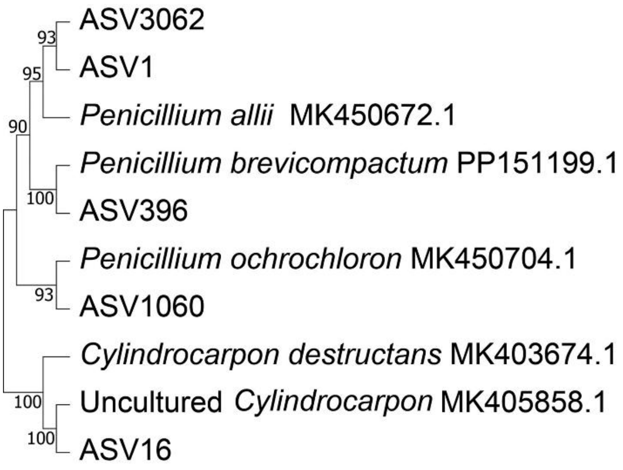

Supplemental Figure 7 Evolutionary tree of *Penicillium* species and ASV level based on ITS.

Bootstrap values based on 1000 replications are shown as percentages at each branch.

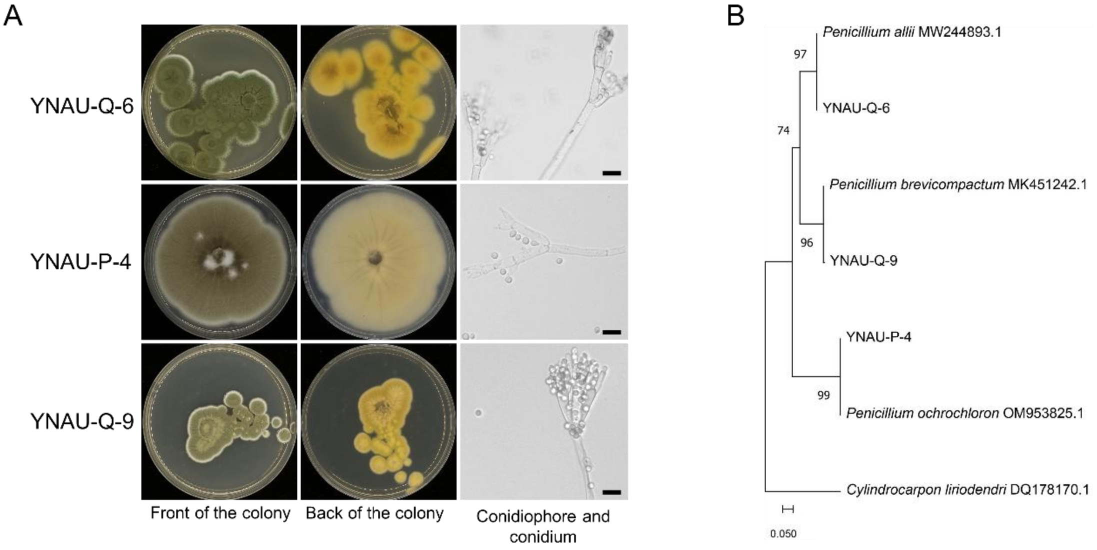

Supplemental Figure 8 Morphological and molecular biological identification of isolated *Penicillium*

(A) Morphological identification of *Penicillium*.

(B) Evolutionary tree of *Penicillium* based on β-tubulin.

Bootstrap values based on 1000 replications are shown as percentages at each branch. Bar = 10 μm.

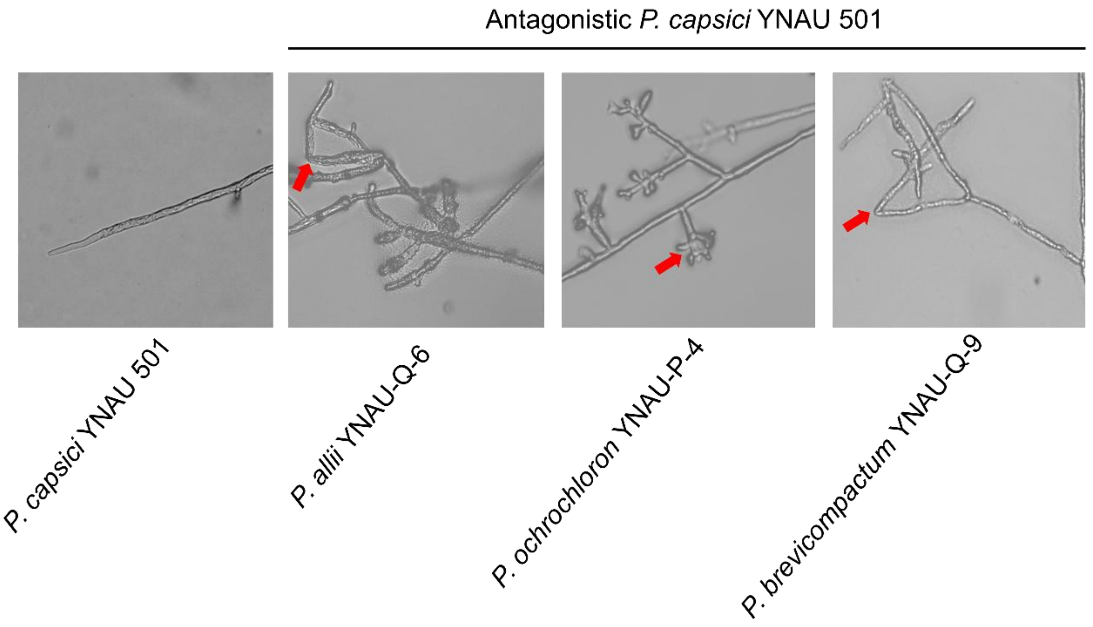

Supplemental Figure 9 Effect of isolated *Penicillium* species on mycelium morphology of *P. capsici.*

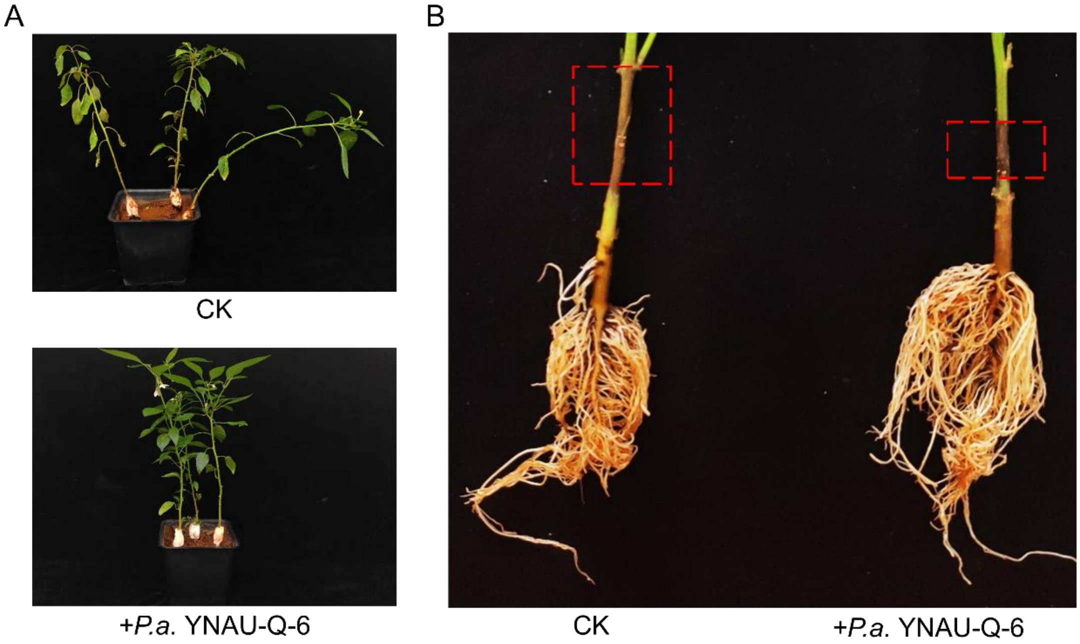

Supplemental Figure 10 Effects of *Penicillium allii* YNAU-Q-6 on pepper *Phytophthora* blight lesion length.

(A) Symptoms of pepper blight under YNAU-Q-6 treatment.

(B) Effects of YNAU-Q-6 on pepper blight lesion length.

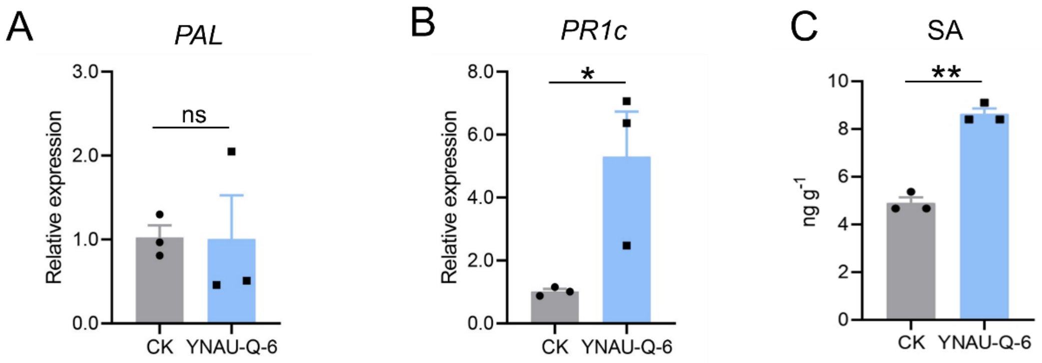

Supplemental Figure 11 The relative expression of resistance genes and salicylic acid contents in pepper shoots induced by *P. allii* YNAU-Q-6.

(A) The relative expression of phenylalanine ammonia-lyase (*PAL*) in pepper shoots.

(B) The relative expression of pathogenesis-related protein 1c (*PR1c*) in pepper shoots.

(C) The salicylic acid (SA) contents in pepper shoots.

Data are expressed as mean ± standard error. An independent sample *t*-test was used for data significance analysis. ** indicates $p < 0.01$, * indicates $p < 0.05$, ns means no significance.

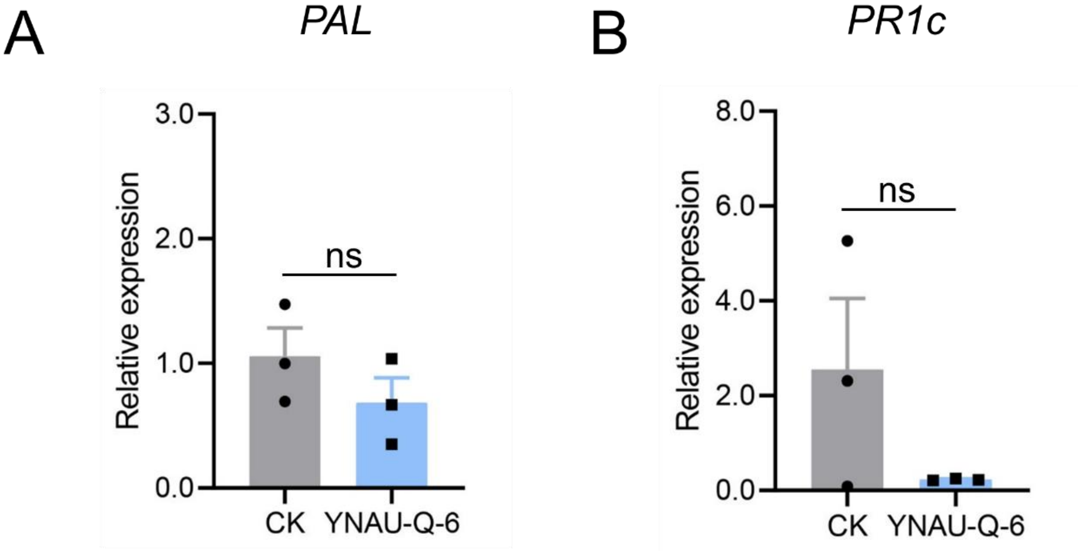

Supplemental Figure 12 The relative expression of resistance genes in garlic seedlings induced by *P. allii* YNAU-Q-6.

(A) The relative expression of phenylalanine ammonia-lyase (*PAL*) in garlic seedlings.

(B) The relative expression of pathogenesis-related protein 1c (*PR1c*) in garlic seedlings.

Data are expressed as mean ± standard error. An independent sample *t*-test was used for data significance analysis. ns means no significance.

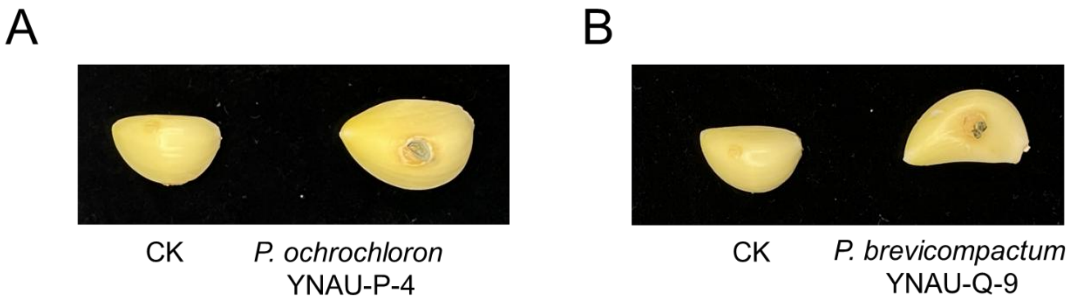

Supplemental Figure 13 Effects of *P. ochrochloron* YNAU-P-4 (A) and *P. brevicompactum* YNAU-Q-9 (B) on garlic cloves.

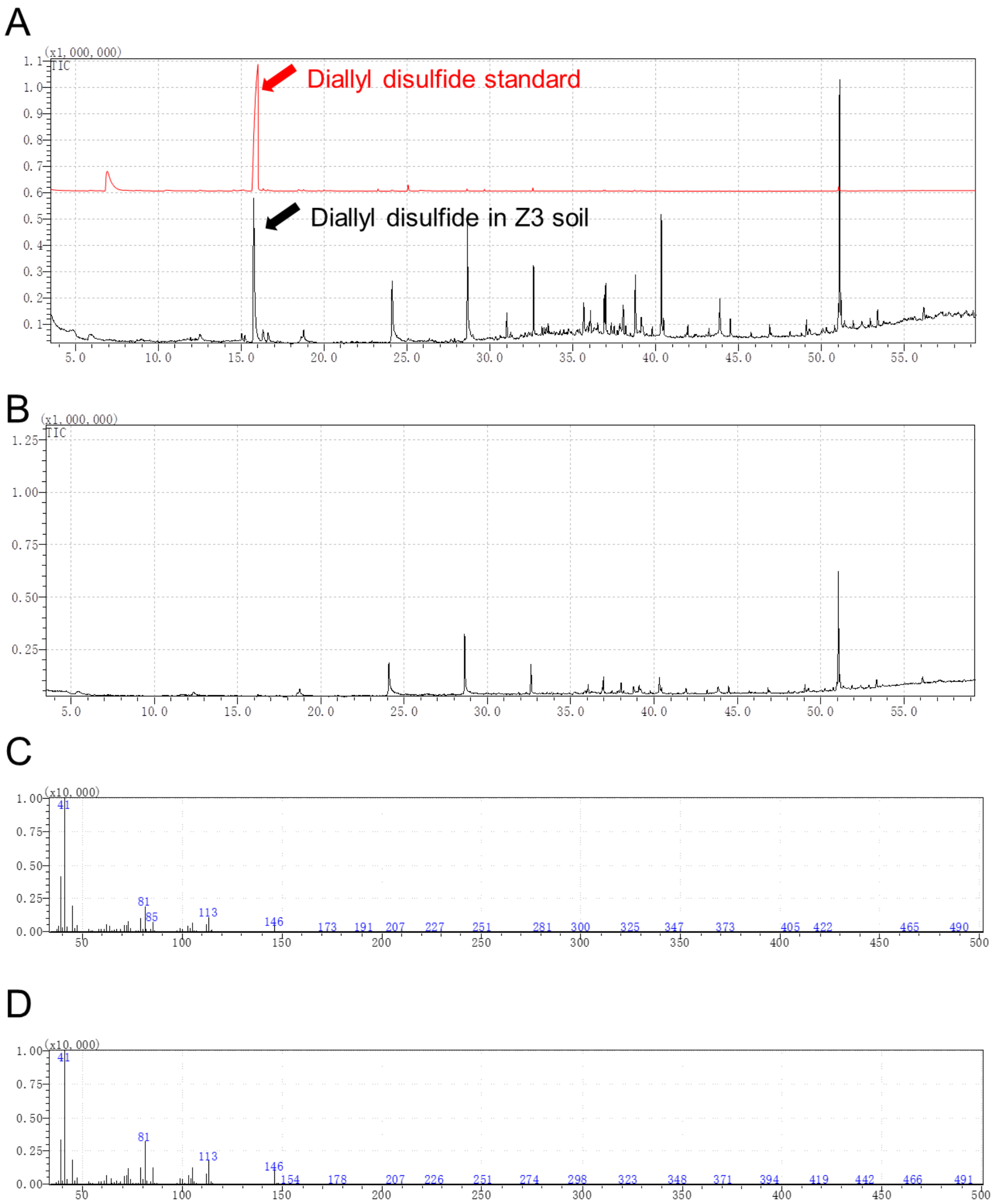

Supplemental Figure 14 Volatile compounds in pot-grown garlic-conditioned soil (Z3 treatment) by GC-MS.

(A) Total ion current (TIC) chromatograms of garlic-conditioned soil.

(B) Total ion current (TIC) chromatograms of CK soil.

(C) The mass spectrum of diallyl disulfide in garlic-conditioned soil.

(D) The mass spectrum of standard diallyl disulfide.

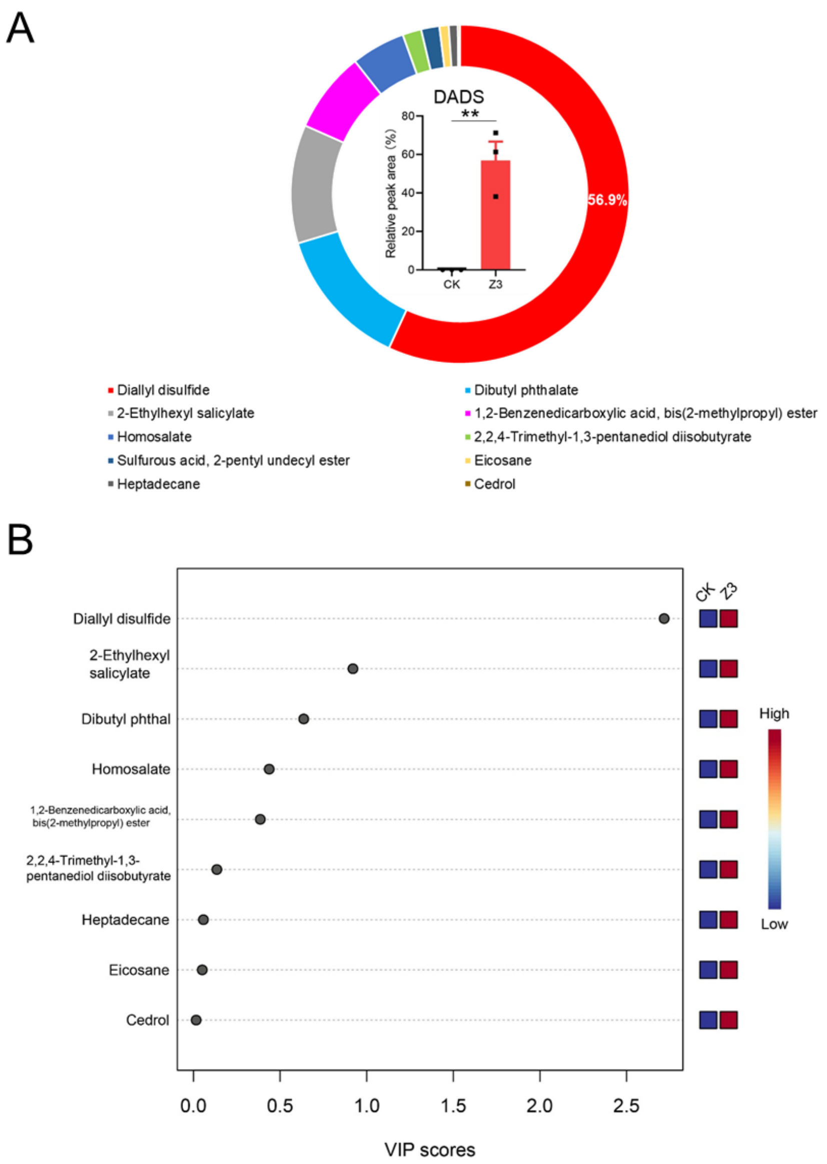

Supplemental Figure 15 Detection of volatile compounds from garlic-conditioned soil in the pot

(A) Relative peak area ratio of volatile compounds in Z3 treatment.

(B) Variable importance in projection analysis (VIP) of volatile compounds between CK and Z3 treatment.

Data are expressed as mean ± standard error. An independent sample *t*-test was used for data significance analysis. ** indicates $p < 0.01$.

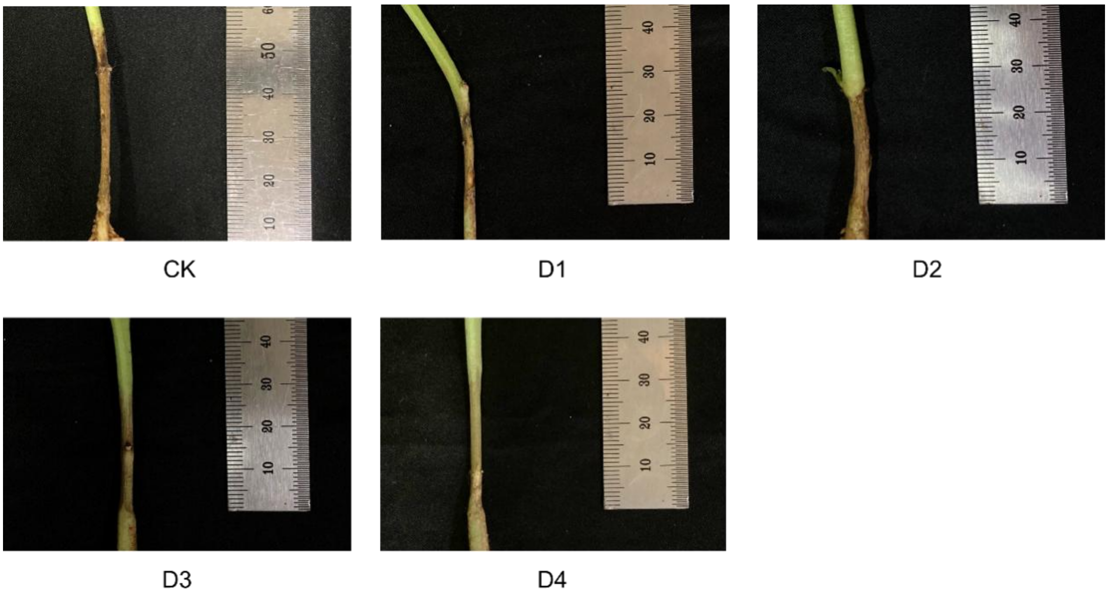

Supplemental Figure 16 Effects of soil microbiome shaped by exogenous DADS with different concentrations on pepper blight expansion.

D1, D2, D3, and D4 represent soil DADS concentrations of 13.7, 27.3, 54.7, and 109.4 μmol kg^{-1}, respectively. CK represents control group.

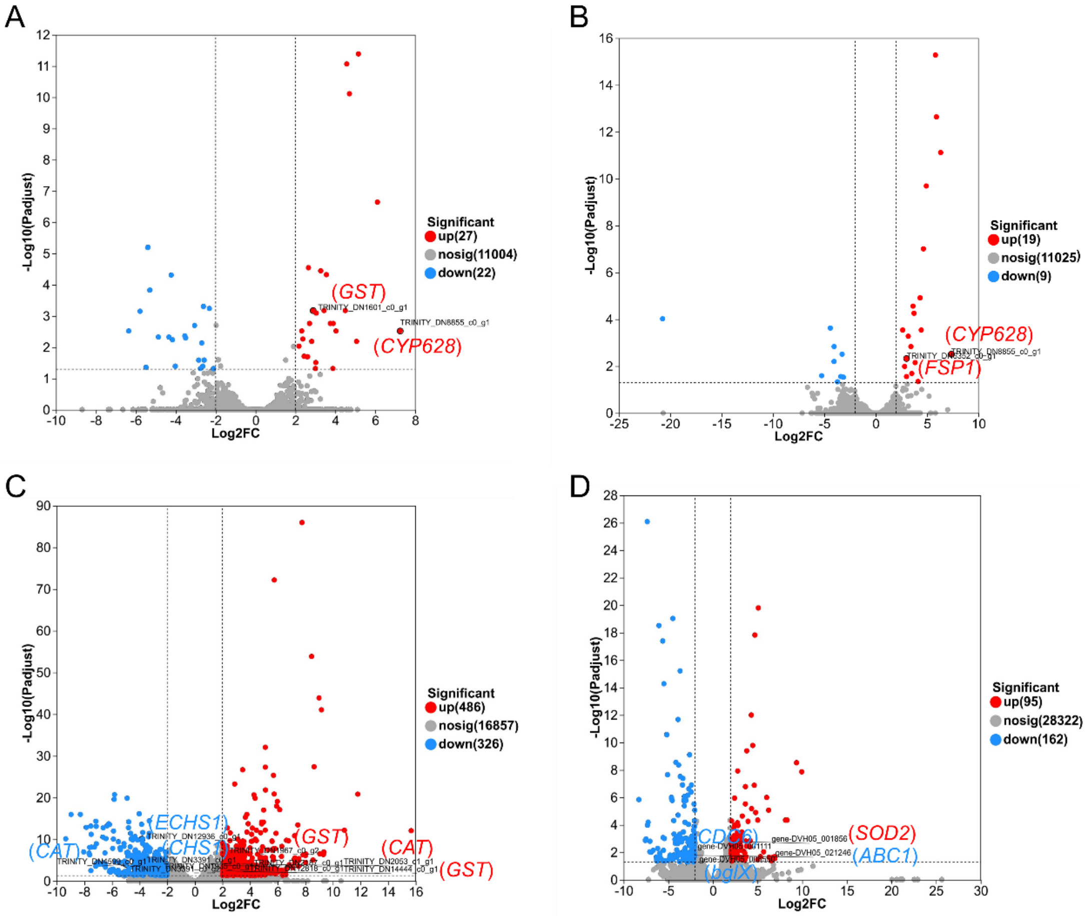

Supplementary Figure 17 Volcano plot of differentially expressed genes (DEGs) from different microorganisms after DADS treatment

(A) DEGs of ***P. allii*** YNAU-Q-6 treated with 13.7 μM DADS.

(B) DEGs of ***P. allii*** YNAU-Q-6 treated with 273.5 μM DADS.

(C) DEGs of ***C. destructans*** YNAU-RS-6 treated with 273.5 μM DADS.

(D) DEGs of ***P. capsici*** YNAU-501 treated with 683.7 μM DADS.

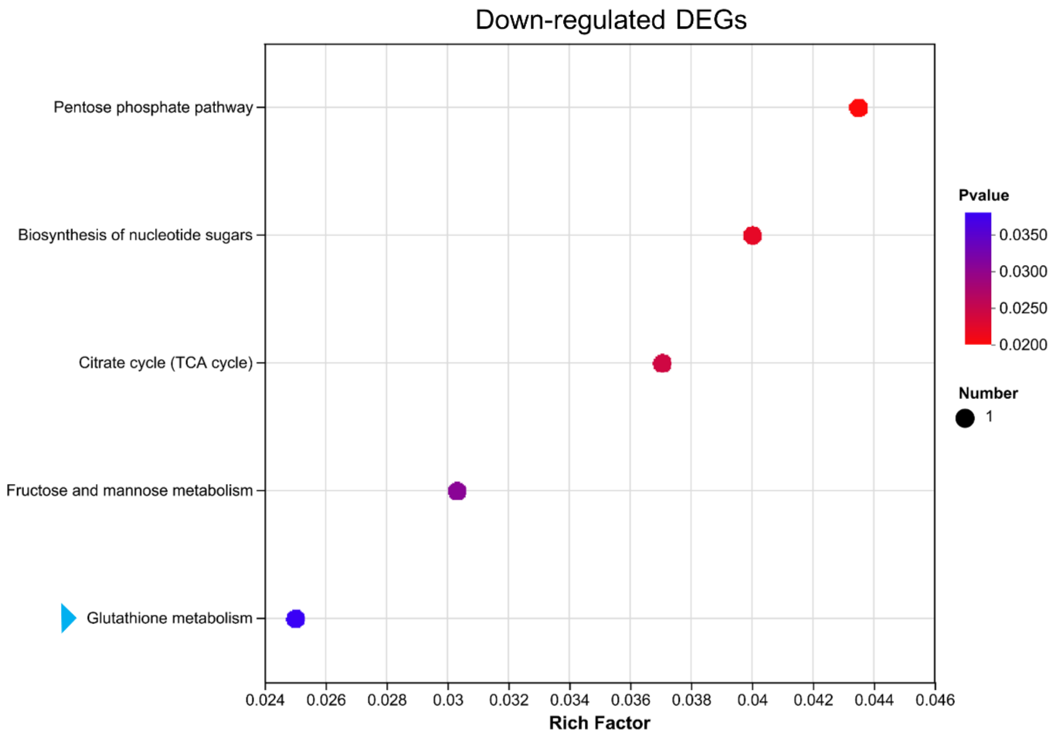

Supplemental Figure 18 KEGG pathway enrichment analysis of *P. allii* YNAU-Q-6 after 13.7 μM DADS treatment.

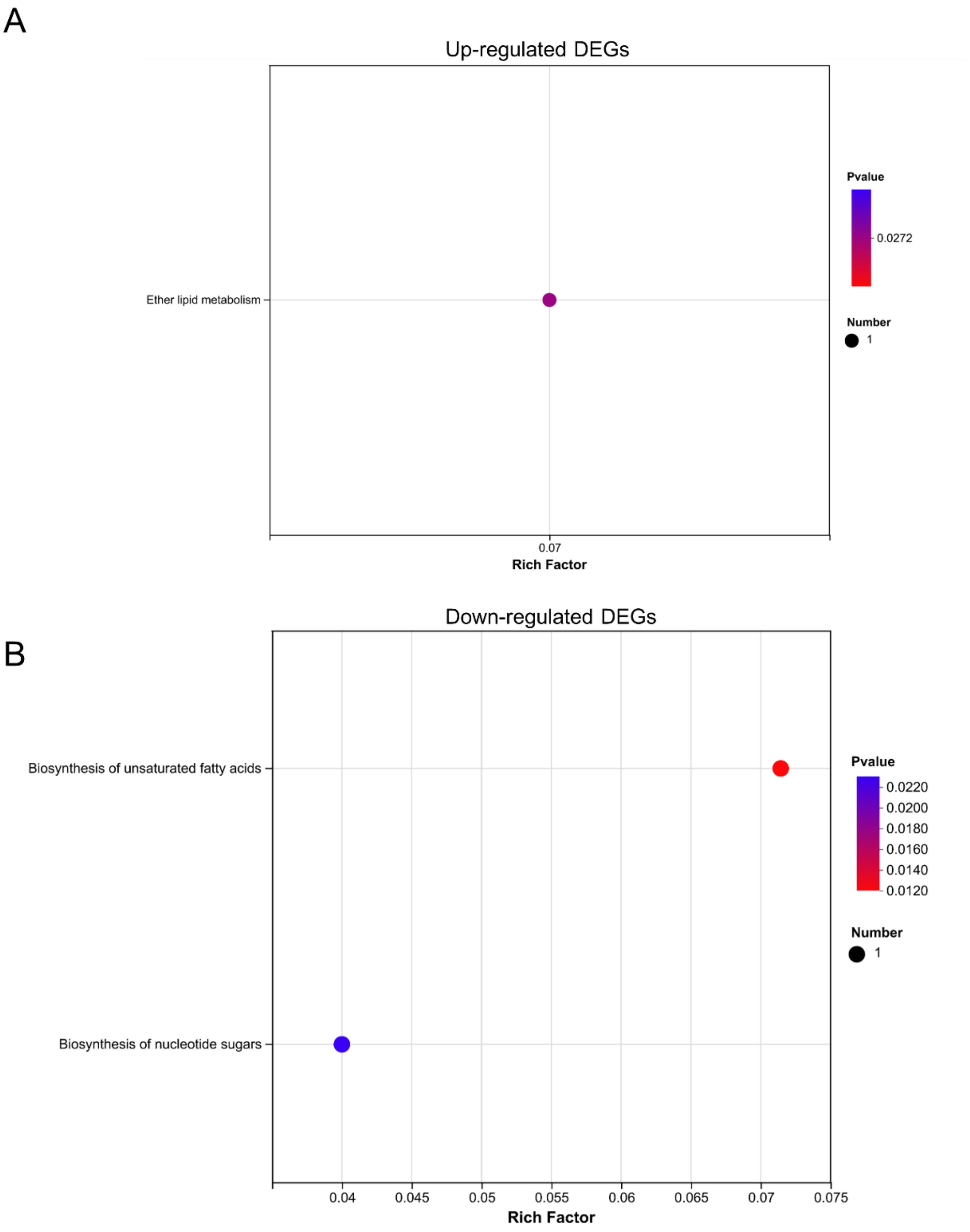

Supplemental Figure 19 KEGG pathway enrichment analysis of *P. allii* YNAU-Q-6 after 273.5 µM DADS treatment.

(A) KEGG pathway enrichment analysis of up-regulated DEGs.

(B) KEGG pathway enrichment analysis of down-regulated DEGs.

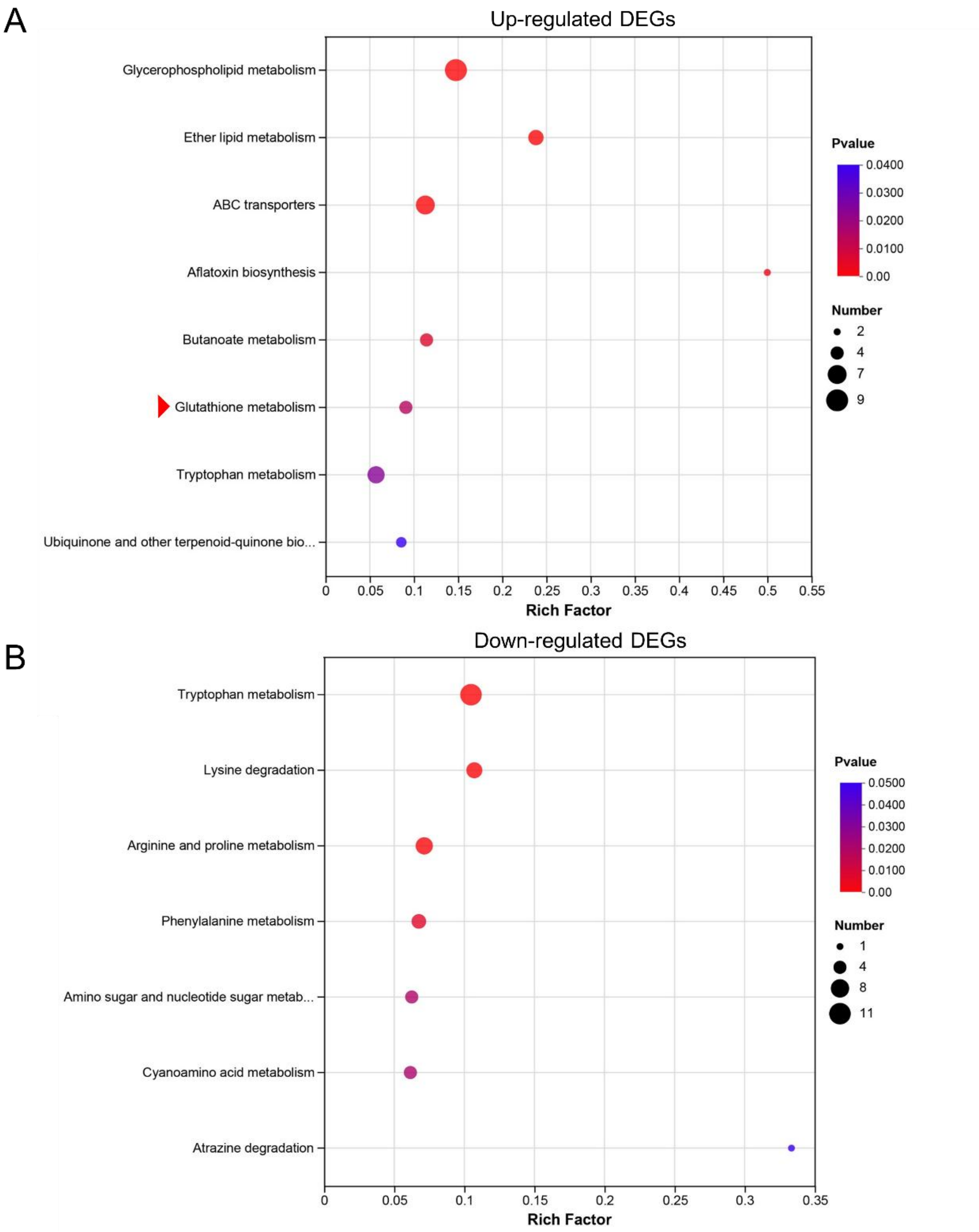

Supplemental Figure 20 KEGG pathway enrichment analysis of *C. destructans* YNAU-RS-6 after 273.5 μM DADS treatment.

(A) KEGG pathway enrichment analysis of up-regulated DEGs.

(B) KEGG pathway enrichment analysis of down-regulated DEGs.

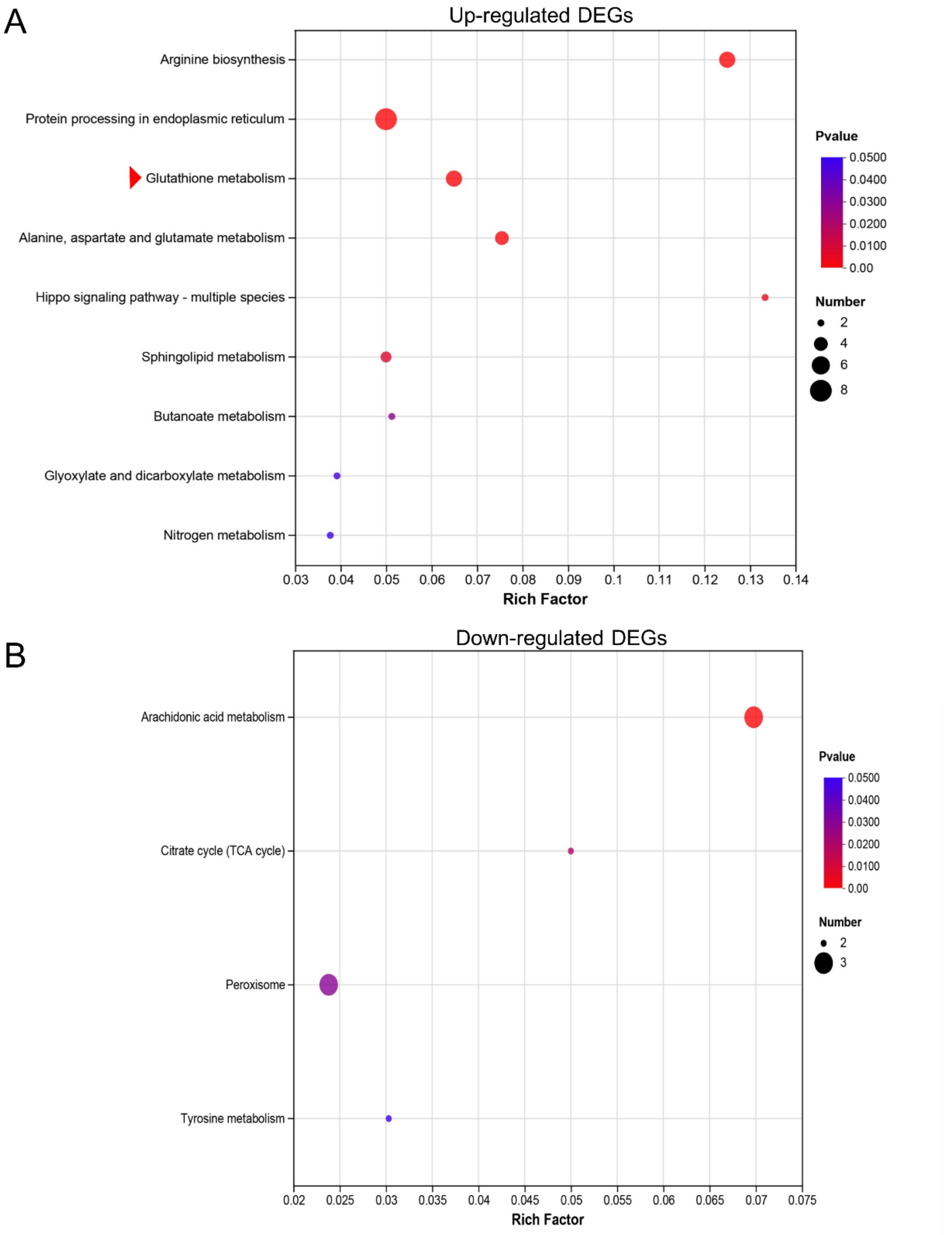

Supplementary Figure 21 KEGG pathway enrichment analysis of *P. capsici* YNAU-501 after 683.7 μM DADS treatment.

(A) KEGG pathway enrichment analysis of up-regulated DEGs.

(B) KEGG pathway enrichment analysis of down-regulated DEGs.

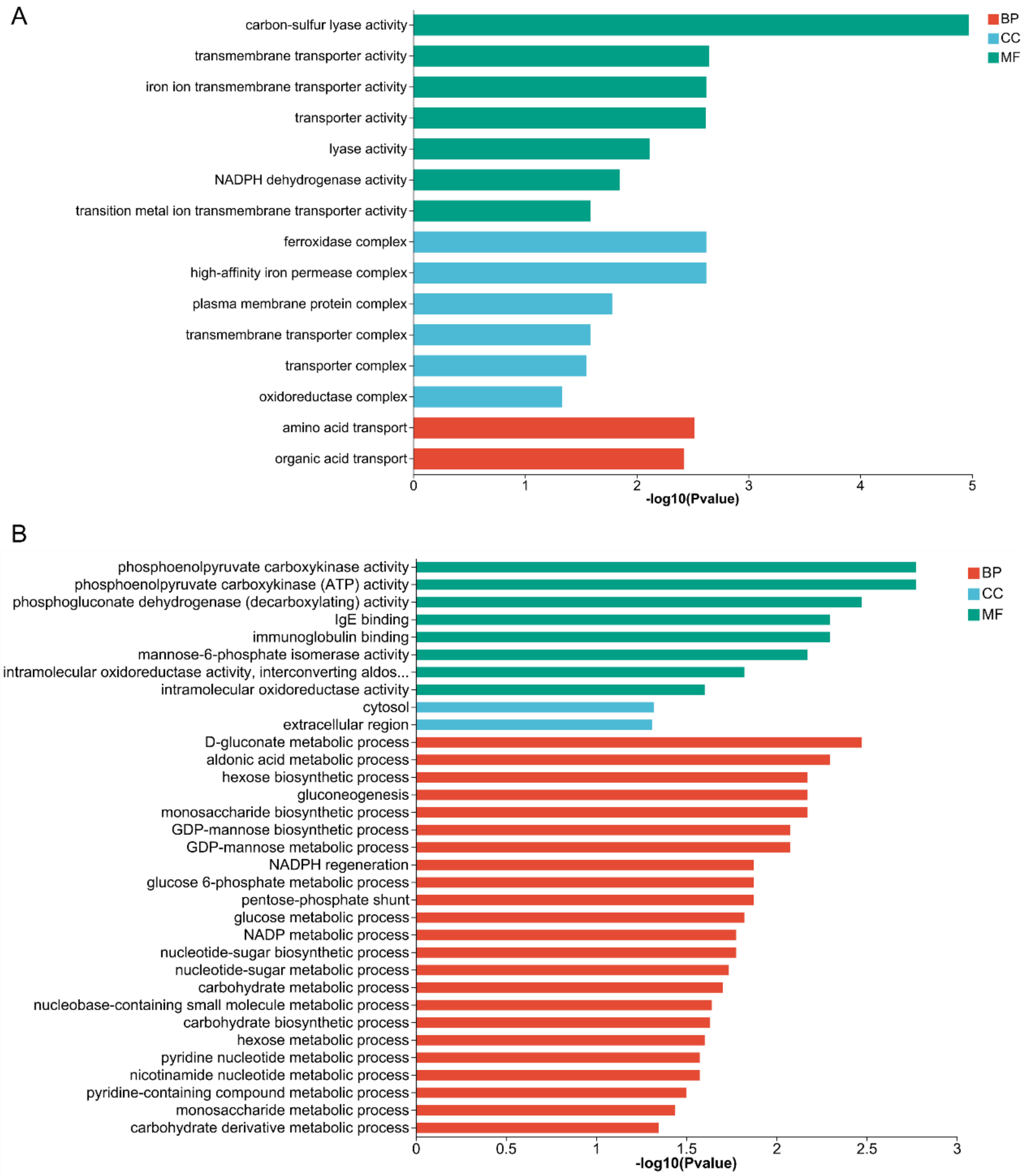

Supplemental Figure 22 GO pathway enrichment analysis of *P. allii* YNAU-Q-6 after 13.7 μM DADS treatment.

(A) GO pathway enrichment analysis of up-regulated DEGs.

(B) GO pathway enrichment analysis of down-regulated DEGs.

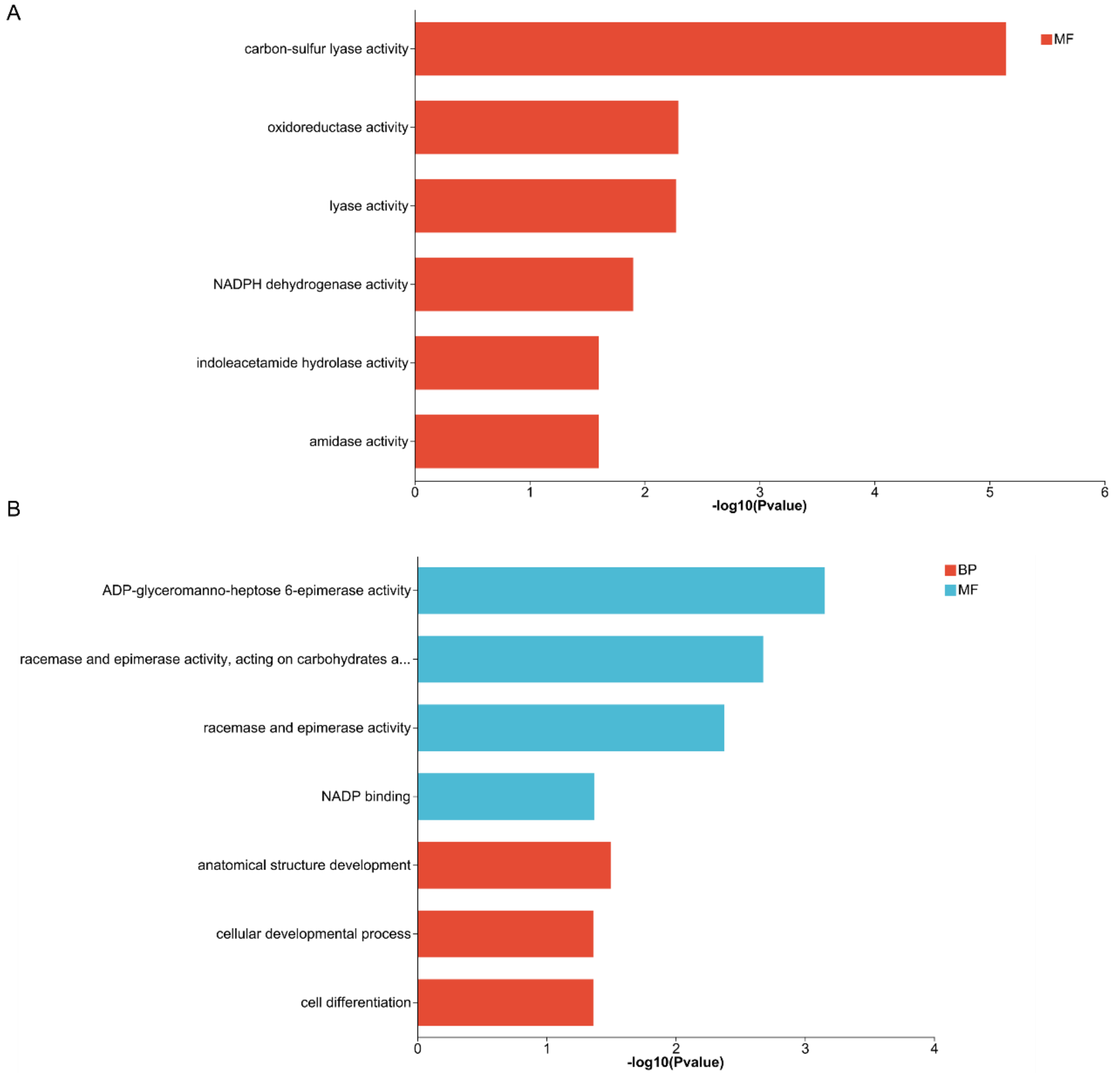

Supplemental Figure 23 GO pathway enrichment analysis of *P. allii* YNAU-Q-6 after 273.5 µM DADS treatment.

(A) GO pathway enrichment analysis of up-regulated DEGs.

(B) GO pathway enrichment analysis of down-regulated DEGs.

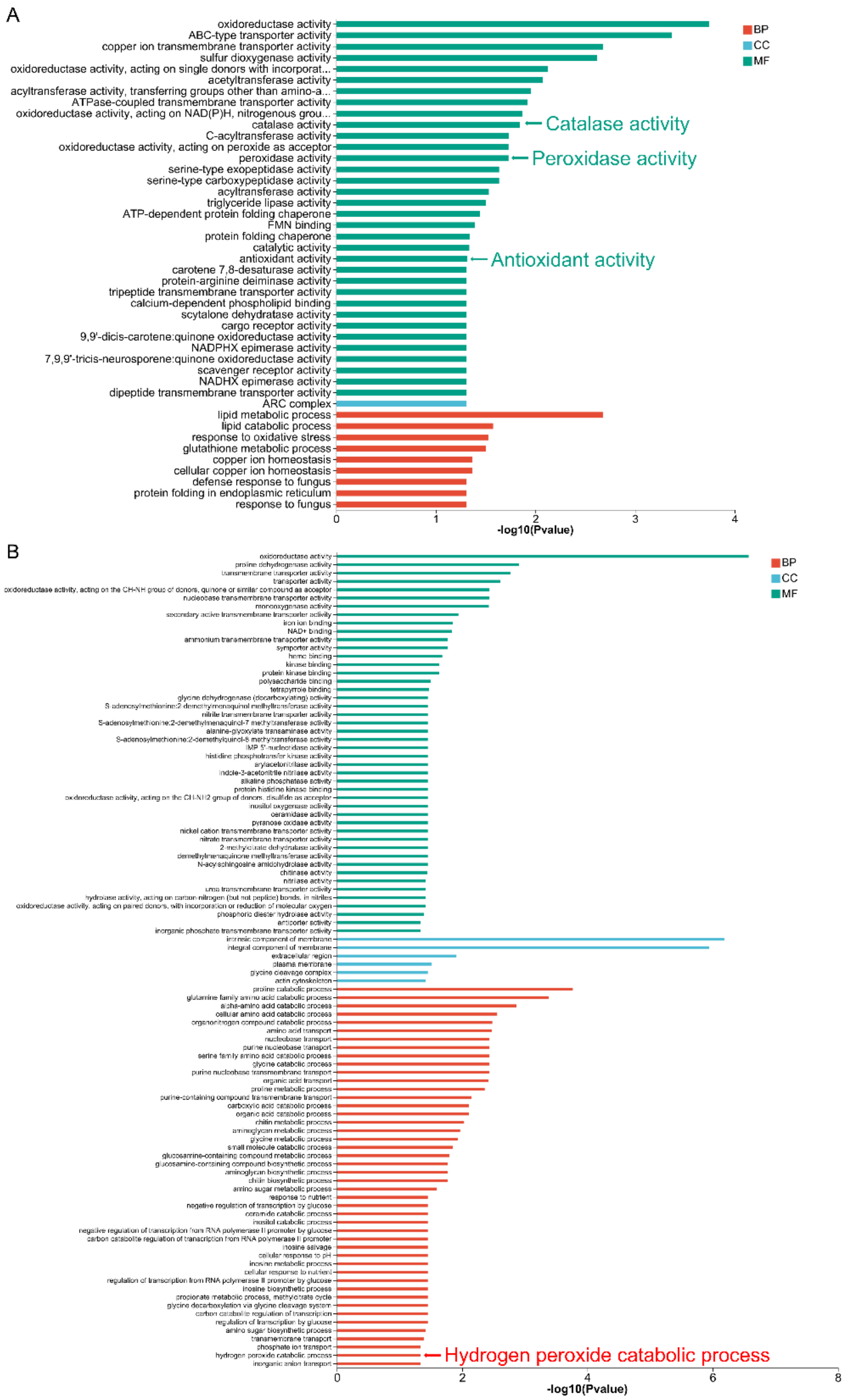

Supplemental Figure 24 GO pathway enrichment analysis of *C. destructans* YNAU-RS-6 after 273.5 µM DADS treatment.

(A) GO pathway enrichment analysis of up-regulated DEGs.

(B) GO pathway enrichment analysis of down-regulated DEGs.

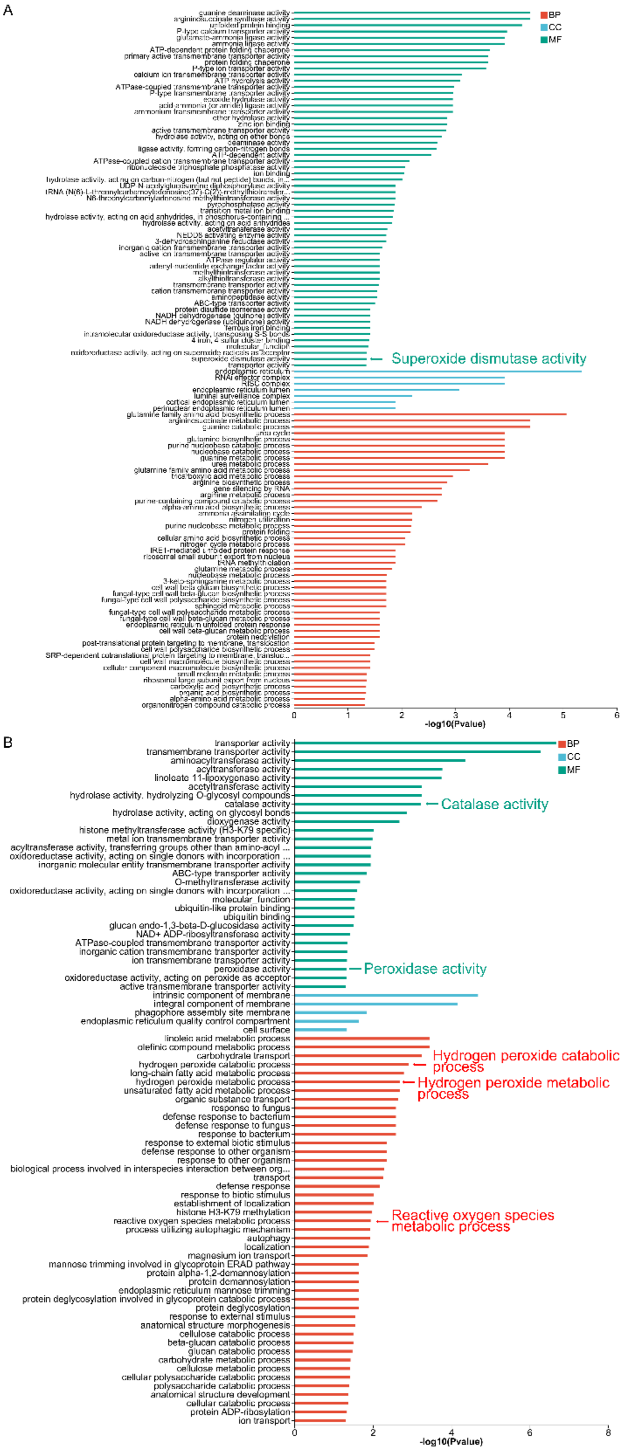

Supplementary Figure 25 GO pathway enrichment analysis of *P. capsici* YNAU-501 683.7 µM after DADS treatment.

(A) KEGG pathway enrichment analysis of up-regulated DEGs.

(B) KEGG pathway enrichment analysis of down-regulated DEGs.

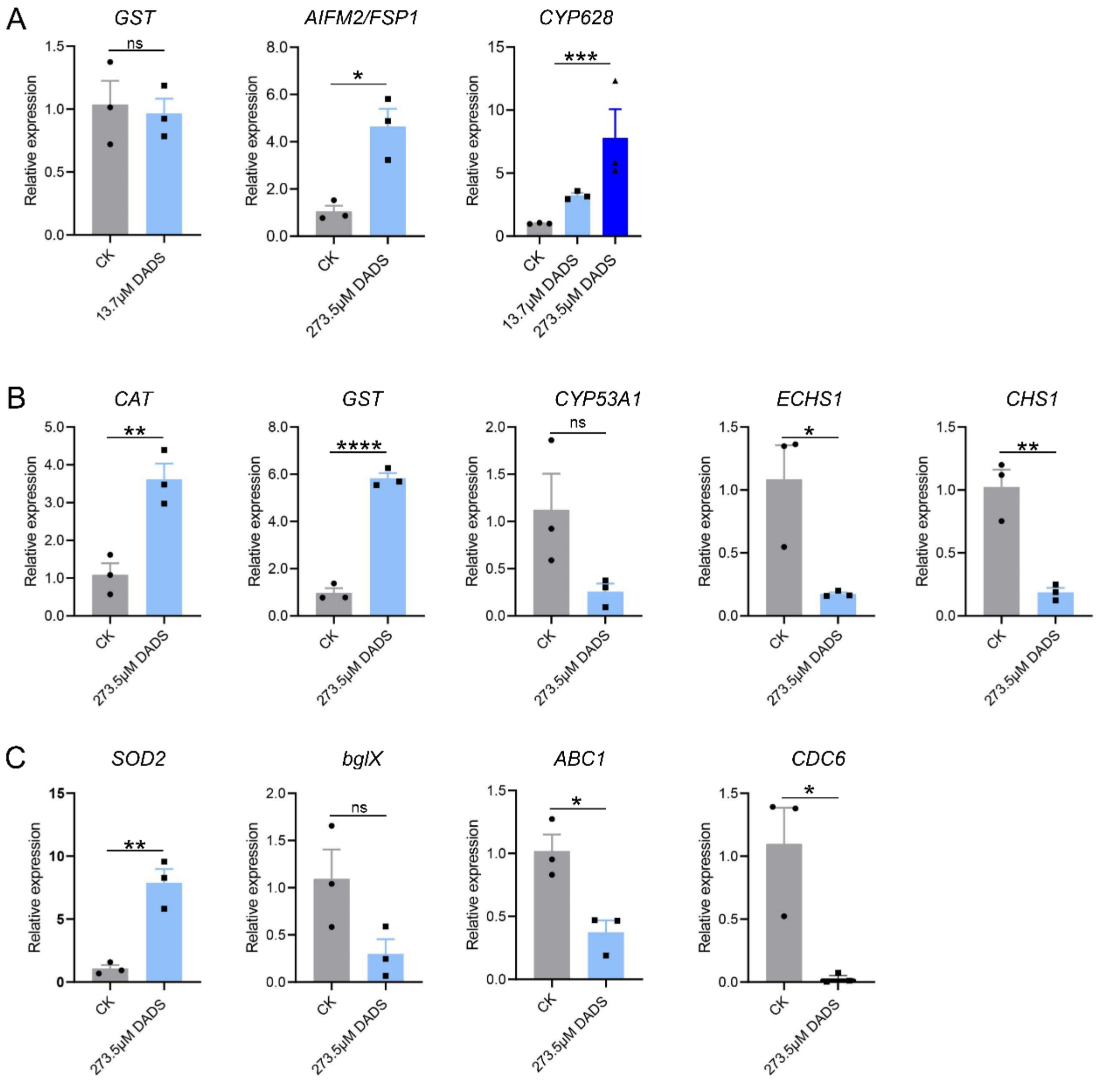

Supplemental Figure 26 Validation of differentially expressed genes using RT-qPCR.

(A) RT-qPCR detection of DEGs in *P. allii* YNAU-Q-6.

(B) RT-qPCR detection of DEGs in *C. destructans* YNAU-RS-6.

(C) RT-qPCR detection of DEGs in *P. capsici* YNAU-501.

Data are expressed as mean ± standard error. An independent sample *t*-test was used for data significance analysis. * indicates $p < 0.05$, ** indicates $p < 0.01$, *** indicates $p < 0.001$, **** indicates $p < 0.0001$, ns means no significance.

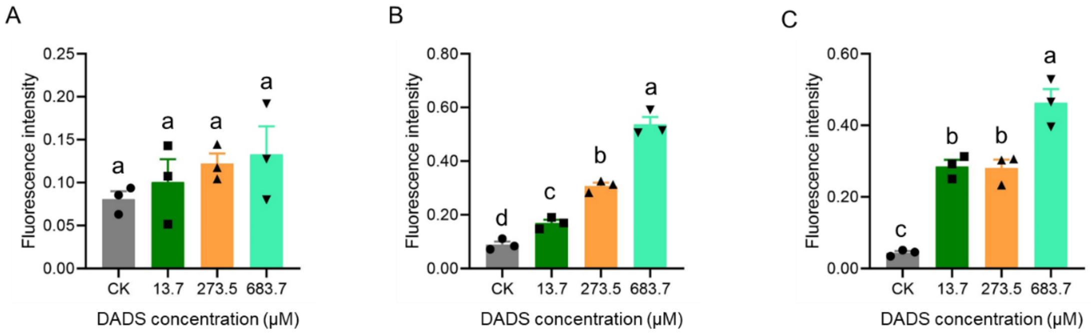

Supplemental Figure 27 ROS quantitative detection of three isolates induced by DADS

(A) Fluorescence intensity detection in *P. allii* YNAU-Q-6.

(B) Fluorescence intensity detection in *C. destructans* YNAU-RS-6.

(C) Fluorescence intensity detection in *P. capsici* YNAU-501.

Data are expressed as mean ± standard error. Different lowercase letters indicate significant differences between treatments ($p < 0.05$, according to ANOVA followed by Duncan's multiple range test).

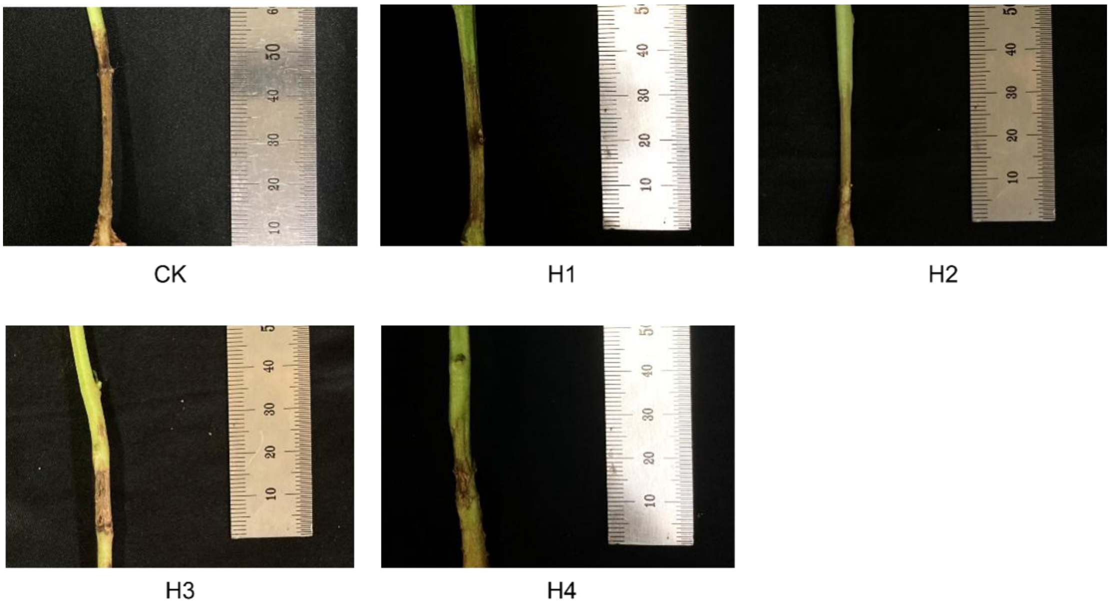

Supplemental Figure 28 Effects of soil microbiome shaped by exogenous H_2O_2 with different concentrations on pepper blight expansion.

H1, H2, H3, and H4 represent soil H_2O_2 concentrations of 85.9, 171.7, 343.4, and 686.9 μmol kg^{-1}, respectively. CK represents control group.

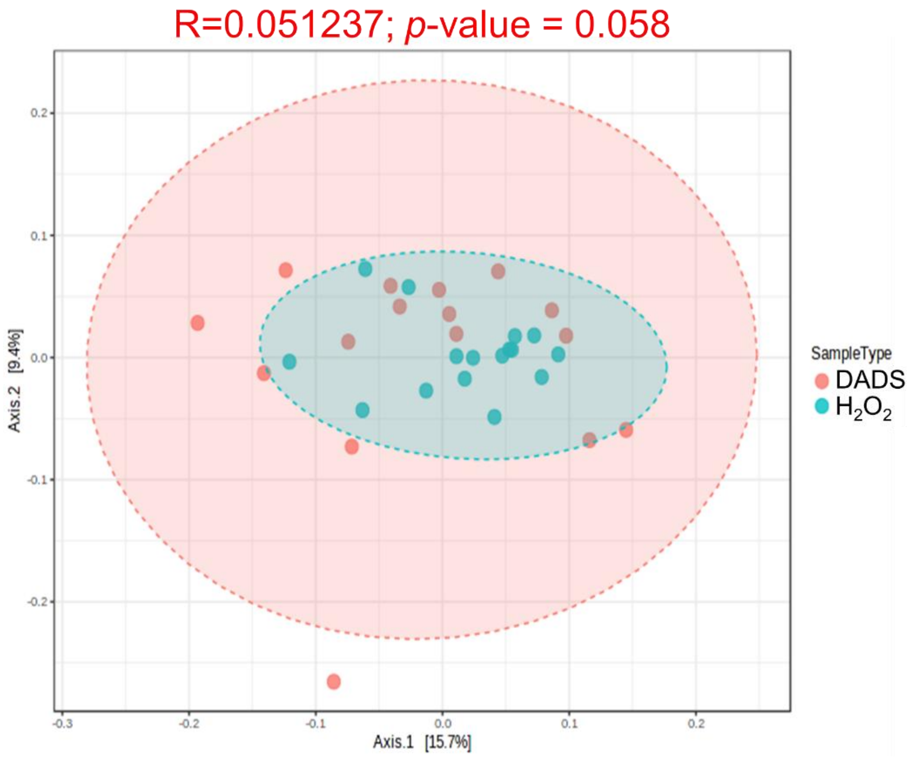

Supplemental Figure 29 ANOSIM analysis of fungal communities treated with DADS and H_2O_2.

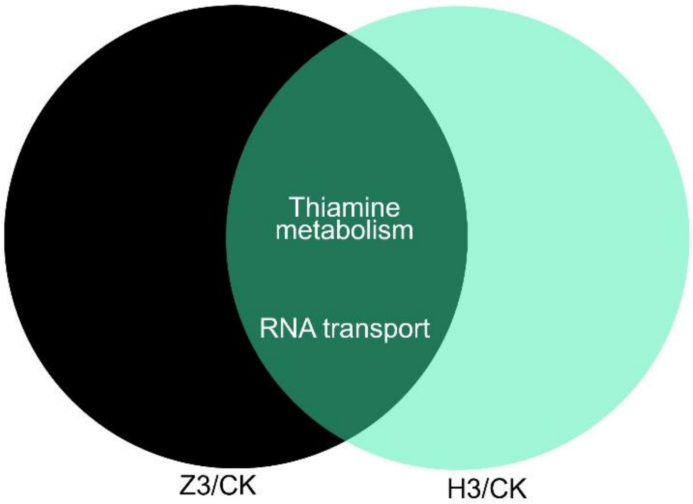

Supplemental Figure 30 Venn diagram of co-variant pathways in Z3 and H3 metagenomes

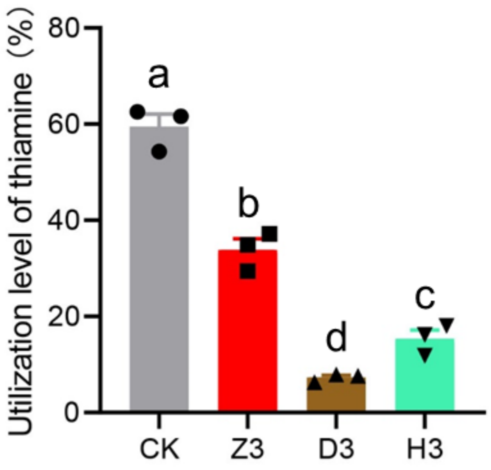

Supplemental Figure 31 Thiamine utilization level of soil microorganism in three treatments. Data are expressed as mean ± standard error. Different lowercase letters indicate significant differences between treatments ($p < 0.05$, according to ANOVA with Duncan's multiple range test). Z3 represents three garlic plants per pot**,** D3 represents DADS treatment at 54.7 μmol kg^{-1} soil, and H3 represents H_2O_2 treatment at 343.4 μmol kg^{-1}.

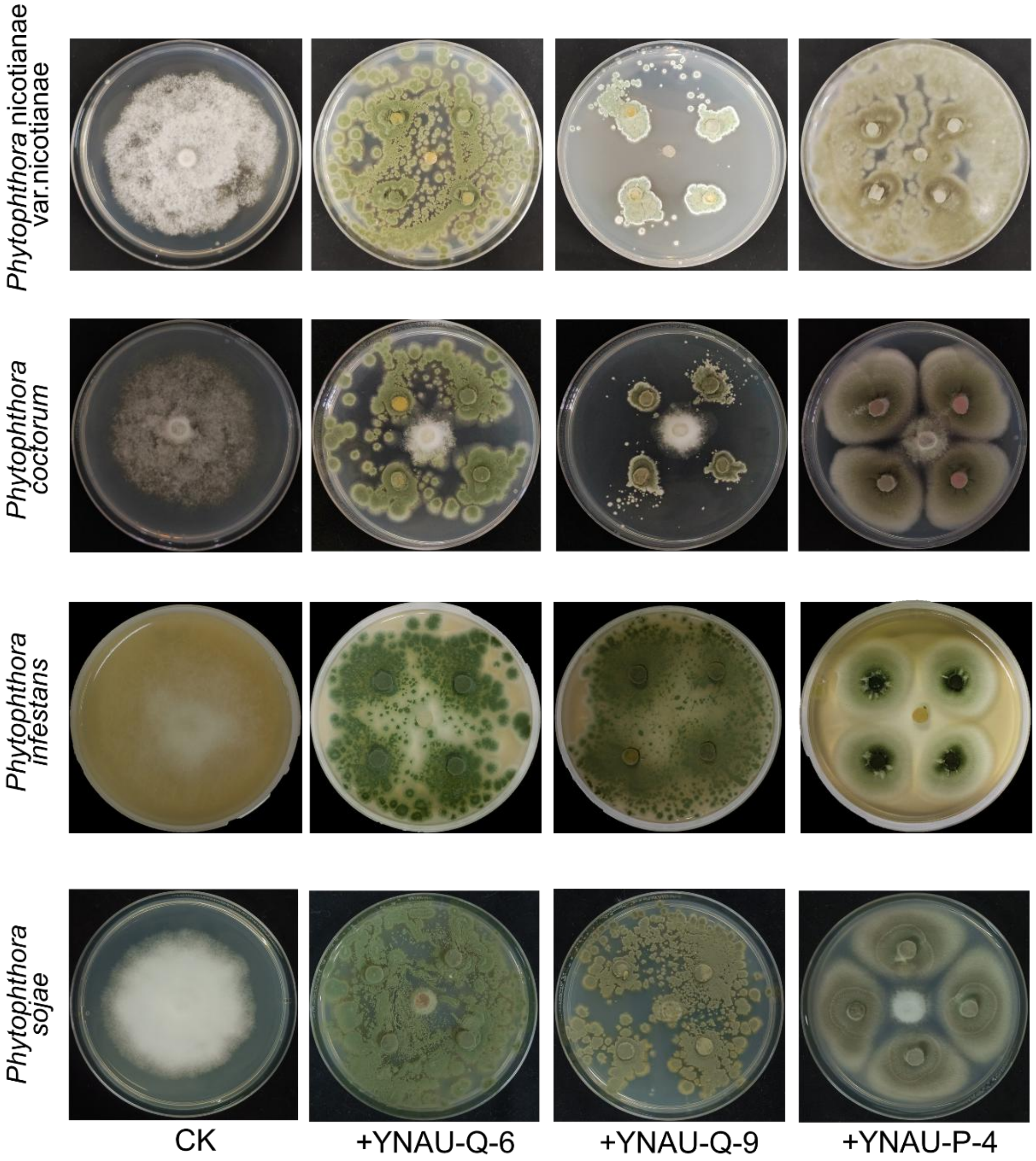

Supplemental Figure 32 Antagonistic effects of three *Penicillium* spp. isolated from the conditioned soil of garlic against different *Phytophthora* spp.

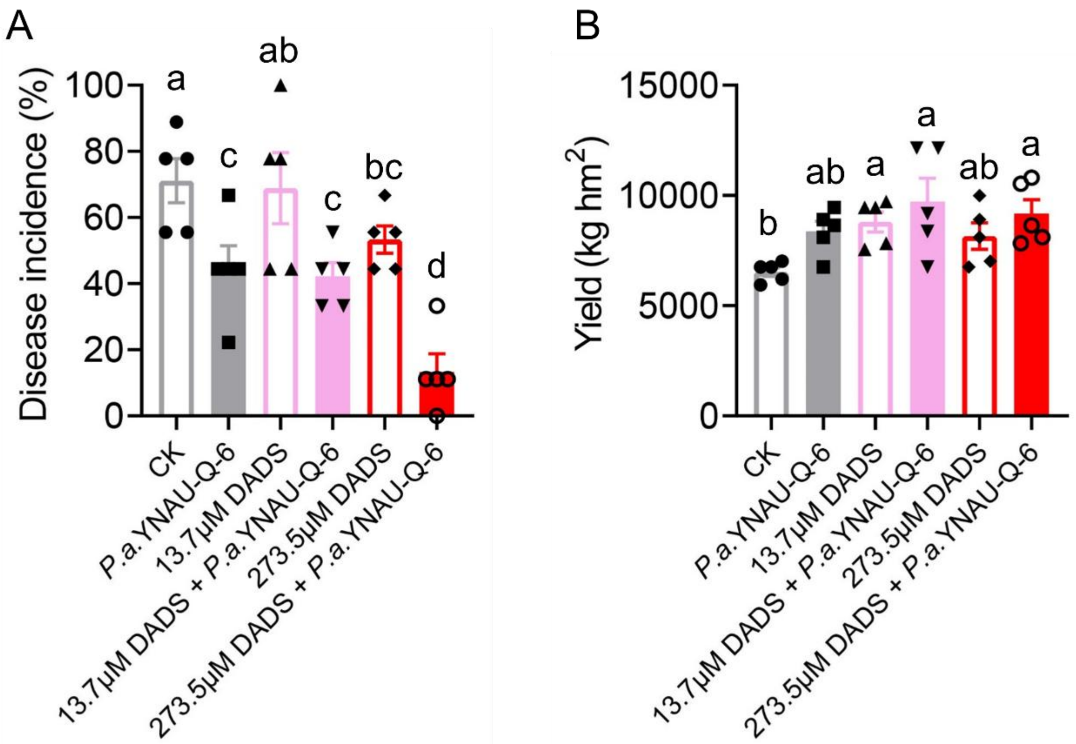

Supplemental Figure 33 Effects of application of *P. allii* YNAU-Q-6 and DADS on the incidence of tobacco black shank and soybean yield.

(A) Incidence of tobacco black shank.

(B) Soybean yield.

Data are expressed as mean ± standard error (n = 5). Different lowercase letters indicate significant differences between treatments (p < 0.05, according to ANOVA followed by Duncan's multiple range test).

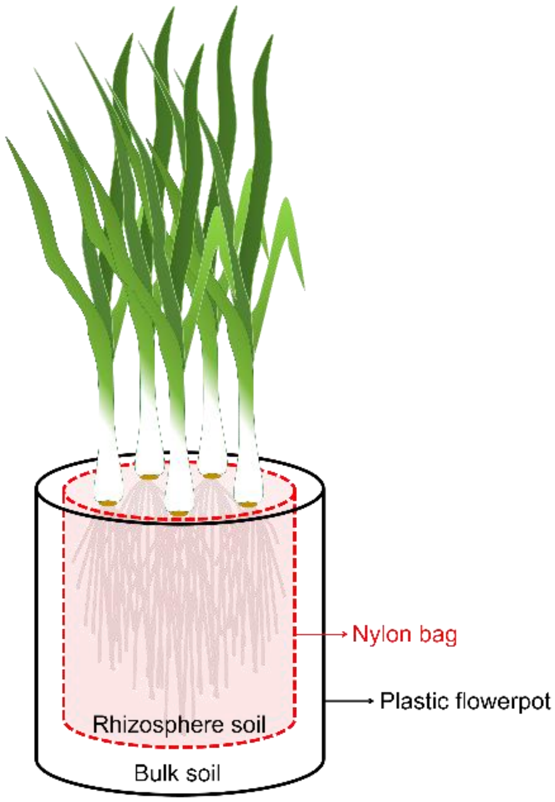

Supplemental Figure 34 Garlic root bag potting experiment device.

Supplemental References

Anisimova, O.K., Shchennikova, A.V., Kochieva, E.Z., and Filyushin, M.A. (2021). Pathogenesis-related genes of PR1, PR2, PR4, and PR5 families are involved in the response to Fusarium infection in garlic (*Allium sativum* L.). Int. J. Mol. Sci. **22**:6688. https://doi.org/10.3390/ijms22136688.

Diao, X.C., Wang, S.B., Diao, W.P., Pan, B.G., Ge, W., and Gao, Q.H. (2019). Cloninig and expression analysis of CaWRKY8 generin pepper under stresses. Acta Bot. Boreal.-Occident. Sin. **39**: 0210-0217. https://doi.org/10.7606/j.esn.1000-4025.

Ding, H.Y., Ali, A., and Cheng, Z.H. (2018). Dynamics of a soil fungal community in a three-year green garlic/cucumber crop rotation system in Northwest China. Sustainability **10**:1391. https://doi.org/10.3390/su10051391.

Guo, P.T., Wu, X.T., Lai, R.Q., Chen, Z.H., Chang, Y., and Bai, J.J. (2019) Allelopathic effects of garlic root exudates on different varieties of flue-cured tobacco. Wuyi Sci. J. **35**:97-102. https://doi: 10.15914 /j.cnki.wykx.2019.02.05.

Luo, L.F., Wang, Z.P., Yan, X.B., Ye, C., Hao, J.J., Liu, X.L., Zhu, S.S., and Yang, M. (2025). Diversified *Alternaria* pathogenicity alters plant-soil feedbacks through leaf-root-microbiome dynamics in agroforestry systems. Hortic. Res. **12**:uhaf137. https://doi.org/10.1093/hr/uhaf137.

Liu, H.J., Wu, J.Q., Su, Y.W., Li. Y,B., Zuo, D.H., Liu, H.B., Liu, Y.X., Mei, X.Y., Huang, H.C., Yang, M., et al. (2021). Allyl isothiocyanate in the volatiles of *Brassica juncea* inhibits the growth of root rot Pathogens of *Panax notoginseng* by inducing the accumulation of ROS. J. Agric. Food Chem. **69**:13713-13723. https://doi.org/10.1021/acs.jafc.1c05225.

Nie, H.L., Huang, S.H., Yang, J.F., Yang Y.M., Zhang J., Yang, H.M., Yang, W.F., Xing, S,L., Jia, L.L., and Yue, Z.L. (2023). Meta analysis of the effects of foliar Se application on grain yield, protein content, and Se accumulation of winter wheat. Chin. J. Eco-Agric. **31**:1997−2010. https://doi.org/10.12357/cjea.20230229.

Tuan, P.A., Park, N.I., Li, X., Xu, H., Kim, H.H., and Park, S.U. (2010). Molecular cloning and characterization of phenylalanine ammonia-lyase and cinnamate 4-hydroxylase in the phenylpropanoid biosynthesis pathway in garlic (*Allium sativum*). J. Agric. Food Chem. **58**:10911–10917. https://doi.org/10.1021/jf1021384.

Visagie, C.M., Houbraken, J., Frisvad, J.C., Hong, S.B., Klaassen, C.H. W., Perrone, G., and Samson, R.A. (2014). Identification and nomenclature of the genus *Penicillium*. Stud. Mycol. **78**:343-371. https://dx.doi.org/10.1016/j.simyco.2014.09.001.

Wu, J.Q., Liu, J.Y., Sun, J.W., Liu, Y.P., He, T., Zhao, J., Mei, X.Y., Liu, Y.X., Yang, M., and Zhu, S.S. (2024). Diallyl trisulfide acts as a soil disinfestation against the *Ilyonectria destructans* through inducing the burst of reactive oxygen species. J. Agric. Food Chem. **72**:9669-9679. https://doi.org/10.1021/acs.jafc.4c01422.

Yu, B.J., Li, J.L, G. Moussa, M.G., Wang, W.C., Song, S.S., Xu, Z.C., Shao, H.F., Huang, W.X., Yang, Y.X., Han, D., et al. (2024). Molybdenum inhibited the growth of *Phytophthora nicotiana* and improved the resistance of *Nicotiana tabacum* L. against tobacco black shank. Pestic. Biochem. Phys. **199**:105803-105815. https://doi.org/10.1016/j.pestbp.2024.105803.

Zhao, Z.X., Yan, W.R., Wang, Bao., Chen, Yuan, Wang, H.F., and Xiao, T.B. (2022). Increase of defense enzyme activity and expression of resistance-related genes in pepper induced by *Bacillus* sp. Ya-1. Mol. Plant Breed. **20**:2699-2706. https://doi.org/10.13271/j.mpb.020.002699.

Zhang, J., Lv, J, Xie J. M., Gan, Y.T., Coulter, J.A., Yu, J.H., Li, J., Wang, J.W., and Zhang, X.D. (2020). Nitrogen source affects the composition of metabolites in pepper (*Capsicum annuum* L.) and regulates the synthesis of capsaicinoids through the GOGAT-GS pathway. Prog. Artif. Intell. **9**:150. https://doi.org/10.3390/foods9020150.

Zhang, Y.J., Ye, C., Su, Y.W., Peng, W.C., Lu, R., Liu, Y.X., Huang, H.C., He, X.H., Yang, M., and Zhu, S.S. (2022). Soil acidification caused by excessive application of nitrogen fertilizer aggravates soil-borne diseases: Evidence from literature review and field trials. Agr. Ecosyst. Environ. **340**:108176. https://doi.org/10.1016/j.agee.2022.108176.

Zheng, J., Yan, F., Pan, Z. P., Feng, Z.J., and Li, X.Y. (2019). Meta analysis on the effect of biogas slurry on crop yield in China. China Biogas，**37**: 78-84.
