# Supplementary material for: An altruistic rhizo-microbiome strategy in crop-rotation systems for sustainable management of soil-borne diseases
Source: Plant Commun. 2025 Sep 3;6(10):101502. doi: 10.1016/j.xplc.2025.101502 (PMC12546766; doi:10.1016/j.xplc.2025.101502)
Supplement: Document S2. Article plus supplemental information [file mmc2.pdf]

Plant Communications
Research article

CellPress
Partner Journal

An altruistic rhizo-microbiome strategy in crop-rotation systems for sustainable management of soil-borne diseases

Jiaqing Wu[1,2,3], Yixiang Liu[1,2,3], Huanjie Yu[1,2], Fuyuan Fan[1,2], Xiahong He[1,2], Youyong Zhu[1,2], Yang Dong[1,2], Min Yang[1,2,*] and Shusheng Zhu[1,2,*]

[1]State Key Laboratory for Conservation and Utilization of Bio-Resources in Yunnan, College of Plant Protection, Yunnan Agricultural University, Kunming, China

[2]Key Laboratory for Agro-Biodiversity and Pest Control of Ministry of Education, Yunnan Agricultural University, Kunming, China

[3]These authors contributed equally to this article.

***Correspondence: Min Yang (yangminscnc@126.com), Shusheng Zhu (sszhu@ynau.edu.cn)**

https://doi.org/10.1016/j.xplc.2025.101502

ABSTRACT

Crops leave a soil legacy with altruistic effects for subsequent crops but not for themselves. While research has focused on improvements in soil physicochemical properties and the suppression of non-host pathogens, the altruistic microbiome and its assembly mechanism driven by root exudates remain largely unknown. Here, we identified altruistic but self-detrimental phenomena when garlic was rotated with other crops based on meta-analysis and *in vivo* experiments. Studies utilizing a globally adopted garlic–pepper rotation system demonstrated density-dependent enrichment of key microbial taxa, especially the *Penicillium* genus, which supports the healthy growth of non-*Allium* plants but exhibits pathogenicity toward garlic. Furthermore, we found that garlic roots stably secrete diallyl disulfide (DADS) into soil, imposing reactive oxygen species (ROS) stress in the rhizosphere and reshaping the microbial community, particularly suppressing ROS-sensitive pathogens while enriching ROS-tolerant beneficial microorganisms. As a result, *Penicillium allii*, with strong oxidative stress tolerance, survives and accumulates in the highly stressful garlic rhizosphere environment, thereby playing an "altruistic but self-detrimental" role in the rotation system. In addition, preliminary field experiments showed that co-application of DADS with *P. allii* could enhance stable colonization of *P. allii*, promoting sustainable management of soil-borne diseases and improving yield. In summary, this study reveals that garlic root exudate DADS triggers ROS-mediated selection pressure, enriching stress-tolerant *P. allii* and establishing an "altruistic" microbiome succession mechanism in crop-rotation systems. This mechanism enables targeted soil-borne disease management through plant-driven microbial community engineering.

Key words: crop rotation, altruistic microbiome, diallyl disulfide, oxidative stress, soil-borne disease, *Penicillium*

Wu J., Liu Y., Yu H., Fan F., He X., Zhu Y., Dong Y., Yang M., and Zhu S. (2025). An altruistic rhizo-microbiome strategy in crop-rotation systems for sustainable management of soil-borne diseases. Plant Comm. **6**, 101502.

INTRODUCTION

Large-scale monoculture has been widely adopted because of its mechanization efficiency and short-term economic returns (Mukhovi and Jacobi, 2022). However, long-term intensive monoculture has been linked to recurrent outbreaks of soil-borne diseases and excessive fungicide application, raising concerns about food safety and ecological stability (Pérez-Brandán et al., 2014). Such disease outbreaks in monoculture systems are mainly driven by the accumulation of soil pathogens (Luo et al., 2021). Among them, soil-borne *Phytophthora* species cause annual yield losses ranging from 10% to 100% (Pokou et al., 2008; Dorrance, 2018). Historically, these pathogens have contributed to major social and ecological crises, such as the Irish famine, the decline of woody plants in the Americas, and continuing threats to global soybean and pepper production, thus undermining agricultural sustainability and farmers' livelihoods (Kamoun et al., 2015). Although resistant crop varieties and chemical fungicides are utilized for the management of soil-borne diseases, their long-term efficacy is hindered by the rapid evolution of pathogen resistance and fungicide tolerance (Xu et al., 2022; Madhushan et al., 2025). Recent evidence indicates that soil-borne diseases can be mitigated by either suppressing pathogens or enriching beneficial microbes

This is an open access article under the CC BY license (http://creativecommons.org/licenses/by/4.0/).

(Wang et al., 2021a; Luo et al., 2021), underscoring the need for eco-friendly approaches to strengthen soil microbial suppression of soil-borne pathogens.

Crop rotation is a cost-effective and widely adopted field-management practice that offers substantial advantages over monocropping. In monocropping systems, soil progressively becomes less suitable for the same crop because of the accumulation of host-specific pathogens (Ostfeld and Keesing, 2012). In contrast, crop rotation generates positive legacy effects for subsequent crops through the enrichment of beneficial microbes and disruption of host–pathogen interactions (Zhou et al., 2023). This strategy alleviates negative plant–soil feedback by fostering a healthier soil ecosystem (Wang et al., 2021a). The rhizosphere microbiome in monoculture systems often displays "self-detrimental behavior," where host-specific pathogens gradually become dominant, reducing soil adaptability to repeated planting of the same species (Zhou et al., 2023). However, through appropriate crop matching, crop rotation can suppress harmful microorganisms, sustain plant diversity, and drive dynamic microbial community shifts in agricultural ecosystems (Wang et al., 2021a; Hong et al., 2023). Despite these advantages, the mechanisms by which crops shape the assembly of specific rhizosphere microbial communities remain poorly understood.

Root exudates serve as both nutrients and signaling molecules that influence microbial growth and aggregation in the rhizosphere (Upadhyay et al., 2022). Allelopathic plants release phytoalexins, key secondary metabolites in root exudates, which recruit beneficial microbes and suppress pathogens to establish a disease-suppressive microbiome that prevents soil-borne infections (Desmedt et al., 2022). Such microbiomes can be transmitted to subsequent crops in crop-rotation systems. However, certain microorganisms (e.g., pathogens) can overcome phytoalexin-mediated defenses, colonize roots, and contribute to the soil microbial legacy (Voges et al., 2019; Wen et al., 2023). Evidence indicates that host-adapted pathogens can tolerate reactive oxygen species (ROS) bursts generated by plant defense metabolites, facilitating infection and parasitic interactions (Voges et al., 2019; Yang et al., 2022). Phytoalexins thus play a dual role in shaping microbial communities, selectively enriching or suppressing taxa depending on their ability to metabolize phytoalexins and detoxify ROS (Voges et al., 2019). Consequently, an understanding of how phytoalexins regulate rhizosphere microbiota assembly, and the related "altruistic but self-detrimental" mechanisms, is critical for efforts to improve soil health management. Thus far, the role of phytoalexin-induced ROS in microbial legacy transmission within crop-rotation systems has not been sufficiently defined.

In this study, we carried out experiments to verify the existence of microbial altruistic yet self-detrimental behavior in a widely adopted garlic–pepper rotation system and to clarify the microbial assembly mechanisms driven by garlic root exudates. Our results demonstrated that garlic roots secrete diallyl disulfide (DADS), which induces ROS stress in the rhizosphere, suppresses the ROS-sensitive pathogen *P. capsici*, and enriches ROS-tolerant *Penicillium allii*, thereby supporting the growth of non-*Allium* crops. Building on this "altruistic but self-detrimental" effect, we propose an environmentally friendly approach in which exogenous DADS is co-applied with *P. allii* to achieve sustainable management of soil-borne diseases.

RESULTS

Garlic-conditioned soil benefits the yield and disease suppression of subsequent crops

Based on a meta-analysis of 21 crop-rotation studies using garlic as the previous crop, garlic-conditioned soil significantly boosted the yield of subsequent crops (Figure 1A), particularly in Solanaceae and Cucurbitaceae species. Greenhouse experiments confirmed that soil conditioned by garlic significantly enhanced the biomass of Solanaceae crops (pepper, tobacco, and potato) but not garlic itself (Figure 1B; Supplemental Figure 1). Disease investigation experiments (Figure 1C) demonstrated that garlic-conditioned soil enhanced soil suppressiveness against *P. capsici* invasion (Figure 1D) and improved resistance in pepper stems against *P. capsici* infection (Figure 1E; Supplemental Figure 2). Soil suspensions from a Midu County garlic field (Figure 1F), a Nanhua County garlic field (Figure 1G), and potted garlic (Figure 1H) exhibited significantly higher antagonistic activity against *P. capsici* mycelial growth compared with filtered soil suspensions, indicating microbial contributions to disease suppression. Additionally, suspensions from potted garlic-conditioned soil significantly increased resistance-related metabolites in pepper shoots (Supplemental Figure 3). In contrast, pepper-conditioned soil showed no significant inhibitory effect on *P. capsici* mycelial growth (Figure 1I).

Penicillium enriched in garlic-conditioned soil suppresses pepper *Phytophthora* disease

To examine the role of garlic in the development of disease-suppressive soil, a greenhouse experiment was conducted using soils conditioned with increasing garlic planting densities (0, 1, 2, 3, and 5 garlic plants per pot, designated as CK, Z1, Z2, Z3, and Z5) (Figure 2A). Rhizosphere soils were harvested to assess their disease-suppressive effects and microbial community changes. The results showed that garlic-conditioned soils enhanced pepper resistance to *P. capsici* infection, with resistance levels 22.03%–60.90% higher than the control, showing the strongest effect at the highest planting density (Z5) (Figure 2B; Supplemental Figure 4). Soil suspensions from these soils inhibited *P. capsici* mycelial growth in a density-dependent manner (Figure 2B).

High-throughput sequencing revealed that garlic planting density significantly altered soil microbial communities. While bacterial communities showed no significant structural changes at the genus level (analysis of similarity, ANOSIM, $R = 0.0881$, $p = 0.249$) or amplicon sequence variant (ASV) level ($R = 0.1550$, $p = 0.164$), fungal communities exhibited significant beta-diversity shifts (genus level, $R = 0.3056$, $p = 0.043$; ASV level, $R = 0.3388$, $p = 0.011$) (Figure 2C; Supplemental Table 1). The Bray–Curtis distance of fungal communities increased along the PCoA1 axis with garlic density (Figure 2C), negatively correlating with pepper lesion length ($R^2 = -0.31$, $p = 0.01$) and positively correlating with mycelial inhibition ($R^2 = 0.3$, $p = 0.01$), but not with bacterial communities (lesion length, $R^2 = 0.03$, $p = 0.47$; mycelial inhibition, $R^2 = 0$, $p = 1.00$) (Figure 2D; Supplemental Figure 5). Further

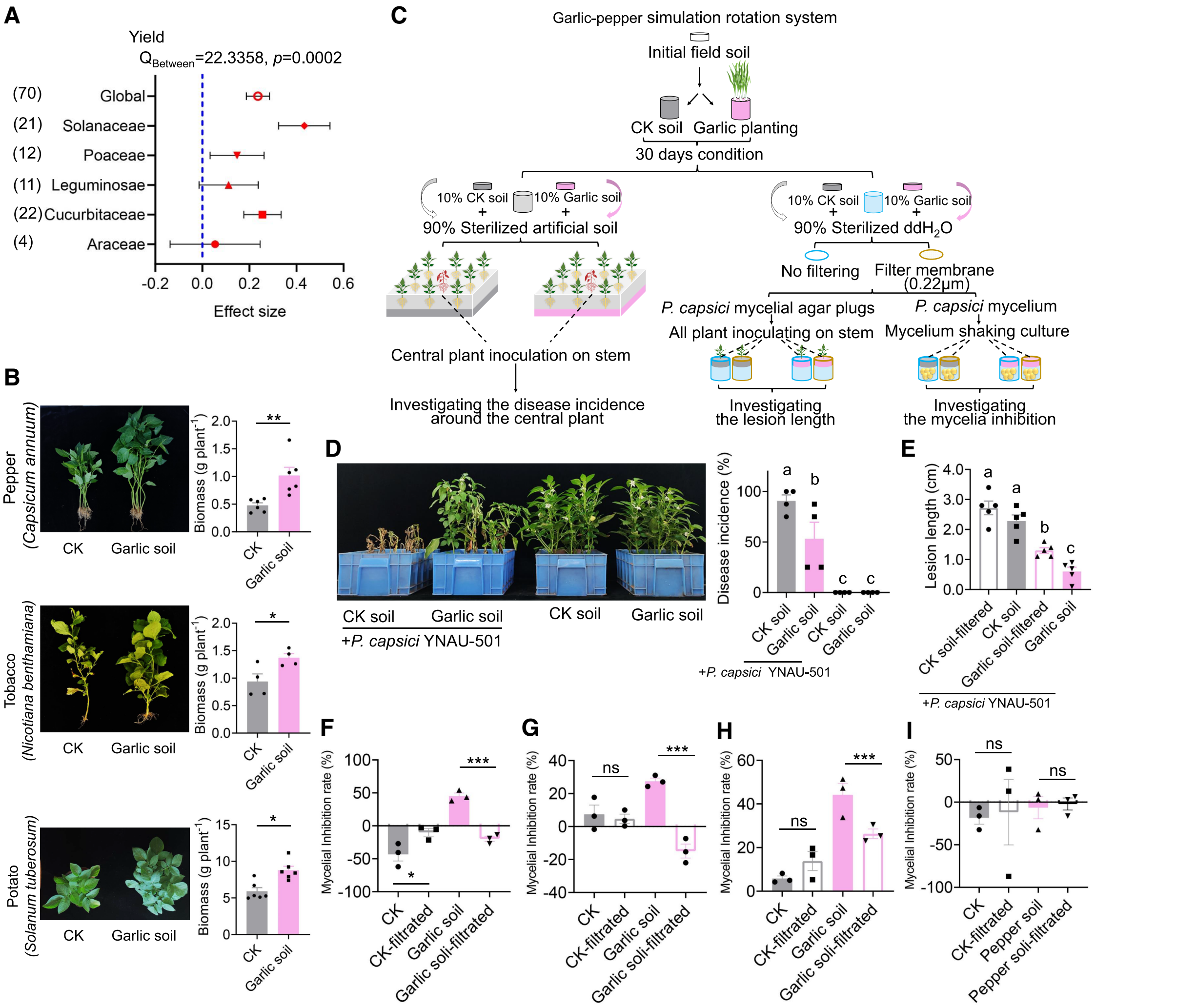

Figure 1. Altruistic effects of garlic-conditioned soil in suppressing pathogens and inducing plant resistance.

(A) Meta-analysis of the effects of garlic rotation on the yield of subsequent crops from various families. $Q_{Between}$ represents between-group heterogeneity.

(B) Effects of garlic-conditioned soil on the growth of three solanaceous crops: pepper, tobacco, and potato.

(C) Experimental design used to assess the altruistic effect of garlic-conditioned soil on *Phytophthora* blight in pepper caused by *Phytophthora capsici.*

(D) Impacts of garlic-conditioned soil on the spread of pepper *Phytophthora* blight.

(E) Effects of garlic-conditioned soil suspension, with or without microbial filtration, on the progression of *Phytophthora* blight on pepper stems.

(F) Antagonistic effect of soil suspension from field garlic-conditioned soil from Midu County, Yunnan Province, China (100.50°E, 25.30°N). **(G)** field garlic-conditioned soil from Nanhua County, Yunnan Province, China (101.27°E, 25.27°N,); and **(H)** potted garlic-conditioned soil on the mycelial growth of *P. capsici*. Solid columns indicate inhibition rates of unfiltered soil suspensions, and hollow columns indicate inhibition rates of filtered soil suspensions.

(I) Antagonistic effect of soil suspension from field pepper-conditioned soil from Xundian County, Yunnan Province, China (103.29°E, 25.51°N) on the mycelial growth of *P. capsici*. Solid columns indicate inhibition rates of unfiltered soil suspensions, and hollow columns indicate inhibition rates of filtered soil suspensions.

Data are expressed as mean ± standard error. Different lowercase letters indicate significant differences between treatments ($p < 0.05$, according to ANOVA and Duncan's multiple range test). An independent-sample *t*-test was used for significance analysis. ***$p < 0.001$, **$p < 0.01$, *$p < 0.05$, ns = not significant.

analysis of fungal taxa revealed that ASV1 (*Penicillium*, Aspergillaceae) was enriched with garlic density (Figure 2E), showing a positive correlation with planting density ($r = 0.9246$, $p < 0.001$) and mycelial inhibition ($r = 0.5979$, $p = 0.0053$), but a negative correlation with lesion length ($r = -0.6112$, $p = 0.0042$) (Supplemental Figure 6). Phylogenetically, ASV1 and ASV3062 clustered with *P. allii*, ASV396 with *P. brevicompactum*, and ASV1060 with *P. ochrochloron* (Supplemental Figure 7).

To validate the role of *Penicillium* in disease suppression, we isolated three *Penicillium* species from garlic-conditioned soil: *P. allii* (YNAU-Q-6), *P. ochrochloron* (YNAU-P-4), and *P. brevicompactum* (YNAU-Q-9). These were identified by morphology and ITS/β-tubulin amplification (Figure 2F; Supplemental Figure 8). All three isolates exhibited strong antagonistic activity against the mycelial growth of *P. capsici* (Figure 2G; Supplemental Figure 9) and significantly enhanced pepper resistance to *P.*

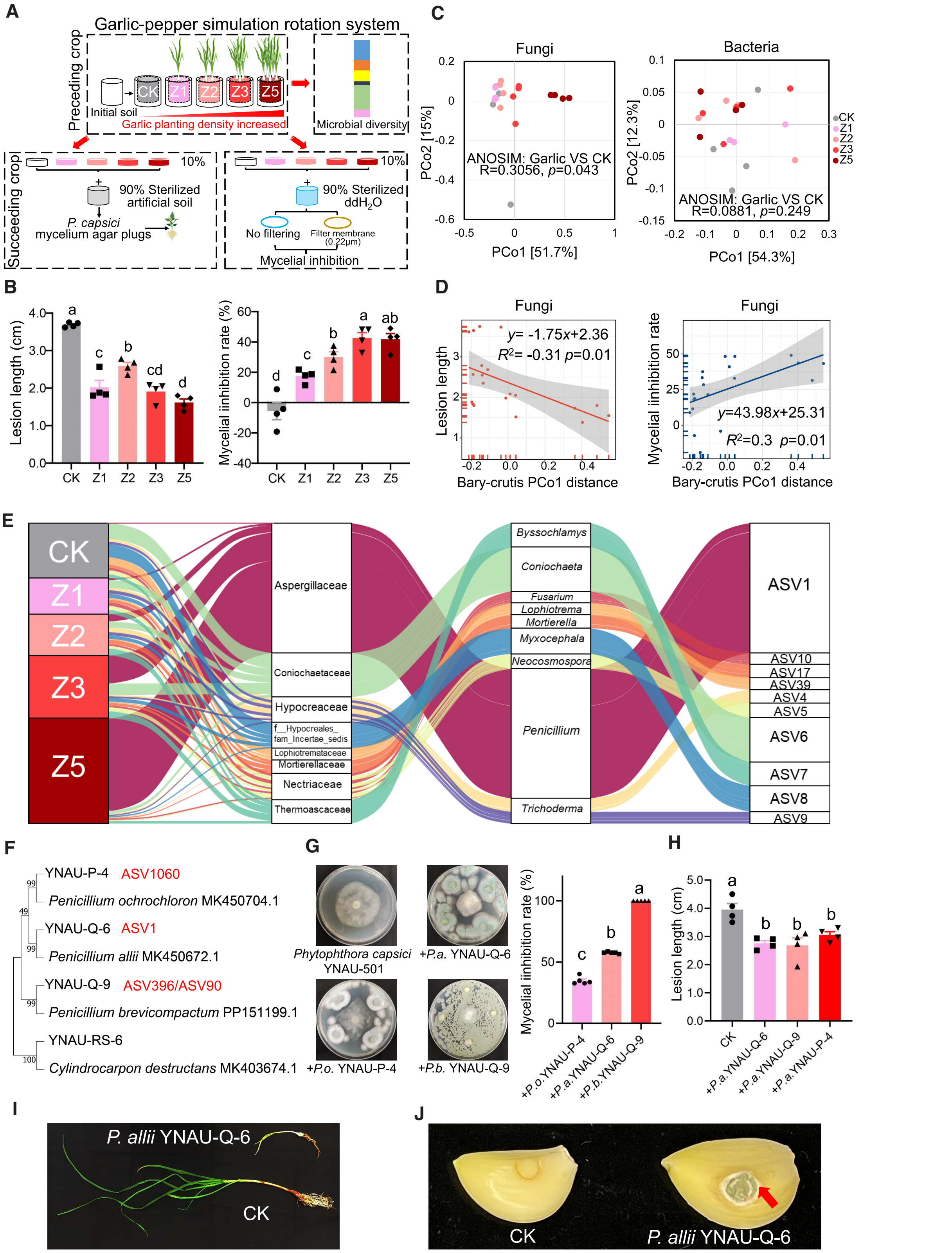

Figure 2. Influence of garlic on rhizosphere microbial community assembly and enrichment of *Penicillium* species that antagonize pathogens and induce resistance while infecting garlic itself.

(A) Experimental design for assessing the effects of garlic at different planting densities on the rhizosphere microbiome and their impact on pepper resistance to *P. capsici* and mycelial growth inhibition. Z1, Z2, Z3, and Z5 represent 1, 2, 3, and 5 garlic plants per pot, respectively.

(legend continue on next page)

capsici infection (Figure 2H; Supplemental Figure 10). *P. allii* YNAU-Q-6 specifically activated systemic immunity in pepper, as evidenced by *PR1c* upregulation and salicylic acid accumulation (Supplemental Figures 11). However, it suppressed garlic growth and caused disease symptoms in garlic without inducing *PAL* or *PR1c* expression (Figures 2I and 2J; Supplemental Figure 12). *P. ochrochloron* YNAU-P-4 and *P. brevicompactum* YNAU-Q-9 exhibited no pathogenic effects on garlic (Supplemental Figure 13).

Garlic-secreted DADS enriches altruistic *Penicillium* for pepper *Phytophthora* disease suppression

To investigate the role of root exudates in the development of disease-suppressive soil, we analyzed the chemical composition of garlic root exudates, pot-grown garlic-conditioned soil, and field-collected garlic rhizosphere soil using gas chromatography–mass spectrometry (GC–MS). DADS emerged as the most abundant and stable compound across all samples (Figure 3A; Supplemental Figures 14 and 15; Supplemental Tables 4–6). In root exudates, DADS constituted 44.39% of the total volatile organic compounds (Supplemental Table 4), a dominance mirrored in field rhizosphere soil (39.68% relative peak area; Supplemental Table 5) and pot-grown garlic-conditioned soil (56.9% relative peak area; Supplemental Table 6; Supplemental Figure 15A). Metabolomic analysis further confirmed DADS as the sole compound with a VIP value >1 (Supplemental Figure 15B). Quantitative analysis revealed that DADS concentrations in garlic root exudates increased with planting density, ranging from 8.96 to 27.39 μM (Figure 3B).

Redundancy analysis revealed that garlic planting density, DADS concentration in root exudates, and the relative abundance of *Penicillium* were significantly negatively correlated with pepper *Phytophthora* lesion length but positively correlated with *P. capsici* mycelial inhibition (Figure 3C). To test the role of DADS in microbiome assembly and *Phytophthora* disease suppression, soils were amended with DADS (0, 13.7, 27.3, 54.7, and 109.4 μmol kg^{-1}; designated as CK, D1, D2, D3, and D4) and conditioned for 1 month. All DADS-treated soils significantly reduced pepper blight lesions (Figure 3D; Supplemental Figure 11) and inhibited *P. capsici* mycelial growth, with the strongest suppression observed in the D1 treatment (Figure 3D). ITS sequencing revealed significant fungal community shifts in DADS-treated soils (ANOSIM, $p < 0.05$; Supplemental Table 2), with *Penicillium* enriched at D1–D2 concentrations (Figure 3E). DADS (13.7 μM, corresponding to the D1 concentration) promoted the growth of culturable *Penicillium* strains YNAU-P-4 and YNAU-Q-6 (Figure 3F) but inhibited soil-borne pathogens (*Cylindrocarpon destructans* YNAU-RS-6 and *P. capsici* YNAU-501) in a dose-dependent manner (Figures 3G and 3H). Collectively, these results demonstrate that garlic-derived DADS drives fungal community assembly, enriches *Penicillium*, and suppresses pepper *Phytophthora* disease.

ROS tolerance mediates *P. allii* resistance to garlic-derived DADS

Gene expression profiles of *P. allii* YNAU-Q-6 (Figure 4A; Supplemental Figure 17A and 17B) compared with the pathogens *C. destructans* YNAU-RS-6 (Figure 4B; Supplemental Figure 17C) and *P. capsici* YNAU-501 (Figure 4C; Supplemental Figure 17D) following DADS exposure revealed distinct responses. Kyoto Encyclopedia of Genes and Genomes (KEGG) pathway analysis showed significant enrichment of glutathione metabolism and peroxisome-related pathways (Figure 4A–4C; Supplemental Figures 18–21). Gene Ontology (GO) analysis further indicated that *C. destructans* and *P. capsici* underwent oxidative stress responses, including cellular detoxification and redox homeostasis (Figure 4B and 4C; Supplemental Figures 24 and 25), whereas *P. allii* showed no such enrichment (Figure 4A; Supplemental Figures 22 and 23).

In *P. allii* YNAU-Q-6, only a limited number of genes were regulated by DADS, with *GST* and *FSP1* genes showing significant upregulation indicative of ROS induction, while strong upregulation of *CYP628* mitigated oxidative stress effects, allowing *P. allii* to maintain growth (Figure 4D; Supplemental Figure 17A and 17B). Conversely, both pathogens exhibited extensive oxidative stress pathway signatures. *C. destructans* upregulated peroxidase and antioxidant genes but downregulated hydrogen peroxide catabolism genes (Figure 4B; Supplemental Figure 24), while *P. capsici* showed superoxide dismutase (*SOD*) upregulation but downregulation of multiple ROS-related processes (Figure 4C; Supplemental Figure 25).

Both pathogens significantly overexpressed antioxidant genes (*GST* and *SOD*; Figure 4E and 4F) yet downregulated critical detoxification genes (*CYP53A1* in *C. destructans*; *bglX/ABC1* in *P. capsici*) and growth-related genes (*CHS1/ECHS1* in *C. destructans*; *CDC6* in *P. capsici*), leading to growth inhibition (Figure 4E and 4F). Experimental validation confirmed these findings. H_2O_2 susceptibility tests demonstrated dose-dependent inhibition of the pathogens, while *P. allii* was unaffected (Figure 4D–4F). Quantitative reverse-transcription PCR (RT–qPCR) results strongly corroborated the RNA sequencing (RNA-seq) data (Supplemental Figure 26), and 2′,7′-dichlorodihydrofluorescein diacetate (DCFH-DA) staining combined with quantitative ROS detection confirmed elevated ROS in pathogens but not in *P. allii* (Figure 4G and 4H; Supplemental Figure 27). These findings demonstrate that differences in

(B) Effects of varying garlic planting densities on the ability of rhizosphere microorganisms to inhibit disease lesion expansion and mycelial growth.
(C) Beta-diversity analysis of fungi and bacteria in the garlic rhizosphere at the genus level.
(D) Correlation analysis between fungal community structure (PCo1 axis) at the genus level and lesion length or mycelial inhibition.
(E) Relative proportions of the top 10 fungal taxa from the family level to the ASV level with increasing garlic densities. ASV = amplicon sequence variant.
(F) Phylogenetic tree of four isolated *Penicillium* species from rhizosphere soil at the ASV level based on ITS sequencing. Bootstrap values based on 1000 replications are shown as percentages at each branch.
(G) Antagonistic effects of isolated *Penicillium* against *P. capsici.*
(H) Induced resistance of isolated *Penicillium* in *P. capsici*-infected pepper plants.
(I and J) Symptoms of *P. allii* inoculation on garlic seedlings and cloves. Data are expressed as mean ± standard error. Different lowercase letters indicate significant differences between treatments ($p < 0.05$, according to ANOVA followed by Duncan's multiple range test).

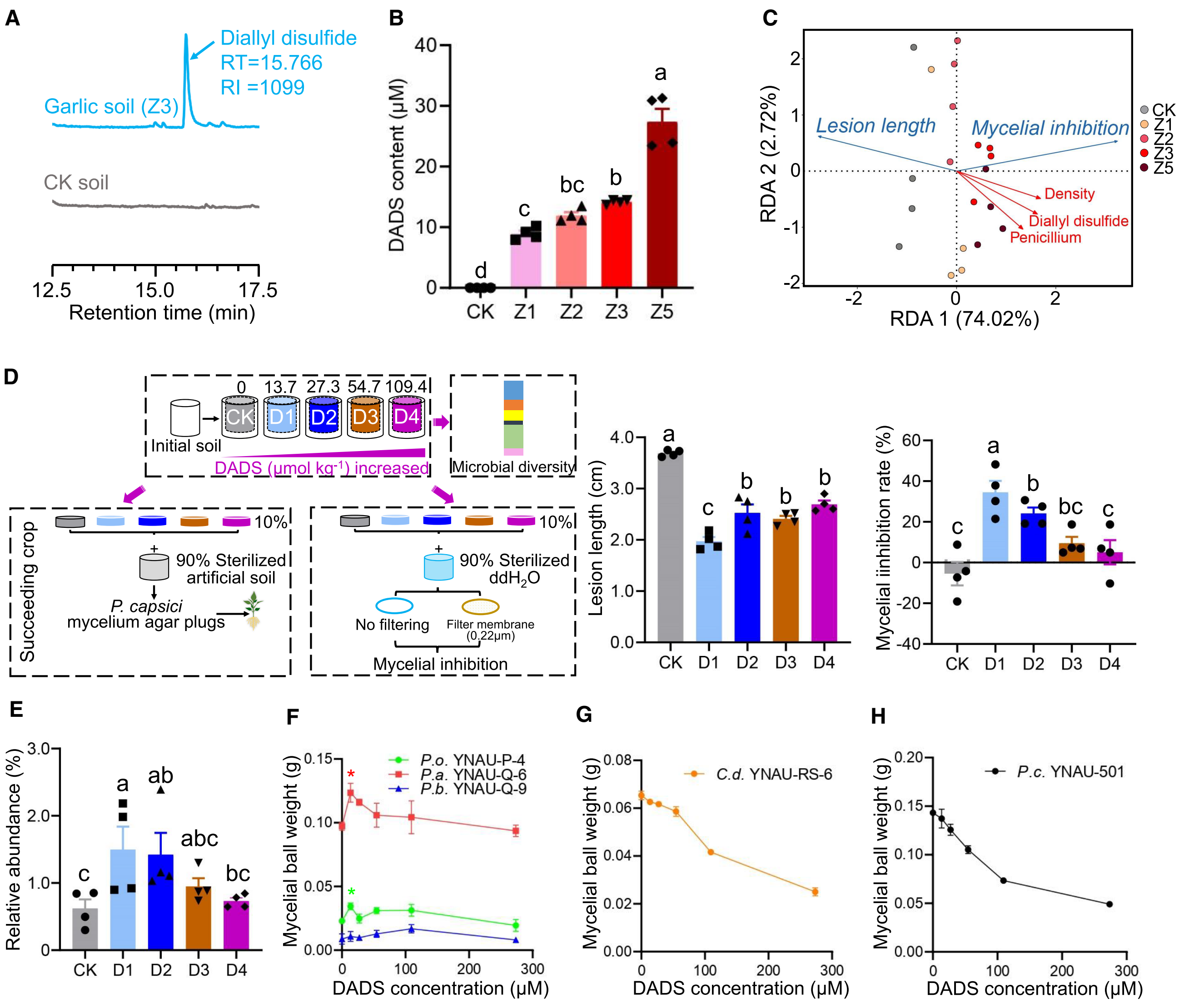

Figure 3. Garlic root-secreted sulfides modify the soil microbiome and enrich *Penicillium* species to suppress *Phytophthora* disease in pepper.

(A) Chromatogram of diallyl disulfide (DADS) in garlic-conditioned rhizosphere soil compared with control (CK) soil.

(B) Quantitative analysis of DADS in garlic root exudates at varying planting densities.

(C) Redundancy analysis (RDA) of lesion length or mycelial inhibition in relation to garlic planting density, DADS concentration, and *Penicillium* abundance.

(D) Experimental setup illustrating how different concentrations of DADS shape the soil microbiome and affect pepper resistance to *P. capsici* and mycelial growth inhibition. D1, D2, D3, and D4 represent soil DADS concentrations of 13.7, 27.3, 54.7, and 109.4 μmol kg^{-1}, respectively.

(E) Relative abundance of *Penicillium* in soil enriched by varying concentrations of DADS.

(F) Effects of different DADS concentrations on the mycelial growth of three isolated *Penicillium* strains.

(G and H) Effects of different DADS concentrations on the mycelial growth of soil-borne pathogens *C. destructans* and *P. capsici.*

Data are expressed as mean ± standard error. Different lowercase letters indicate significant differences between treatments ($p < 0.05$, according to ANOVA with Duncan's multiple range test). An independent-sample *t*-test was used for significance analysis. *$p < 0.05$.

microbial sensitivity to DADS-induced ROS bursts underlie the observed growth inhibition phenotypes, with *P. allii* exhibiting greater oxidative stress tolerance than the pathogenic fungi.

DADS-induced oxidative stress drives soil community reassembly and *Penicillium* enrichment

To verify that DADS-induced oxidative stress mediates rhizosphere microbiome reassembly, we simulated oxidative stress by exogenously applying H_2O_2 at varying concentrations and analyzed subsequent changes in soil microbial communities and disease suppression capacity (0, 85.9, 171.7, 343.4, and 686.9 μmol kg^{-1}; designated as CK, H1, H2, H3, and H4) (Figure 5A). The results showed that all H_2O_2-treated soils significantly reduced pepper blight lesion length, with H3 showing the strongest suppression (Figure 5B; Supplemental Figure 28). Mycelial inhibition assays confirmed dose-dependent suppression of *P. capsici* by H1–H3 treatments (Figure 5B). Similar to

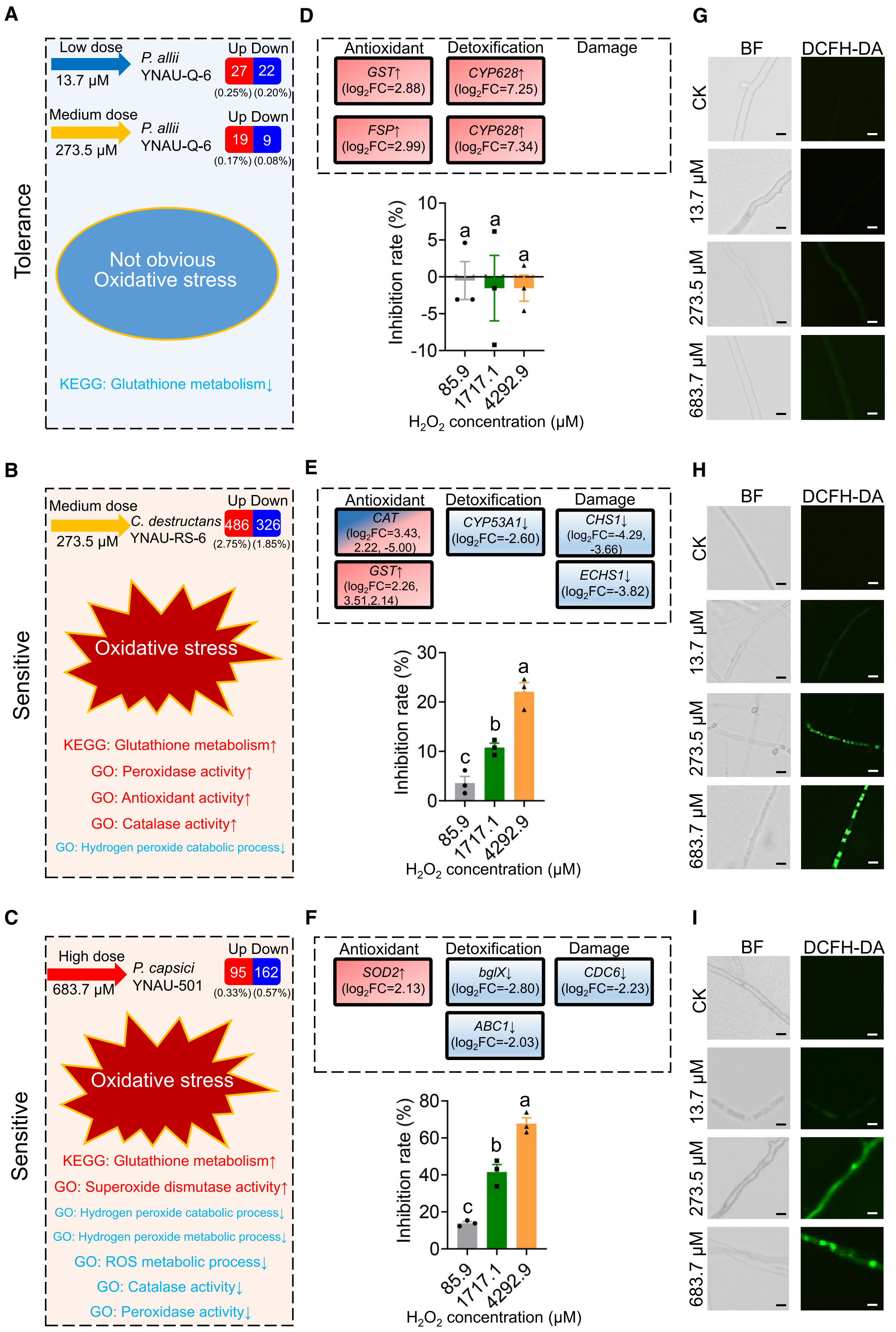

Figure 4. Differential tolerance to DADS-induced ROS among the garlic pathogen *P. allii* YNAU-Q-6 and the non-garlic pathogens *C. destructans* YNAU-RS-6 and *P. capsici* YNAU-501.

(A–C) Transcriptomic changes in *P. allii*, *C. destructans*, and *P. capsici* following exposure to DADS. Values in brackets represent the proportion of differentially expressed genes. GO = Gene Ontology. KEGG = Kyoto Encyclopedia of Genes and Genomes. Up arrow indicates pathways enriched by upregulated differentially expressed genes, and down arrow indicates pathways enriched by downregulated genes.

(legend continue on next page)

DADS treatments, H_2O_2 application significantly altered fungal community structure compared with controls at both genus and ASV levels (ANOSIM, $p < 0.05$, Supplemental Table 3). Consistent with the garlic- and DADS-conditioned microbiome, *Penicillium* was significantly enriched by H_2O_2 treatment, with H1, H2, and H3 treatments driving significant enrichment relative to CK (Figure 5C). ANOSIM confirmed high similarity between H_2O_2- and DADS-driven communities (Supplemental Figure 29). Metagenomic analysis revealed downregulation of thiamine metabolism in Z3 and H3 treatments, while pathways such as RNA transport were significantly upregulated (Figure 5D and 5F; Supplemental Figure 30). Biochemical assays validated inhibited thiamine utilization in Z3, D3, and H3 treatments ($p < 0.05$; Supplemental Figure 31). Additionally, D3 and Z3 treatments significantly increased apoptosis-related pathways (e.g., apoptosis-fly) (Figure 5D and 5E). These results demonstrate that garlic-derived DADS reshapes rhizosphere microbiomes via ROS stress, favoring *Penicillium* enrichment and functional adaptation.

Co-application of DADS and *Penicillium* for disease suppression and yield improvement

We conducted field trials to verify the effects of co-applying *Penicillium* and DADS on disease suppression and yield improvement across multiple crops. Three *Penicillium* isolates demonstrated significant *in vitro* antagonistic activity against four economically important *Phytophthora* pathogens: *P. parasitica* var. *nicotianae* (tobacco), *P. cactorum* (strawberry), *P. infestans* (potato), and *P. sojae* (soybean) (Supplemental Figure 32). Field trials showed that combining *Penicillium* isolates with DADS improved disease control and yield in pepper, tobacco, and soybean. In pepper, co-application of 273.5 μM DADS and *P. allii* YNAU-Q-6 significantly reduced *Phytophthora* blight incidence (Figure 6A) and increased yield (Figure 6B). Similar benefits were observed in tobacco and soybean (Supplemental Figure 33). Pot experiments further revealed that two applications of *Penicillium* with DADS maximized pepper biomass (Figure 6C) and disease resistance (Figure 6D). Comparable effects were also observed in tobacco and soybean (Supplemental Figure 33). Absolute quantitative analysis indicated that colonization of *P. allii* YNAU-Q-6 in the pepper rhizosphere was enhanced by DADS (Figure 6E). These findings indicate that DADS primes soil microbiomes to enrich beneficial *Penicillium* strains, providing cross-crop protection and yield gains (Figure 6F).

DISCUSSION

Crop rotation is widely adopted as a sustainable strategy for the management of soil-borne diseases. Although it is well documented that crop rotation enhances soil physical and chemical properties and disrupts host–pathogen interactions (Wang et al., 2021b; He et al., 2024), emerging evidence has revealed a complex phenomenon: crops leave behind a soil legacy that provides altruistic benefits for subsequent crops but imposes detrimental effects on themselves. An understanding of this altruistic yet self-detrimental behavior is essential for efforts to refine crop-rotation systems. In the present study, we demonstrate that garlic mediates such legacy effects through rhizosphere microbiome reprogramming. Garlic root exudates, particularly DADS, generate ROS stress that restructures microbial communities. This stress selectively suppresses ROS-sensitive non-host pathogens while enriching ROS-tolerant beneficial microbes and garlic-specific pathogens, especially *P. allii*. Consequently, the garlic-conditioned rhizosphere microbiome supports the health of subsequent crops but creates a self-detrimental environment for garlic itself. Our findings support a practical approach: co-application of DADS and *P. allii* to harness garlic's altruistic legacy for sustainable disease suppression and yield improvement. This strategy aligns with principles of eco-friendly agriculture by exploiting natural plant–microbe interactions.

The garlic-conditioned rhizosphere microbiome exhibits altruistic effects on non-*Allium* crops while remaining detrimental to garlic. Recognition of this duality is critical when optimizing crop-rotation practices to maximize both disease control and productivity. *Allium* crops, including garlic, are established tools for soil-borne disease management (Ding et al., 2018). Our meta-analysis and *in vivo* experiments confirmed that garlic-conditioned soil microbiomes enhance the growth of solanaceous crops (e.g., tobacco, pepper) by suppressing pathogens and inducing host resistance. These beneficial outcomes were associated with enrichment of diverse taxa, including *Ramlibacter*, *Pseudomonas*, *Bacillus*, *Enterobacter*, *Escherichia–Shigella*, *Pantoea*, *Enterococcus*, *Lysinibacillus*, *Paenibacillus*, *Kluyvera*, and *Jeotgalibacillus* (Hong et al., 2023; Zhou et al., 2023; He et al., 2024). *P. allii* exemplifies the dual role of the microbiome. As plant growth-promoting fungi, *Penicillium* spp. inhibit pathogens via production of antimicrobial metabolites and induction of resistance (Duan et al., 2014; Zhuang et al., 2021; Liu et al., 2023). However, *P. allii* also exerts self-detrimental effects, becoming pathogenic to garlic under stress conditions such as high planting density or during post-harvest storage. This phenomenon mirrors patterns observed in other systems, including *Burkholderia cepacia* in onion and *Streptomyces acidiscabies* in potato (Shang et al., 2010; Chen et al., 2023). Such findings are consistent with host selection theory, which proposes that plants tend to recruit microorganisms offering immediate benefits but may inadvertently favor pathogens over time (Xiong et al., 2021; Yang et al., 2025). To mitigate these risks while preserving beneficial traits, biotechnological approaches could be adapted for *Penicillium* strains. For instance, pathogenic gene deletion (as demonstrated in engineered *Burkholderia ambifaria*; Mullins et al., 2019) or the horizontal transfer of detoxification genes (e.g., *CYP* genes; Guo et al., 2024) may improve microbial safety without diminishing biocontrol efficacy. Harnessing altruistic microorganisms while minimizing their self-detrimental traits is essential for sustainable agriculture.

(D–F) Changes in antioxidant, detoxification, and damage-related gene expression in different microorganisms, along with inhibition rates of H_2O_2 on *P. allii*, *C. destructans*, and *P. capsici.* Up arrow indicates upregulated genes, and down arrow indicates downregulated genes.

(G–I) DCFH-DA staining for ROS detection in *P. allii*, *C. destructans*, and *P. capsici* after DADS treatment. BF = bright field. ,Scale bar 10 μm.

Data are expressed as mean ± standard error. Different lowercase letters indicate significant differences between treatments ($p < 0.05$, according to ANOVA with Duncan's multiple range test).

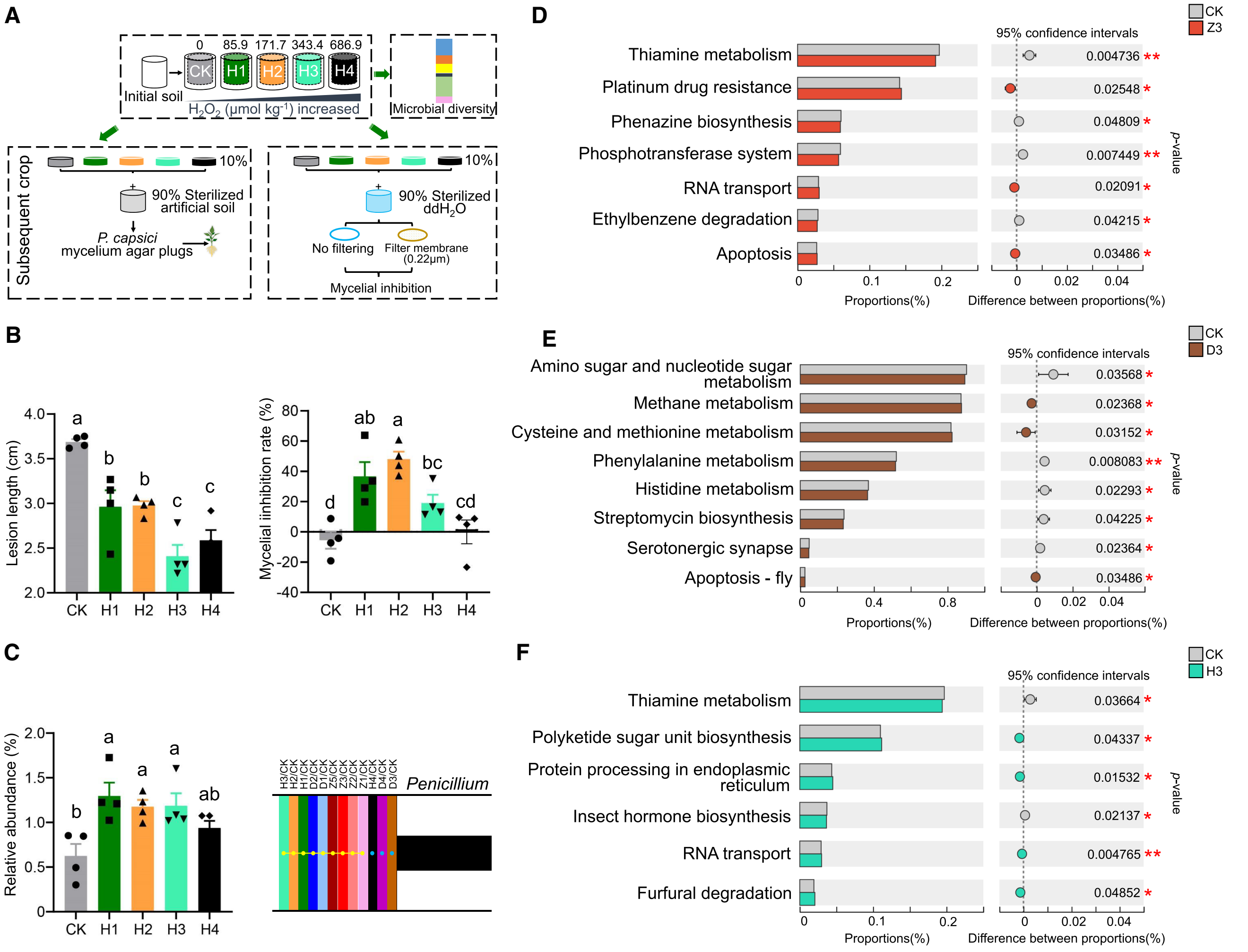

Figure 5. Oxidative stress drives the differential selection of soil microorganisms, leading to the formation of disease-suppressive soil.

(A) Experimental setup illustrating the effect of different H_2O_2 concentrations on the soil microbiome and their impact on pepper resistance to *P. capsici* and mycelial growth inhibition. H1, H2, H3, and H4 represent soil H_2O_2 concentrations of 85.9, 171.7, 343.4, and 686.9 μmol kg^{-1}, respectively.

(B) Outcomes of changes in the soil microbiome under varying H_2O_2 concentrations on pepper resistance and mycelial growth.

(C) Relative abundance of *Penicillium* in soil enriched with different H_2O_2 concentrations, along with an upset diagram showing microbial co-enrichment under garlic planting, DADS treatment, and H_2O_2 treatment.

(D–F) Significant changes in the soil microbiome at KEGG level 3 pathways after planting three garlic plants (Z3), DADS treatment at 54.7 μmol kg^{-1} soil (D3), and H_2O_2 treatment at 343.4 μmol kg^{-1} (H3).

Data are expressed as mean ± standard error. Different lowercase letters indicate significant differences between treatments ($p < 0.05$, according to ANOVA followed by Duncan's multiple range test). An independent-sample *t*-test was used for significance analysis. *$p < 0.05$, **$p < 0.01$, ***$p < 0.001$.

Future research should prioritize two directions: systematic screening of rotation crops to maximize microbiome-mediated benefits, and targeted engineering of microbial strains to separate their beneficial and pathogenic characteristics.

Root-secreted antimicrobial metabolites shape rhizosphere microbiomes through ROS-mediated selection. Garlic roots release DADS, a stable sulfide compound that imposes ROS stress and restructures rhizosphere microbial communities. This study demonstrates how DADS drives the formation of altruistic yet self-detrimental microbial assemblages in garlic rhizosphere soil. At physiological concentrations, DADS exhibits selective antimicrobial activity: suppressing non-host pathogens such as *C. destructans* and *P. capsici* while favoring tolerant microbes such as *Penicillium* spp. Antimicrobial assays revealed the superior tolerance of *Penicillium* to DADS-induced ROS stress compared with sensitive pathogens. Transcriptomic analysis (RNA-seq) and ROS staining demonstrated that *Penicillium* activates strong antioxidant defenses, including upregulation of glutathione S-transferase (*GST*) and cytochrome P450 (*CYP628*) genes. In contrast, susceptible pathogens showed downregulation of key genes (*ECHS1*, *CDC6*), impairing DNA replication and cell division (Wu et al., 2024). Therefore, although DADS-mediated ROS stress assists garlic in defending against non-host pathogens, it also enriches *Penicillium*, a microorganism that can function both as a beneficial microbe and as a pathogen. This dual role underscores the trade-off inherent in garlic's defense strategy, where long-term co-evolution has allowed certain

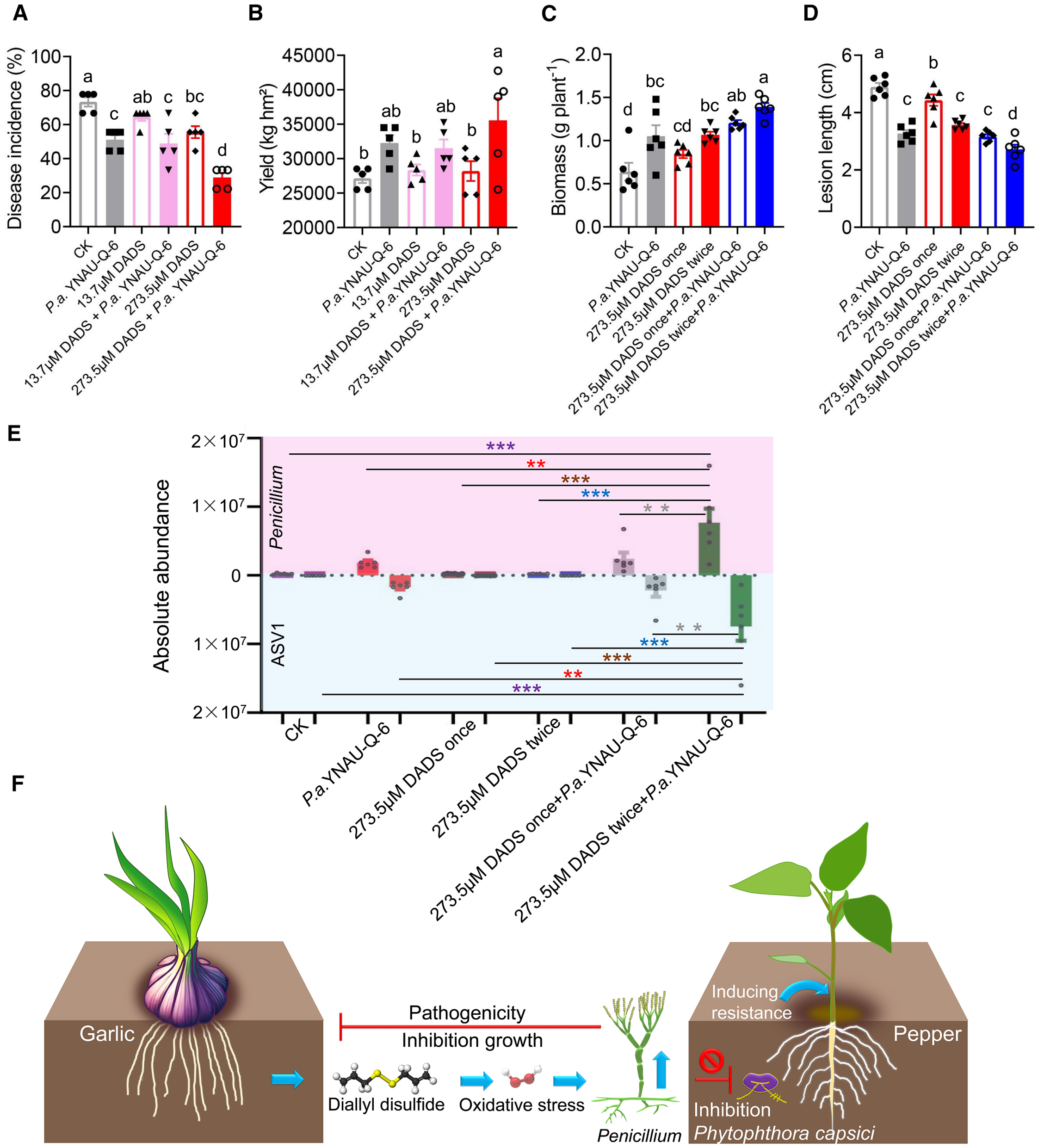

Figure 6. Co-application of DADS and *Penicillium* in the suppression of *Phytophthora* disease and improvement of yield.
(A and B) Effects of the co-application of *P. allii* YNAU-Q-6 and DADS on pepper *Phytophthora* disease and yield in the field.
(C and D) Effects of the co-application of *P. allii* YNAU-Q-6 and DADS on pepper yield and *Phytophthora* disease in pot experiments.
(E) Effects of the co-application of DADS on the colonization of *P. allii* YNAU-Q-6 in pepper rhizosphere soil. ASV = amplicon sequence variant.
(F) Altruistic behavior and mechanisms of garlic rhizosphere microorganisms in a crop-rotation system: DADS-induced soil microbial oxidative stress drives the formation of disease-suppressive soil.
Data are expressed as mean ± standard error. Different lowercase letters indicate significant differences between treatments ($p < 0.05$, according to ANOVA followed by Duncan's multiple range test). An independent-sample *t*-test was used for significance analysis. $^{*}p < 0.05$, $^{**}p < 0.01$, $^{***}p < 0.001$.

pathogens to circumvent chemical defenses (Cavagnaro et al., 2005). Metagenomic analysis revealed that garlic cultivation induces oxidative stress in the rhizosphere, enriching stress-response pathways and apoptosis-related mechanisms. Experimental validation demonstrated that exogenous DADS or H_2O_2 application can reproduce these community shifts, confirming ROS as the main driver (Wu et al., 2024). These findings extend beyond garlic. Many plant species utilize similar chemical defenses, releasing secondary metabolites such as phenolic acids, allyl isothiocyanate, and benzoxazines that trigger pathogen ROS bursts (Voges et al., 2019; Liu et al., 2021). Such convergent stress responses may drive microbial communities toward functional homogeneity (Ning et al., 2024), suggesting a broader paradigm in plant–microbe interactions. By exploiting root exudate–ROS–microbe dynamics, it may be possible to design optimized rotation sequences that maximize beneficial microbial legacy effects, develop targeted biocontrol strategies using ROS-tolerant beneficial microbes, and implement chemical

priming approaches to precondition soils for subsequent crops. This work links chemical ecology with applied agriculture, offering solutions to the inherent trade-offs in plant defense strategies.

Effective soil-borne disease management requires stable colonization of beneficial microorganisms, which depends on persistent ecological drivers. In agricultural practice, bioinoculants often fail to deliver consistent field performance despite promising laboratory results, largely because of disparities between controlled experimental conditions and the complexity of rhizosphere environments (Sánchez-Gil et al., 2023). Factors such as soil heterogeneity, host genotype variation, and environmental fluctuations frequently restrict the persistence and functionality of introduced strains (Liu et al., 2024). The present study demonstrates that co-application of DADS as a prebiotic and *P. allii* as a probiotic addresses these limitations through synergistic mechanisms. In contrast to monofunctional bioinoculants, this dual strategy harnesses ecological selection pressures to enhance microbial sustainability. DADS, a sulfur-containing secondary metabolite released from garlic roots, establishes oxidative stress niches that selectively enrich stress-adapted *P. allii* while suppressing pathogens. This mutualistic relationship reflects natural rhizosphere selection processes, enabling *P. allii* to become a dominant community member. The prebiotic–probiotic synergy provides several distinct advantages. First, DADS sustains *P. allii* colonization by continually modifying the soil microenvironment, analogous to the manner in which inositol supports *Pseudomonas fluorescens* persistence through siderophore induction (Sánchez-Gil et al., 2023). Second, DADS exhibits multifunctional activity—directly suppressing pathogens at bioactive concentrations (Cheng et al., 2016) while promoting plant growth. Third, emerging evidence suggests that DADS indirectly recruits beneficial microbes by modulating root exudate profiles (Zhou et al., 2024), although such mechanisms warrant further investigation.

In summary, this study identified *Penicillium* as the principal microbial component underlying garlic's altruistic effects, revealing remarkable tolerance to garlic-derived DADS-induced oxidative stress. We elucidated the chemical–ecological mechanisms shaping these altruistic interactions in crop-rotation systems, leading to practical insights for improved disease management. The co-application of DADS and *Penicillium* provides an environmentally friendly strategy for sustainable control of soil-borne pathogens, combining plant-derived metabolites with stress-adapted beneficial microbes to enhance field performance.

METHODS

Meta-analysis of garlic rotation effects on subsequent crop yield and verification in pot experiments

This meta-analysis integrated data from 21 independent studies (70 observational data points) evaluating the effects of garlic rotation on subsequent crop yields (Supplemental Table 7). The treatment group comprised fields cultivated with garlic; control groups included fallow plots, winter wheat fields aligned with garlic planting schedules, and continuous cropping systems. Yield data—including means, standard deviations, and replication numbers—were extracted in accordance with established protocols (Zhang et al., 2022). Random-effects models were applied using MetaWin 3.0.14 to calculate global effect sizes with 95% confidence intervals (CIs). Statistical significance was inferred from the absence of overlap between 95% CIs and zero. Complete methodological details are provided in the Supplemental Methods.

Soil-conditioning effects were assessed using a standardized bioassay. Garlic-conditioned soil collected via the root bag method (see Supplemental Methods) or unplanted control soil was mixed with sterilized substrate (moist-heat sterilization at 121°C for 30 min) at a 1:9 weight ratio (Ma et al., 2020). Three solanaceous species were tested: pepper (*Capsicum annuum*), tobacco (*Nicotiana benthamiana*), and potato (*Solanum tuberosum*). Total plant dry biomass at 60 days after planting was considered the primary endpoint. Each treatment included a minimum of four replicates to ensure statistical robustness.

Assessment of *Phytophthora* disease suppression by garlic-conditioned soil

The capacity of garlic-conditioned soil to suppress *Phytophthora* disease was evaluated through a standardized bioassay (Figure 1C). Garlic-conditioned or unplanted control soil was mixed with sterilized soil (moist-heat sterilization at 121°C for 30 min) at a 1:9 (w/w) ratio. Healthy pepper seedlings were transplanted into pots (34.5 × 26.5 × 12.5 cm) containing the soil mixtures, with nine plants per pot. After 70 days of growth, the central seedling was inoculated with *P. capsici*; surrounding seedlings served as indicators of disease spread. Disease incidence was recorded 60 days after inoculation, and blank PDA plates were used as negative controls. Each treatment consisted of four biological replicates (Yang et al., 2014).

The capacity of garlic-conditioned soil microorganisms to induce resistance was examined through hydroponic and pot experiments (Figure 1C). For hydroponic testing, pepper seedlings were maintained in 220-ml tissue culture bottles containing 60 ml of sterilized Hoagland solution. Soil extracts were prepared by mixing 100 g of garlic-conditioned or control soil with 900 ml of sterile ddH_2O (1:9 w/v), followed by 30 min of rotation (140 rpm, 25°C) and filtration. Two types of filtrates were obtained: 0.22-μm membrane-filtered (microbe-free) and unfiltered. Seedlings received 20 ml of the respective extracts for 5 days before inoculation with *P. capsici.* Stem lesion lengths were measured 5 days post-inoculation, with four replicates per treatment (one plant per bottle) (Gu et al., 2020). Pot experiments used containers (10.5 cm upper diameter × 9.5 cm height) filled with soil mixtures (1:9 treated:sterile soil, w/w). Seventy-day-old pepper seedlings were transplanted and pre-treated for 5 days, then inoculated with *P. capsici*. Lesion development was analyzed in relation to soil microbial community composition (Figure 2A). Each pot contained six seedlings, with four replicate pots per treatment.

The antifungal activity of soil microorganisms was quantified through liquid culture inhibition assays (Figure 1C; Figure 2A). Soil suspensions (3 g soil in 27 ml sterile water) were prepared as described above. Inhibition of *P. capsici* was assessed by adding 5 ml of soil suspension to 45 ml of carrot liquid medium containing five pre-cultured mycelial plugs (5 mm diameter). Cultures were incubated at 140 rpm for 72 h, then filtered and dried at 60°C for biomass quantification. Controls consisted of sterile water in place of soil suspension, with three replicates per treatment (Luo et al., 2021; Li et al., 2023).

Qualitative and quantitative detection of garlic rhizosphere sulfides

Volatile sulfur compounds in garlic rhizosphere soil were qualitatively assessed using headspace solid-phase microextraction coupled with GC–MS (HS–SPME/GC–MS). Five grams of garlic-conditioned soil (Z3, three garlic plants per pot) or control soil (CK) were placed in sealed collection bottles. A 50/30 μm DVB/CAR/PDMS SPME fiber (57328-U, Supelco) was exposed to the headspace at 35°C for 1 h prior to thermal desorption in the GC–MS inlet. Metabolite profiling was performed using MicrobiomeAnalyst (https://www.microbiomeanalyst.ca/). Complete

GC–MS parameters are provided in the Supplemental Methods (Qian et al., 2022).

DADS and related sulfur metabolites were quantified in garlic root exudates through liquid–liquid extraction followed by GC–MS. Garlic seedlings were grown in root bags at densities of 1–5 plants per pot for 30 days, then transferred to hydroponic systems containing 125 ml of sterile ddH_2O for 48 h of exudate collection (Luo et al., 2022). The experiment included four biological replicates, each consisting of pooled exudates from six pots (24 pots per treatment). For sample preparation, 10-ml aliquots of exudate were mixed with 20 ml of ethyl acetate (1:2 v/v), concentrated by rotary evaporation at 40°C, and reconstituted in 6 ml of n-hexane. After filtration through 0.22-μm polytetrafluoroethylene membranes, samples were stored in amber vials at 4°C for less than 24 h before analysis. A five-point calibration curve (0, 34.2, 68.4, 102.6, and 136.7 μM) was generated via serial dilution of authentic sulfur standards in n-hexane (Ji et al., 2025). GC–MS analysis utilized parameters identical to the HS–SPME method, enabling direct comparison between volatile and soluble sulfur pools. This dual approach allowed comprehensive characterization of both volatile and water-soluble sulfur compounds in the garlic rhizosphere.

High-throughput sequencing and metagenomic analysis of microbial communities

High-throughput sequencing. Soil DNA was extracted using the E.Z.N.A. Soil DNA Kit (Omega Bio-tek, Norcross, GA, USA). Specific sequences were amplified using internal transcribed spacer (ITS) primer pairs ITS1F (5′-CTTGGTCATTTAGAGGAAGTAA-3′) and ITS2R (5′-GCTGCGTTCTT CATCGATGC-3′) for fungal communities, and 16S rRNA primer pairs 338F (5′-ACTCCTACGGGAGGCAGCAG-3′) and 806R (5′-GGACTACHVG GGTWTCTAAT-3′) for bacterial communities. PCR amplification was performed under the following conditions: initial denaturation at 95°C for 3 min, followed by 27 cycles of denaturation (95°C, 30 s), annealing (55°C, 30 s), and elongation (72°C, 30 s), with a final extension at 72°C for 10 min. PCR products were separated via electrophoresis on 1% agarose gel, then pooled in equimolar concentrations based on Qubit quantification. Paired-end sequencing was performed on the Illumina MiSeq platform at Majorbio Bio-Pharm Technology (Shanghai, China) following manufacturer protocols. Each dataset was independently re-processed using a standardized bioinformatics pipeline with QIIME2 and DADA2. Taxonomic classification of bacterial sequences was performed with the SILVA 16S rRNA gene database using the Naive Bayes classifier. Fungal taxonomic assignments were conducted with the UNITE database (version 8.3). ASVs were taxonomically annotated with a scikit-learn multinomial Naive Bayes classifier implemented in QIIME2 to represent sequence and abundance information (Simonin et al., 2022).

Metagenomic analysis. Total genomic DNA was extracted using the Mag-Bind Soil DNA Kit (Omega Bio-tek) according to the manufacturer's instructions. DNA concentration and purity were determined with a TBS-380 fluorometer and a NanoDrop2000 spectrophotometer, respectively. DNA quality was verified by electrophoresis on 1% agarose gel. Data were analyzed on the Majorbio Cloud Platform (http://www.majorbio.com). Briefly, paired-end Illumina reads were trimmed of adapters, and low-quality reads (length < 50 bp, quality score < 20) were removed using fastp (v0.20.0, https://github.com/OpenGene/fastp). Contigs ≥300 bp in length were retained for assembly and used for subsequent gene prediction and annotation. KEGG annotation was performed using DIAMOND (v0.8.35) against the KEGG database (Yin et al., 2025). Raw sequencing data have been deposited in the National Center for Biotechnology Information (NCBI).

RNA-seq analysis of DADS-treated microorganisms and ROS staining verification

RNA-seq analysis was conducted as follows: Ten fungal blocks of *C. destructans* or *P. allii* were inoculated into 100 ml of potato glucose liquid culture medium and pre-incubated on a rotary shaker at 140 rpm and 28°C for 120 h. The medium was supplemented with DADS methanol solution at final concentrations of 13.7 or 273.5 μM for *P. allii*, 273.5 μM for *C. destructans*, and 683.7 μM for *P. capsici*. Methanol (1%, v/v) served as the control. Following 24 h of continuous shaking at 28°C, the supernatant was discarded, and mycelia were immediately flash-frozen in liquid nitrogen and stored at −80°C for subsequent RNA extraction. Total RNA was isolated using TRIzol Reagent according to the manufacturer's instructions. A cDNA library was prepared from enriched cDNA and sequenced on an Illumina NovaSeq 6000 platform using paired-end 150-bp reads. Differentially expressed genes (DEGs) between control and DADS-treated samples were identified by quantifying transcript levels via the transcripts per million (TPM) method. Statistical analysis was performed using DESeq2, with significance thresholds set at $|\log_2$ fold change (FC)$| > 2$ and an adjusted p-value <0.05. To validate DEGs, RT–qPCR was conducted using primers listed in Supplemental Table 8. Gene expression was normalized to 18S rRNA (for *P. allii* and *C. destructans*) or *Actin-1* (for *P. capsici*) as reference genes, and relative quantification was calculated using the $2^{-\Delta\Delta CT}$ method. DEGs were functionally annotated through the KEGG pathway database, and pathway enrichment analysis was performed to elucidate metabolic perturbations (Wu et al., 2024).

ROS generation was visualized using DCFH-DA, a ROS-specific fluorescent dye (Solarbio, China). Conidial or zoospore suspensions were pre-inoculated in potato dextrose liquid medium for 120 h in shaking culture (140 rpm) at 28°C. Subsequently, DADS at concentrations of 0, 13.7, 273.5, and 683.7 μM was separately added to the medium for 24 h at 28°C. After treatment, developed mycelia were incubated with DCFH-DA for 25 min at 28°C in the dark, followed by three washes with sterile water prior to microscopic observation. ROS staining was observed using fluorescence microscopy (Leica DM2000, Germany) (Pan et al., 2023; Wu et al., 2024). In addition, ROS fluorescence intensity was quantitatively detected with a Varioskan LUX multimode microplate reader (Thermo Fisher Scientific). Excitation and emission wavelengths were set to 488 nm and 525 nm, respectively (Wu et al., 2024).

Exogenous DADS and H_2O_2 application to soil to simulate garlic-conditioned soil

The DADS concentration range was determined through root exudation analysis, and two experimental series were conducted using the rhizosphere bag method (Supplemental Figure 34). Soil treatments included four DADS concentration gradients (D1, D2, D3, and D4 representing soil DADS concentrations of 13.7, 27.3, 54.7, and 109.4 μmol kg^{-1}, respectively) and four H_2O_2 concentration gradients (H1, H2, H3, and H4 representing soil H_2O_2 concentrations of 85.9, 171.7, 343.4, and 686.9 μmol kg^{-1}, respectively), along with an untreated control group. DADS was applied to evaluate the effects of sulfur-containing compounds on soil microbiota, while H_2O_2 was used to assess the impacts of ROS on soil microbial communities. DADS emulsifiable concentrate (0.005% Tween-20) and H_2O_2 aqueous solution were applied twice to the rhizosphere bags on day 0 and day 15. Soil samples were collected after 30 days of incubation under controlled conditions (25°C ± 1°C, 60% field capacity) for subsequent analysis (Figures 3D and 5A).

Field experiments

Field yield experiments were conducted in Qilin District, Yunnan Province, China (103.86°E, 25.41°N). Each plot measured 6 m × 1 m (length × width), and pepper or soybean seedlings were randomly selected from each plot for yield assessment. Each treatment consisted of five replicate plots. Greenhouse experiments were conducted in Xundian County, Yunnan Province, China (103.29°E, 25.51°N) to evaluate disease control effectiveness. Each plot measured 1.5 m × 1.3 m (length × width), and disease incidence of *P. capsici* and *Phytophthora parasitica* var. *nicotianae* was assessed. Each plot contained nine pepper or tobacco seedlings, with five replicate plots per treatment. Both experiments included six treatments: control (CK); YNAU-Q-6 alone; 13.7 μM DADS; 13.7 μM DADS combined with YNAU-Q-6; 273.5 μM DADS; and

273.5 μM DADS combined with YNAU-Q-6. The concentration of *P. allii* YNAU-Q-6 spores was set to 10^6 colony-forming units (CFU) ml^{-1}.

The colonization effects of *Penicillium* in the pepper rhizosphere were evaluated under six treatment conditions: control (CK); *P. allii* YNAU-Q-6 alone; single application of 273.5 μM DADS; double application of 273.5 μM DADS; single application of 273.5 μM DADS combined with YNAU-Q-6; and double application of 273.5 μM DADS combined with YNAU-Q-6. Quantification of *Penicillium* colonization was performed using an ITS-based absolute quantification approach. This involved constructing a standard curve through correlation analysis between known concentrations of spike-in DNA (expressed as copies per microliter) and corresponding sequencing abundance values obtained via high-throughput sequencing. The absolute copy numbers of ASVs in each experimental sample were subsequently determined by applying the obtained sequence counts to the standard curve equation, as described by Wang et al. (2024).

$$Copy\ number\ (g^{-1}) = \frac{\text{ASV total copy number} \times \text{extracted DNA (ng)}}{\text{template DNA (ng)} \times \text{environmental samples (g)}}$$

Statistical analysis

Metabolome analysis was performed using the MetaboAnalyst cloud platform. Microbial high-throughput sequencing, metagenomics, and transcriptome analyses were conducted on the Meiji Bio Cloud Platform and MicrobiomeAnalyst. The statistical significance of differences between treatments was analyzed using one-way ANOVA (for three or more groups) or a *t*-test (for two groups) in SPSS Statistics 19 (IBM, USA). A significance threshold of $p < 0.05$ was applied for all analyses. Data visualization was conducted using GraphPad Prism 8 (GraphPad Software, USA), Hiplot (https://hiplot.com.cn), and OmicStudio (https://www.omicstudio.cn).

DATA AVAILABILITY

Raw sequencing data (fasta format) have been deposited in the NCBI Sequence Read Archive under BioProject accession numbers PRJNA1230841 (microbiome), PRJNA1232338 (transcriptome), and PRJNA1233622 (metagenome). Raw sequencing data of microbiome (CSTR: 31 253.11.sciencedb.27280) and transcriptome (CSTR: 31 253.11.sciencedb.27286) are also presented in ScienceDB (https://www.scidb.cn/).

FUNDING

This work was supported by the National Key Research and Development Program of China (2023YFE0107500), National Natural Science Foundation of China (32260706), Colorful Yunnan Postdoctoral Program (A3012025211), and Gansu Province Postdoctoral Special Project (25JRRG024).

ACKNOWLEDGMENTS

The valuable comments provided by Jean-Benoit Morel and Junfeng Liu from the International Associated Laboratory of China and France in Agriculture are gratefully acknowledged. No conflict of interest is declared.

AUTHOR CONTRIBUTIONS

S.Z., J.W., Y.L., and M.Y. conceived and designed the study; J.W., Y.L., H. Y., and M.Y. performed the experiments and analyzed the data; S.Z. and J.W. wrote the manuscript. All authors edited the manuscript and approved the final version.

SUPPLEMENTAL INFORMATION

Supplemental information is available at *Plant Communications Online*.

Received: April 12, 2025
Revised: June 7, 2025
Accepted: September 1, 2025
Published: September 3, 2025

REFERENCES

Cavagnaro, P.F., Camargo, A., Piccolo, R.J., Lampasona, S.G., Burba, J.L., and Masuelli, R.W. (2005). Resistance to *Penicillium hirsutum* Dierckx in garlic accessions. Eur. J. Plant Pathol. **112**:195–199. https://doi.org/10.1007/s10658-005-1750-6.

Chen, X.C., Xu, J.R., Gao, P.Y., Luo, Z.D., Zheng, Z.S., Wu, B.C., Zhao, Y., Huang, Z.B., Chen, H.B., and Zhang, Q.F. (2023). Identification and inhibitory effect on *Lasiodiplodia theobromae* of Actinomycetes in *Camellia sinensis* rhizosphere soil. Fujian J. Agr. Sci. **38**:1103–1111. https://doi.org/10.19303/j.issn.1008-0384.2023.09.012.

Cheng, F., Cheng, Z., Meng, H., and Tang, X. (2016). The garlic allelochemical diallyl disulfide affects tomato root growth by influencing cell division, phytohormone balance and expansin gene expression. Front. Plant Sci. **7**:1199. https://doi.org/10.3389/fpls.2016.01199.

Desmedt, W., Kudjordjie, E.N., Chavan, S.N., Zhang, J., Li, R., Yang, B., Nicolaisen, M., Mori, M., Peters, R.J., Vanholme, B., et al. (2022). Rice diterpenoid phytoalexins are involved in defense against parasitic nematodes and shape rhizosphere nematode communities. New Phytol. **235**:1231–1245. https://doi.org/10.1111/nph.18152.

Ding, H., Ali, A., and Cheng, Z. (2018). Dynamics of a soil fungal community in a three-year green garlic/cucumber crop rotation system in Northwest China. Sustainability **10**:1391. https://doi.org/10.3390/su10051391.

Dorrance, A.E. (2018). Management of *Phytophthora sojae* of soybean: a review and future perspectives. Can. J. Plant Pathol. **40**:210–219. https://doi.org/10.1080/07060661.2018.1445127.

Duan, Y.M., Zhang, T.J., Xu, X.Y., Fang, R.Q., Yang, Z.Y., Wang, J.G., Li, X.J., and Chen, S.Y. (2014). Effect of soil texture on stress resistance of flue-cured tobacco induced by dry mycelium of *Penicillium chrysogenum*. Southwest China J. Agr. Sci. **27**:2449–2454. https://doi.org/10.16213/j.cnki.scjas.2014.06.037.

Gu, Y., Dong, K., Geisen, S., Yang, W., Yan, Y., Gu, D., Liu, N., Borisjuk, N., Luo, Y., and Friman, V.P. (2020). The effect of microbial inoculant origin on the rhizosphere bacterial community composition and plant growth-promotion. Plant Soil **452**:105–117. https://doi.org/10.1007/s11104-020-04545-w.

Guo, T., Li, C., Zhao, Y., Huang, X., Luo, Z., Li, H., Liu, A., Ahammed, G. J., and Chen, S. (2024). Cytochrome P450 CYP736A12 is crucial for *Trichoderma asperellum*-induced alleviation of phoxim phytotoxicity and reduction of pesticide residue in tomato roots. J. Hazard. Mater. **471**:134299.

He, S., Lv, M., Wang, R., Li, N., Wang, T., Shi, W., Gao, Z., and Li, X. (2024). Long-term garlic−maize rotation maintains the stable garlic rhizosphere microecology. Environ. Microbiome **19**:90. https://doi.org/10.1186/s40793-024-00636-8.

Hong, S., Yuan, X., Yang, J., Yang, Y., Jv, H., Li, R., Jia, Z., and Ruan, Y. (2023). Selection of rhizosphere communities of diverse rotation crops reveals unique core microbiome associated with reduced banana *Fusarium* wilt disease. New Phytol. **238**:2194–2209. https://doi.org/10.1111/nph.18816.

Ji, X.Y., Ye, C., Kang, W., Luan, W., Liu, Y., He, X., Yang, M., Sun, L., Sun, W., Huang, H., et al. (2025). Interspecific allelopathic interaction primes direct and indirect resistance in neighboring plants within agroforestry systems. Plant Commun. **6**:101173. https://doi.org/10.1016/j.xplc.2024.101173.

Kamoun, S., Furzer, O., Jones, J.D.G., Judelson, H.S., Ali, G.S., Dalio, R.J.D., Roy, S.G., Schena, L., Zambounis, A., Panabières, F., et al. (2015). The Top 10 oomycete pathogens in molecular plant pathology. Mol. Plant Pathol. **16**:413–434. https://doi.org/10.1111/mpp.12190.

Li, X., Chen, D., Carrión, V.J., Revillini, D., Yin, S., Dong, Y., Zhang, T., Wang, X., and Delgado-Baquerizo, M. (2023). Acidification suppresses the natural capacity of soil microbiome to fight pathogenic *Fusarium* infections. Nat. Commun. **14**:5090. https://doi.org/10.1038/s41467-023-40810-z.

Liu, H., Wu, J., Su, Y., Li, Y., Zuo, D., Liu, H., Liu, Y., Mei, X., Huang, H., Yang, M., and Zhu, S. (2021). Allyl isothiocyanate in the volatiles of *Brassica juncea* inhibits the growth of root rot pathogens of *Panax notoginseng* by inducing the accumulation of ROS. J. Agric. Food Chem. **69**:13713–13723. https://doi.org/10.1021/acs.jafc.1c05225.

Liu, S., Tao, C., Zhang, L., Wang, Z., Xiong, W., Xiang, D., Sheng, O., Wang, J., Li, R., Shen, Z., et al. (2023). Plant pathogen resistance is mediated by recruitment of specific rhizosphere fungi. ISME J. **17**:931–942. https://doi.org/10.1038/s41396-023-01406-z.

Liu, Y., Xu, Z., Chen, L., Xun, W., Shu, X., Chen, Y., Sun, X., Wang, Z., Ren, Y., Shen, Q., and Zhang, R. (2024). Root colonization by beneficial rhizobacteria. FEMS Microbiol. Rev. **48**:fuad066. https://doi.org/10.1093/femsre/fuad066.

Luo, L., Wang, L., Deng, L., Mei, X., Liu, Y., Huang, H., Du, F., Zhu, S., and Yang, M. (2021). Enrichment of *Burkholderia* in the rhizosphere by autotoxic ginsenosides to alleviate negative plant-soil feedback. Microbiol. Spectr. **9**:e0140021. https://doi.org/10.1128/Spectrum.01400-21.

Luo, L., Zhang, J., Ye, C., Li, S., Duan, S., Wang, Z., Huang, H., Liu, Y., Deng, W., Mei, X., et al. (2022). Foliar pathogen infection manipulates soil health through root exudate-modified rhizosphere microbiome. Microbiol. Spectr. **10**:e0241822. https://doi.org/10.1128/spectrum.02418-22.

Ma, H.K., Pineda, A., Hannula, S.E., Kielak, A.M., Setyarini, S.N., and Bezemer, T.M. (2020). Steering root microbiomes of a commercial horticultural crop with plant-soil feedbacks. Appl. Soil Ecol. **150**:103468. https://doi.org/10.1016/j.apsoil.2019.103468.

Madhushan, A., Weerasingha, D.B., Ilyukhin, E., Taylor, P.W.J., Ratnayake, A.S., Liu, J.K., and Maharachchikumbura, S.S.N. (2025). From natural hosts to agricultural threats: The evolutionary journey of phytopathogenic fungi. J. Fungi (Basel) **11**:25. https://doi.org/10.3390/jof11010025.

Mukhovi, S., and Jacobi, J. (2022). Can monocultures be resilient? Assessment of buffer capacity in two agroindustrial cropping systems in Africa and South America. Agr. Food Secur **11**:19. https://doi.org/10.1186/s40066-022-00356-7.

Mullins, A.J., Murray, J.A.H., Bull, M.J., Jenner, M., Jones, C., Webster, G., Green, A.E., Neill, D.R., Connor, T.R., Parkhill, J., et al. (2019). Genome mining identifies cepacin as a plant-protective metabolite of the biopesticidal bacterium *Burkholderia ambifaria*. Nat. Microbiol. **4**:996–1005. https://doi.org/10.1038/s41564-019-0383-z.

Ning, D., Wang, Y., Fan, Y., Wang, J., Van Nostrand, J.D., Wu, L., Zhang, P., Curtis, D.J., Tian, R., Lui, L., et al. (2024). Environmental stress mediates groundwater microbial community assembly. Nat. Microbiol. **9**:490–501. https://doi.org/10.1038/s41564-023-01573-x.

Ostfeld, R.S., and Keesing, F. (2012). Effects of Host Diversity on Infectious Disease. Annu. Rev. Ecol. Evol. Syst. **43**:157–182. https://doi.org/10.1146/annurev-ecolsys-102710-145022.

Pan, C., Yang, K., Erhunmwunsee, F., Li, Y.X., Liu, M., Pan, S., Yang, D., Lu, G., Ma, D., and Tian, J. (2023). Inhibitory effect of cinnamaldehyde on *Fusarium solani* and its application in postharvest preservation of sweet potato. Food Chem. **408**:135213. https://doi.org/10.1016/j.foodchem.2022.135213.

Pérez-Brandán, C., Huidobro, J., Grümberg, B., Scandiani, M.M., Luque, A.G., Meriles, J.M., and Vargas-Gil, S. (2014). Soybean fungal soil-borne diseases: a parameter for measuring the effect of agricultural intensification on soil health. Can. J. Microbiol. **60**:73–84. https://doi.org/10.1139/cjm-2013-0792.

Pokou, N.D., N'Goran, J.A.K., Kébé, I., Eskes, A., Tahi, M., and Sangaré, A. (2008). Levels of resistance to *Phytophthora* pod rot in cocoa accessions selected on-farm in Côte d'Ivoire. Crop Prot. **27**:302–309. https://doi.org/10.1016/j.cropro.2007.07.012.

Qian, Y.L., Hua, G.K.H., Scott, J.C., Dung, J.K.S., and Qian, M.C. (2022). Evaluation of sulfur-based biostimulants for the germination of *Sclerotium cepivorum* sclerotia and their interaction with soil. J. Agric. Food Chem. **70**:15038–15045. https://doi.org/10.1021/acs.jafc.2c05862.

Sánchez-Gil, J.J., Poppeliers, S.W.M., Vacheron, J., Zhang, H., Odijk, B., Keel, C., and de Jonge, R. (2023). The conserved iol gene cluster in *Pseudomonas* is involved in rhizosphere competence. Curr. Biol. **33**:3097–3110. https://doi.org/10.1016/j.cub.2023.05.057.

Shang, J., Liu, X.F., Adili, S., Pan, C.D., Zhao, Z.Y., and Ma, H.X. (2010). Pathogen identification of walnut stalk rot disease. Sci. Silvae Sin. **46**:97–100.

Simonin, M., Briand, M., Chesneau, G., Rochefort, A., Marais, C., Sarniguet, A., and Barret, M. (2022). Seed microbiota revealed by a large-scale meta-analysis including 50 plant species. New Phytol. **234**:1448–1463. https://doi.org/10.1111/nph.18037.

Upadhyay, S.K., Srivastava, A.K., Rajput, V.D., Chauhan, P.K., Bhojiya, A.A., Jain, D., Chaubey, G., Dwivedi, P., Sharma, B., and Minkina, T. (2022). Root exudates: mechanistic insight of plant growth promoting rhizobacteria for sustainable crop production. Front. Microbiol. **13**:916488. https://doi.org/10.3389/fmicb.2022.916488.

Voges, M.J.E.E.E., Bai, Y., Schulze-Lefert, P., and Sattely, E.S. (2019). Plant-derived coumarins shape the composition of an Arabidopsis synthetic root microbiome. Proc. Natl. Acad. Sci. USA **116**:12558–12565. https://doi.org/10.1073/pnas.1820691116.

Wang, G., Bei, S., Li, J., Bao, X., Zhang, J., Schultz, P.A., Li, H., Li, L., Zhang, F., Bever, J.D., and Zhang, J. (2021a). Soil microbial legacy drives crop diversity advantage: Linking ecological plant-soil feedback with agricultural intercropping. J. Appl. Ecol. **58**:496–506. https://doi.org/10.1111/1365-2664.13802.

Wang, H.Y., Zhao, L., Jiang, W.T., Zhang, R., Chen, R., Mao, Y.F., Chen, X.S., Shen, X., Yin, C.M., and Mao, Z.Q. (2021b). Effects of *Allium fistulosum-Brassica juncea-Triticum aestivum rotation* a year on the soil microbial environment and the subsequent growth of young apple trees. Sci. Hortic. **290**:110549. https://doi.org/10.1016/j.scienta.2021.110549.

Wang, M., Ge, A.H., Ma, X., Wang, X., Xie, Q., Wang, L., Song, X., Jiang, M., Yang, W., Murray, J.D., et al. (2024). Dynamic root microbiome sustains soybean productivity under unbalanced fertilization. Nat. Commun. **15**:1668. https://doi.org/10.1038/s41467-024-45925-5.

Wen, T., Ding, Z., Thomashow, L.S., Hale, L., Yang, S., Xie, P., Liu, X., Wang, H., Shen, Q., and Yuan, J. (2023). Deciphering the mechanism of fungal pathogen-induced disease-suppressive soil. New Phytol. **238**:2634–2650. https://doi.org/10.1111/nph.18886.

Wu, J., Liu, J., Sun, J., Liu, Y., He, T., Zhao, J., Mei, X., Liu, Y., Yang, M., and Zhu, S. (2024). Diallyl Trisulfide acts as a soil disinfestation against the *Ilyonectria destructans* through inducing the burst of reactive oxygen species. J. Agric. Food Chem. **72**:9669–9679. https://doi.org/10.1021/acs.jafc.4c01422.

Xiong, C., Zhu, Y.G., Wang, J.T., Singh, B., Han, L.L., Shen, J.P., Li, P. P., Wang, G.B., Wu, C.F., Ge, A.H., et al. (2021). Host selection shapes crop microbiome assembly and network complexity. New Phytol. **229**:1091–1104. https://doi.org/10.1111/nph.16890.

Xu, Z., Xu, X., Wang, Y., Liu, L., Li, Y., Yang, Y., Liu, L., Zou, L., and Chen, G. (2022). A varied AvrXa23-like TALE enables the bacterial blight pathogen to avoid being trapped by Xa23 resistance gene in rice. J. Adv. Res. **42**:263–272. https://doi.org/10.1016/j.jare.2022.01.007.

Yang, M., Zhang, Y., Qi, L., Mei, X., Liao, J., Ding, X., Deng, W., Fan, L., He, X., Vivanco, J.M., et al. (2014). Plant-plant-microbe mechanisms involved in soil-borne disease suppression on a maize and pepper intercropping system. PLoS One **9**:e115052. https://doi.org/10.1371/journal.pone.0115052.

Yang, Q., Yang, J., Wang, Y., Du, J., Zhang, J., Luisi, B.F., and Liang, W. (2022). Broad-spectrum chemicals block ROS detoxification to prevent plant fungal invasion. Curr. Biol. **32**:3886–3897. https://doi.org/10.1016/j.cub.2022.07.022.

Yang, X.P., Du, D., Zhang, M.Z., Z.., et al. (2025). Pathogenic fungi accumulation rather than direct autotoxicity is responsible for the root-rot disease of foxtail millet under continuous monoculture regimes. Biol. Fert. Soils **61**:877–892. https://doi.org/10.1007/s00374-025-01904-6.

Yin, C.F., Pan, P., Li, T., Song, X., Xu, Y., and Zhou, N.Y. (2025). The universal accumulation of p-aminophenol during the microbial degradation of analgesic and antipyretic acetaminophen in WWTPs: a novel metagenomic perspective. Microbiome **13**:68. https://doi.org/10.1186/s40168-025-02065-2.

Zhang, Y., Ye, C., Su, Y., Peng, W., Lu, R., Liu, Y., Huang, H., He, X., Yang, M., and Zhu, S. (2022). Soil Acidification caused by excessive application of nitrogen fertilizer aggravates soil-borne diseases: Evidence from literature review and field trials. Agric. Ecosyst. Environ. **340**:108176. https://doi.org/10.1016/j.agee.2022.108176.

Zhou, X., Zhang, J., Shi, J., Khashi u Rahman, M., Liu, H., Wei, Z., Wu, F., and Dini-Andreote, F. (2024). Volatile-mediated interspecific plant interaction promotes root colonization by beneficial bacteria via induced shifts in root exudation. Microbiome **12**:207. https://doi.org/10.1186/s40168-024-01914-w.

Zhou, Y., Yang, Z., Liu, J., Li, X., Wang, X., Dai, C., Zhang, T., Carrión, V.J., Wei, Z., Cao, F., et al. (2023). Crop rotation and native microbiome inoculation restore soil capacity to suppress a root disease. Nat. Commun. **14**:8126. https://doi.org/10.1038/s41467-023-43926-4.

Zhuang, L., Li, Y., Wang, Z., Yu, Y., Zhang, N., Yang, C., Zeng, Q., and Wang, Q. (2021). Synthetic community with six *Pseudomonas* strains screened from garlic rhizosphere microbiome promotes plant growth. Microb. Biotechnol. **14**:488–502. https://doi.org/10.1111/1751-7915.13640.

Plant Communications, Volume *6*

Supplemental information

An altruistic rhizo-microbiome strategy in crop-rotation systems for sustainable management of soil-borne diseases

Jiaqing Wu, Yixiang Liu, Huanjie Yu, Fuyuan Fan, Xiahong He, Youyong Zhu, Yang Dong, Min Yang, and Shusheng Zhu

Supplemental Methods

Meta-analysis

Literatures were searched in Google Scholar and CNKI using “garlic” and “crop rotation” as the primary keywords. Papers were selected according to the following criteria (Zhang et al., 2022): (a) the experimental type must be a field or pot experiment; (b) the experimental research parameters must include crop yield; (c) the mean, standard deviation (or standard error) and sample size must be available or calculable. For each selected study, the original data were collected directly from the tables and texts. If the data appeared in the form of graphics, the required data were obtained online using WebPlotDigitizer (https://apps.automeris.io/wpd/index.zh_CN.html). If there is no standard error (SE), SE is calculated as SE=1/4×mean (Dynarski et al., 2018). If the data provided in the literature is the standard error (SE), the standard deviation (SD) can be converted using the following equation:

$$SD=SE\sqrt{n}, \quad ①$$

where n = number of replicates.

According to the above screening criteria, a total of 21 articles were finally obtained (Supplemental Table 2), and 70 experimental data were used for meta-analysis. The garlic planting group was used as the treatment group, and any treatment without garlic, including fallow or wheat planting (winter wheat planting time is similar to garlic) or continuous planting as the control group, the average yield, standard deviation and number of replicates of the subsequent crops in each study were extracted to calculate the effect size lnR (Zheng et al., 2019):

$$\ln R=\ln(Y_e/Y_c), \quad ②$$

Y_e is the yield of the experimental group; Y_c is the yield of the control group. The yield units of field trials are uniformly converted to kg hm^{-2}, and the yield units of pot trials are uniformly converted to g $plant^{-1}$.

The mean and variance of lnR were analyzed by MetaWin 3.0.14 software (http://www.metawinsoft.com). The random effect size and variance were calculated, and the global effect size was further calculated using MetaWin 3.0.14 software with a confidence interval (CI) of 95%. The significance level was reached when the 95% CI crossed the zero line.

Due to differences in geographical factors, climatic conditions, field management measures, and

soil fertility among the studies, there was a large variability in the research results, so a random effects model was selected for analysis.

This study used the heterogeneity between groups ($Q_{Between}$) to test the difference in the effect size of the same indicator between different subgroups and to analyze the heterogeneity between groups. If $p < 0.05$, the heterogeneity between groups is considered significant; otherwise, it is considered insignificant (Nie et al., 2023).

The data were tested for publication bias using Egger's method. If the Egger's regression result $p > 0.05$, the data were considered reliable (Nie et al., 2023).

Plant materials

Garlic (*Allium sativum*, purple-skinned variety) used in this study was sourced from a local market in Yunnan. The pepper (*Capsicum annuum*) used in the indoor and field experiments was six-color pickled pepper F1 (Guangxi Hengxian Zilong Seed Industry Co., Ltd.), the tobacco was *Nicotiana benthamiana*, and the potato was purple potato.

Collection of garlic-conditioned soil

Prior to sowing, the garlic was subjected to surface disinfection using a 1.5% sodium hypochlorite solution. To facilitate the collection of conditioned soil for subsequent experiments, a root bag method was employed. The procedure involved placing a nylon root bag (upper diameter of 11 cm and height 10 cm) inside each plastic flower pot (inner diameter of 13.5 cm and height of 14.5 cm). Each bag was filled with 600 g of soil, while an additional 1.2 kg of soil was packed into the gap between the bag and the pot. This setup created two distinct compartments: the interior of the bag, which constituted the rhizosphere, and the area between the bag and the pot, representing the root circumference. The root bag allowed the penetration of small molecular compounds while preventing root intrusion (Supplemental Figure 34).

The planting trial was conducted over two seasons. In the first season, garlic-conditioned soil was collected to evaluate its effects on the growth of three Solanaceous crops (pepper, tobacco, potato) and the incidence of *Phytophthora* blight in peppers. The second season focused on further assessment the impacts of garlic-conditioned soil with varying planting density on the blight as well as the composition of soil microbial communities (Ding et al., 2018; Guo et al., 2019).

One to five cloves of garlic were sown in each bag, while soil treated similarly but without any planted crops served as a control. After 30 days of regular watering, the garlic rhizosphere soil and control soil were collected separately. Each replicate consisted of four pots, which were subsequently combined into a single composite sample for analysis.

Inoculation of *Phytophthora capsici* on the stem of pepper

Method for inoculating *P. capsici* by scratching the base of pepper seedling stems: a sterilized blade was used to create a wound on the epidermis of the stem base of the pepper seedling, approximately 7 mm in length and located close to the soil. A 7 mm diameter cake was then applied to the incision surface, followed by a layer of absorbent cotton. 1 mL of sterile water was injected into the absorbent cotton to moisten it (Yu et al., 2024).

Pepper metabolite extraction and analysis

The changes in metabolites of pepper were measured following previous report with some modifications (Liu et al., 2023). The leaves of pepper treated with garlic soil and garlic soil-filtered (CK) were ground in liquid nitrogen, respectively, which were used for metabolite extraction. 60 mg samples were weighed and ultrasonically extracted at 37°C for 30 min with 1 mL of extraction solution prepared by methanol, chloroform and sterilized deionized water in a volume ratio of 5:2:2. Then the homogenates were centrifuged for 3 min (1600 g, 4°C). After that, the supernatant was transferred and dried using a SpeedVac (Christ, Germany) at 25°C. A total of 80 μL of 20 mg·mL^{-1} methoxyamine hydrochloride solution dissolved in pyridine and 40 μL of N-methyl-N-(trimethylsilyl)-trifluoroacetamide were added to the dried samples in two steps, and reacted for 90 min, 30°C and 30 min, 37°C respectively for derivatization. Finally, each sample was centrifuged for 3 min (1600 g, 4°C) and stored at 4°C for metabolite analysis. Based on a previously reported method, gas chromatography−mass spectrometry (GC−MS; QP2010 Ultra, Shimadzu, Kyoto, Japan) was used to analyse the metabolites of pepper. The model of chromatographic column was SH-Rxi-5Sil MS and 30.0 m × 0.25 mm × 0.25 μm. The offline data was first converted to abf format using Analysis Base File Converter, and then processed by MSDIAL for peak search, peak alignment, and identification of metabolite ion peaks. Based on a fold change (FC) >1.5 or <0.67 and $p < 0.05$, the differentially accumulated metabolites (DAMs) were screened (Liu et al., 2023).

Isolation, identification and functional verification of garlic-conditioned **soil microorganisms**

5 grams of garlic-conditioned soil samples from different densities were placed into 50 mL sterile centrifuge tubes. Then, 45 mL of sterile water was added, and the mixture was shaken at 120 rpm for 30 minutes. The solution was allowed to stand for 15 minutes to create a soil suspension, which was subsequently diluted 100-fold with sterile water. A volume of 50 μL of this suspension was spread evenly onto the surface of various Bengal red solid culture media and incubated at 28°C in a constant temperature incubator. Single colonies were selected from the corresponding plates for purification. Following the method of Visagie et al., 2014, single colonies were observed on both sides of PDA medium for colony shape, color, texture, and colony margins. Conidia and sporangia morphology were examined under a light microscope. PCR amplification was conducted using ITS and β-tubulin (*TUB2*) primers. The amplified products were purified and subjected to Sanger sequencing. The sequencing results were compared against the GenBank database via BLAST analysis. A phylogenetic tree was constructed with MEGA11 software using the neighbor-joining method. The molecular and morphological identification results were integrated to determine the pathogen's species.

An antagonistic test was conducted between isolated fungi and *P. capsici*. Different fungi were activated, and a 7 mm diameter *P. capsici* plug was inoculated at the center of the PDA medium. The plugs of the test strains were then inoculated 22 mm away from the *P. capsici* plugs. As a control, *P. capsici* was inoculated only in the center of the plate. Each strain was replicated four times. Inhibition rate (%) = (average diameter of control treatment − average diameter of experimental treatment)/(average diameter of control treatment) × 100.

Induced resistance test for pepper disease. The isolated fungi plug was placed in liquid PDA medium and cultured at 26°C for 5-7 days until the hyphae covered the medium. It was then broken down using a juicer and diluted five times with water for subsequent use. The base of the pepper stem was inoculated with the blight; the fungal fermentation liquid was applied once prior to inoculation and again following inoculation. The length of the lesions was measured 7 days post-inoculation. Three pepper plants were inoculated per pot, with each pot serving as a replicate, and four replicates were included for each treatment. Additionally, 24 hours after fungal treatment, the aboveground parts of pepper seedlings were harvested to detect salicylic acid pathway resistance genes *(PAL* and *PR1c*; primer sequences provided in Supplemental Table 9), as described by Diao

et al. (2019), Zhao et al. (2022), and Zhang et al. (2020). Salicylic acid of pepper seedlings were quantified according to Luo et al. (2025).

Three *Penicillium* strains (*P. allii* YNAU-Q-6, *P. ochrochloron* YNAU-P-4, and *P. brevicompactum* YNAU-Q-9) were evaluated using 5-mm mycelial plugs from 7-day-old PDA cultures. Garlic seedlings and uniform cloves (30-35 mm) were surface-sterilized with 1% NaClO (3 min), then triple-rinsed. Standardized 5-mm wounds were inoculated with 5-mm fungal plugs, with PDA-only and non-wounded controls. Incubation at 28°C for 3 days allowed daily disease assessment. Rot severity was observed by lesion diameter. In addition, the same method was used to detect the effect of *Penicillium* on the disease resistance genes of garlic seedlings (*PAL* and *PR1c*; primer sequences provided in Supplemental Table 9), as described by Tuan et al. (2010) and Anisimova et al. (2021).

GC-MS conditions

GC conditions: HP-5MS chromatography column (30.0m × 0.25mm × 0.25 μm). Starting column temperature: 40 °C, after heating up to 80 °C at 3.0/min, raise the temperature to 270 °C at 5.0 °C/min and maintain it for 10.0 minutes. The carrier gas is helium, with a sample inlet temperature of 250.0 °C and a column box temperature of 40.0 °C. The injection method is direct injection without splitting.

MS conditions: EI ionization source, ion source temperature 230 °C, interface temperature 250 °C, scanning range m/z 35-500, acquisition method Scan, scanning interval 0.30s. The identification of volatile components in roots was carried out by searching the retention time in the NIST14/ NIST14s spectrogram library. Garlic sulfide compounds were determined by comparing the characteristic fragment ions with the characteristic fragment ions of sulfide compound standards (Wu et al., 2024).

Effect of DADS and H_2O_2 treatment for the growth of *P. allii*, *C. destructans* and *P. capsici*

10 blocks of *P. allii, C. destructans and P. capsici* were added to 100 mL of potato glucose liquid culture medium and placed on a shaking table for pre-incubating at 140 rpm and 28°C for 120 hours. The culture medium was supplemented DADS with final concentrations of0, 13.7, 27.3, 54.7, 109.4, 683.7 μM or H_2O_2 with final concentrations of 85.9, 1717.1, 4292.9μM. After being subjected to continuous shaking cultivation at 28 °C for 72 hours. Collecting the mycelial balls and dry them in an oven for weight (Wu et al., 2024).

Detection of Thiamine Utilization by Soil Microorganisms

Five grams of soil from different treatments (CK, Z3, D3, and H3) were weighed, and 45 mL of ddH_2O was added. The mixture was shaken at 150 rpm for 30 min to prepare a soil suspension. The suspension was filtered through filter paper, and the filtrate was centrifuged at 3000 rpm and 4°C for 10 min, with the supernatant discarded. The pellet was resuspended in 20 mL of 296.5 μM thiamine solution and shaken at 150 rpm for 24 h. The mixture was reacted with the test solution for 10 min before and after the 24 h shaking period. The OD_{704} values were measured before and after fermentation using a microplate reader (Li et al., 2022). Thiamine utilization efficiency was calculated using the following formula:

$$\frac{\text{Initial absorbance value} - \text{Absorbance value after 24 hours}}{\text{Initial absorbance value} - \text{Blank absorbance value}} \times 100\%$$

Supplemental Table 1 Fungi and Bacteria analysis of similarities (ANOSIM) in garlic treatment

		Garlic VS CK		Z1 VS CK		Z2 VS CK		Z3 VS CK		Z5 VS CK	
	ANOSIM	R	*p*-value	R	*p*-value	R	*p*-value	R	*p*-value	R	*p*-value
Genus	Fungi	0.3056	0.043	0.1042	0.065	0.2604	0.040	0.3646	0.040	0.8854	0.040
	Bacteria	0.0881	0.249	-0.1563	0.86	-0.125	0.767	-0.0625	0.625	0.03125	0.346
ASV	Fungi	0.3388	0.011	0.2360	0.023	0.1680	0.028	0.4960	0.015	0.9560	0.015
	Bacteria	0.1550	0.164	-0.2292	0.949	-0.0625	0.617	-0.0313	0.535	0.3542	0.115

Note: Z1, Z2, Z3, and Z5 represent 1, 2, 3, and 5 garlic plants per pot, respectively. CK represents control group.

Supplemental Table 2 Fungi analysis of similarities (ANOSIM) in DADS treatment

	DADS VS CK		D1 VS CK		D2 VS CK		D3 VS CK		D4 VS CK	
ANOSIM	R	*p*-value	R	*p*-value	R	*p*-value	R	*p*-value	R	*p*-value
Genus	0.4896	0.021	0.0625	0.240	0.0313	0.290	0.0521	0.205	-0.0417	0.623
ASV	0.4063	0.023	0.0360	0.325	0.1520	0.064	0.1240	0.080	-0.0440	0.617

Note: D1, D2, D3, and D4 represent soil DADS concentrations of 13.7, 27.3, 54.7, and 109.4 μmol kg^{-1}, respectively. CK represents control group.

Supplemental Table 3 Fungi analysis of similarities (ANOSIM) in H_2O_2 treatment

	H_2O_2 VS CK		H1 VS CK		H2 VS CK		H3 VS CK		H4 VS CK	
ANOSIM	R	*p*-value	R	*p*-value	R	*p*-value	R	*p*-value	R	*p*-value
Genus	0.6223	0.005	0.1354	0.092	0.1771	0.040	0.0833	0.178	0.1667	0.069
ASV	0.6040	0.002	0.1560	0.057	0.2160	0.027	-0.0160	0.522	0.2040	0.015

Note: H1, H2, H3, and H4 represent soil H_2O_2 concentrations of 85.9, 171.7, 343.4, and 686.9 μmol kg^{-1}, respectively. CK represents control group.

Supplemental Table 4 Volatile components in root exudates of garlic seedlings

Compound Name	Relative peak area%
Diallyl disulfide	44.39418
Dibutyl phthalate	17.38317
1,2-Benzenedicarboxylic acid, bis(2-methylpropyl) ester	8.397252
Oxalic acid, cyclohexylmethyl octadecyl ester	5.223487
Heptacosyl heptafluorobutyrate	4.849501
Sulfurous acid, di(cyclohexylmethyl) ester	3.538899
Bis(2-ethylhexyl) phthalate	3.292318
Sulfurous acid, cyclohexylmethyl octadecyl ester	2.401355
1,2-Benzenedicarboxylic acid, bis(2-methylpropyl) ester	2.222368
Octacosyl pentafluoropropionate	2.130469
Butanoic acid, ethyl ester	1.981014
Phenol, 2,2'-methylenebis[6-(1,1-dimethylethyl)-4-methyl-	1.108266
Propanoic acid, 2-methyl-, ethyl ester	0.699461
5,5,8a-Trimethyldecalin-1-one	0.564236
Sulfurous acid, cyclohexylmethyl pentadecyl ester	0.485706
Heptacosyl heptafluorobutyrate	0.443981
13-Tetradecen-1-ol acetate	0.410959
Heneicosanoic acid, methyl ester	0.240738
erythro-7,8-Bromochlorodisparlure	0.232643

Supplemental Table 5 Volatile compounds in field-collected garlic rhizosphere soil

Compound Name	Relative peak area%
Diallyl disulphide	39.67674
1-Allyl-2-isopropyldisulfane	23.56737
.alpha.-Pinene	9.053534
Disulfide, dipropyl	8.152234
Naphthalene, decahydro-, trans-	2.664853
o-Xylene	2.377539
3-Undecene, 6-methyl-, (E)-	1.990223
3-Hydroxy-3-methylvaleric acid	1.535884
Ethanone, 1-cyclohexyl-	1.349859
Decyl heptyl ether	1.211857
Caryophyllene oxide	1.15235
.beta.-Longipinene	0.957287
Caryophyllene	0.931622
Benzene, 1,4-diethyl-	0.857209
Phenol, 2,2'-methylenebis[6-(1,1-dimethylethyl)-4-methyl-	0.834565
Aciphyllene	0.834091
2H-2,4a-Methanonaphthalene, 1,3,4,5,6,7-hexahydro-1,1,5,5-tetramethyl-, (2S)-	0.763848
Sulfurous acid, 2-ethylhexyl nonyl ester	0.574802
2H,8H-Benzo[1,2-b:5,4-b']dipyran-10-propanoic acid, 5-methoxy-2,2,8,8-tetramethyl-, methyl ester	0.558611
trans-Decalin, 2-methyl-	0.533582
1,2,4-Methenoazulene, decahydro-1,5,5,8a-tetramethyl-, [1S-(1.alpha.,2.alpha.,3a.beta.,4.alpha.,8a.beta.,9R*)]-	0.421932

Supplemental Table 6 Volatile compounds in pot-grown garlic-conditioned soil (Z3 treatment)

Compound Name	Relative peak area%
Diallyl disulfide	56.89708
Dibutyl phthalate	13.42136
2-Ethylhexyl salicylate	11.32036
1,2-Benzenedicarboxylic acid, bis(2-methylpropyl) ester	7.717764
Homosalate	5.15351
2,2,4-Trimethyl-1,3-pentanediol diisobutyrate	1.797997
Sulfurous acid, 2-pentyl undecyl ester	1.739104
Eicosane	0.884293
Heptadecane	0.860879
Cedrol	0.207662

Supplemental Table 7 Meta-analysis data extraction literature

Number	Literature
1	**Yang, J.B., Sun, L.H., Li, Z.M., Guo, W.C., and Guo, D.Q.** (2021). Effects of different varieties and different stubble on *Pinellia ternata* yield. Bull. Agric. Sci. Technol. **0**:181-183. http://tongxun.aiijournal.com/CN/Y2021/V0/I1/181.
2	**Yang, R.P., Mo, Y.L., Liu, C.M., Wang, Y.Q., Ma, J.X., Zhang, Y., Li, H., and Zhang, X.** (2016). The effects of cattle manure and garlic rotation on soil under continuous cropping of watermelon (*Citrullus lanatus* L.). PLoS One **11**:e0156515. https://doi.org/10.1371/journal.pone.0156515.
3	**Dhillon, N.K., Kaur, S., Sidhu, H.S., and Anupam** (2019). Management of root knot nematode opting garlic crop in vegetable based cropping systems. Indian J. Hortic. **76**: 472-478. https://doi.org/10.5958/0974-0112.2019.00075.6.
4	**Ding, H.Y., Ali, A., and Cheng, Z.H.** (2018). Dynamics of a soil fungal community in a three-year green garlic/cucumber crop rotation system in Northwest China. Sustainability **10**:1391. https://doi:10.3390/su10051391.
5	**Li, Z.S., Xiang, Y.H., Jiang, J.Q., Tang, L.W., Teng, Z., and Yan, Q.F.** (2019). A study on the effects of different previous crops on yield and quality of flue-cured tobacco in Panzhihua Area. J. Xichang Univ. (Nat. Sci. Ed.) **33**:10-12. https://doi.org/10.16104/j.issn.1673-1891.2019.01.003.
6	**Liu, W.X., F. X.Y, Zhang F.Y., He, Q,L., Chen, L., Li K., and Wu. J.H.** (2021). Effects of different preceding crops and seed coating agent dosage on peanut diseases, pests yield. Crops **37**:199-204. https://doi.org/10.16035/j.issn.1001-7283.2021.06.032.
7	**Zhang, G.W., Wang, X.Q., Yang, C.Q., Shu, H.M., and Liu, R.X.** (2021). Effects of rotational pattern and fertilization application on soybean yield under straws returning of preceding crop. Chin. J. Eco-Agric. **29**:1493-1501. https://doi.org/10.13930/j.cnki.cjea.210084.
8	**Zhou, Q., Zhang, P., Wang, Z.Q., Wang, L.X., Wang, S.B., Yang, W.T., Yang, B.J., and Huang, G.Q.** (2023). Winter crop rotation intensification to increase rice yield, soil carbon, and microbial diversity. Heliyon **9**:e12903. https://doi.org/10.1016/j.heliyon.2023.e12903.
9	**Wang, T., Chen, H., Zhou, W., Chen, Y., Fu, Y., Yang, Z.P., Liu, Q., Yue, X.P., Deng, F., Nkrumah, M., et al.** (2022). Garlic–rice system increases net economic benefits and reduces greenhouse gas emission intensity. Agric. Ecosyst. Environ.326:107778. https://doi.org/10.1016/j.agee.2021.107778.

Number	Literature
10	**Huang, G.R., Zhang, Z.** (2007) The effect of tobacco rotation with different crops on the growth, development, and yield of tobacco. **6**:30-31. https://doi.org/10./j.cnki.52—1065/s.2007.06.014.
11	**Jia, W.Y., Liang, Y.L. Bai, C.H., Zhu, Y.L., Peng, Q., Lin, X.J., and Chen, C.** (2011). Effects of different proceding crops on growth physiology and quality of hot pepper. Agr. Res. Arid Areas.**29**:151-156.
12	**Chuan, YC., Zhang L.M., Jiao Y.G., Luo, L.F., Fang, Y.T., Liao, J.J., JI, S.G., Zhu, S.S., and Yang. M.** (2016). Control effects of tobacco and garlic rotation on tobacco black shank and a preliminary study on the inhibition mechanism. Acta Tab. Sin. **22**:55-62. https://doi.org/10.16472/j.chinatobacco.2016.200
13	**Tang, B., Zhang, X.Z., and Yang, X.B.** (2015). Effects of tobacco garlic crop rotation and intercropping on tobacco yield and rhizosphere soil phosphorus fractions. Chin. J. Appl. Ecol. **26**:1977-1984. https://pubmed.ncbi.nlm.nih.gov/26710622/.
14	**Miao, Q.S, Wang, D.S., Wei, Y.G., Huang, S.H, and Zhang, Y.Y.** (2021). Effects of intercropping and rotation on the growth of continuous crop eggplant and the incidence of *Verticillium* wilt. Chin. J. Veg. Sci. **34**:33-40. https://doi.org/10.16861/j.cnki.zggc.2021.0319.
15	**Jia, W.Y., Liang, Y.L. Bai, C.H., Zhu, Y.L., Peng, Q., Lin, X.J., Peng, Q., and Chen, C.** (2010). Effect of fore crops on growth yield and quality of hot pepper. J. Northwest A&F Univ. **38**:119-130 (Nat. Sci. Ed.). https://doi.org/10.13207/j.cnki.jnwafu.2010.05.024.
16	**Shi, G.H., Liang, Y.L., Yao, X.W., Zeng, R., and Mu, L.** (2013). Effects of Actinomycetes on yields and qualities of tomato and pepper under different fore Crops. Bull. Soil Water Conserv. 33:275-279. https://doi.org/10.13961/j.cnki.stbctb.2013.01.060.
17	**Hao, W.L., Liang, Y.L, Zhu, Y.L., Wu, X., Lin, X.J., and Luo, A.R.** (2011). Production efficiency and soil nutrient characteristics in food--vegetable rotation systems. Bull. Soil Water Conserv. **31**:46-51. https://doi.org/10.13961/j.cnki.stbctb.2011.02.031.
18	**Lee, H.U., Kim, C.H., and Nam, K.W.** (1991) Suppression of Phytophthora blight incidence of red pepper by cropping system. Plant Pathol. J. **7**: 147-152.
19	**Chen, Y.L., Lin, Z.Q., Tuo, Y.Y., Ding, Y.R., Li, H.L., and Wang, Y.** (2022). Effects

Number	Literature
	of tobacco garlic crop rotation on fungal community structure of susceptible tobacco soil. Southwest China J. Agri. Sci. **35**:972-980. https://doi.org/10.16213/j.cnki.scjas.2022.4.030.
20	**Yang, X.B., Li, T.X., Zhang, X.Z., Chen, D.R., Liang, Y.J., and Zhang, C.H**. (2016). Effects of tobacco garlic crop rotation and tobacco garlic crop intercropping on soil microbial groups in tobacco fields. Soils. **48**:698-704. https://doi.org/10.13758/j.cnki.tr.2016.04.012.
21	**Zhang, T., Chen, A.Q., Liu, J., Liu, H.B., Lei, B.K., Zhai, L., Zhang, D., and Wang, H.Y.** (2017). Cropping systems affect paddy soil organic carbon and total nitrogen stocks (in rice-garlic and rice-fava systems) in temperate region of southern China. Sci. Total Environ. **609**:1640-1649. https://doi.org/10.1016/j.scitotenv.2017.06.226.

Supplemental Table 8 The primers of three microorganisms for RT-primer

Species	Gene name	Sequence 5' to 3'
Penicillium allii	*GST*	TTTGAGCTTCGTTGCTTGGC
		CAGTCGGTCTCTCCTGCATC
	CYP628	TCGTTCGCCTCAGATCATCG
		TGCACATCAGTTTCCCTCGT
	FSP1	ATCAACCCATCGCCAACCTT
		ACTGGCTGAACTCCCTTTCG
	18S (reference gene)	GATGAAGAACGCAGCGAAAT
		TTGAAATGACGCTCGAACAG
Cylindrocarpon destructans	*ECHS1*	CAGCCTGGATGCTGTCTGTA
		CTTTTAGGGTCCCCCATCTC
	GST	AACCCAACTCGCCAAGTCAA
		CAGCCACTTCTCCAGGTGAG
	CHS1	TCATGTTCGATCCCTGGCAC
		TGCTGACCGAAGGGAGTTTC
	CYP53A1	TGTCTTACGGCCAAGACCAC
		ACAATTCTCAGCATCCCGCA
	18S (reference gene)	GATGAAGAACGCAGCGAAAT
		TTGAAATGACGCTCGAACAG
Phytophthora capsici	*SOD2*	TTCTCGCATCGCCTCTTCCG
		TTAGGAGCCAGGTTCGTCCA
	bglX	GTTCTCAATGGTGGCTTCGC
		ATCGGGTGTCCTCCGTTTTC
	ABC1	CAACACTCGCACACACAACA
		ATTTCACGCTTGGAGGGTCC
	CDC6	ATTGAACGCACAACAACCCG
		CGCGTGCTTGATAATGCGAA
	Tublin B (reference gene)	CCAGCTTCAGCCTTCACTTC
		CATCCCAATCCTGATCCTGT

Supplemental Table 9 The primers of pepper and garlic for RT-qPCR

Species	Gene name	Sequence 5' to 3'
Pepper	*PAL*	CAACAGCAACATCACCCCATGTTTGC
		GCTGCAACTCGAAAAATCCACCAC
	PR1c	AAATGCAGCACGTTCAGCA
		CGTTCCCACTTCCCCAAA
	Actin-1 (reference gene)	GTCCTCTTCCAACCATCCAT
		TACTTTCTCTCTGGTGGTGC
Garlic	*PAL*	GAAGTCGTTCACCAGCTCGGAGAAC
		GACGTGTCAAGGAAAAAGCCGTG
	PR1c	GGCGGTCCTTATGGTGAAA
		GCCAGGGTCACATGTGTTA
	GAPDH (reference gene)	AGGCTGGTGCTGATTACG
		AGGTCTGAAGTGTATGAAGTATGG

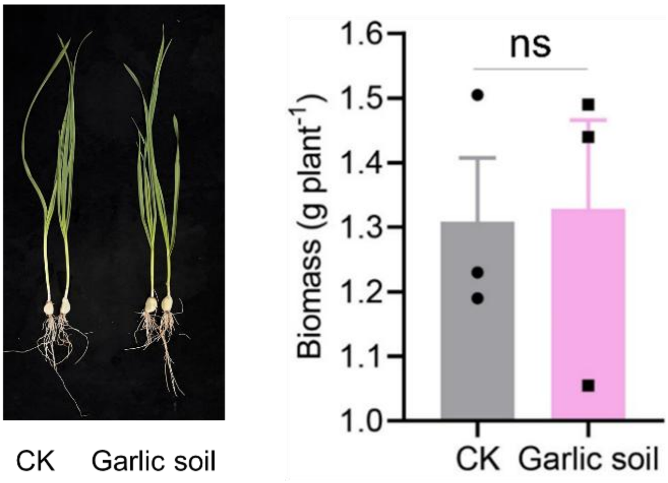

Supplemental Figure 1 Effect of garlic-conditioned soil on the growth of garlic.

CK soil represents the blank treatment without garlic planting, and Garlic soil represents the garlic-conditioned soil treatment. Data are expressed as mean ± standard error (*n* = *3*). Data are expressed as mean ± SE. ns represents no significant difference. (Independent sample t test, $p < 0.05$)

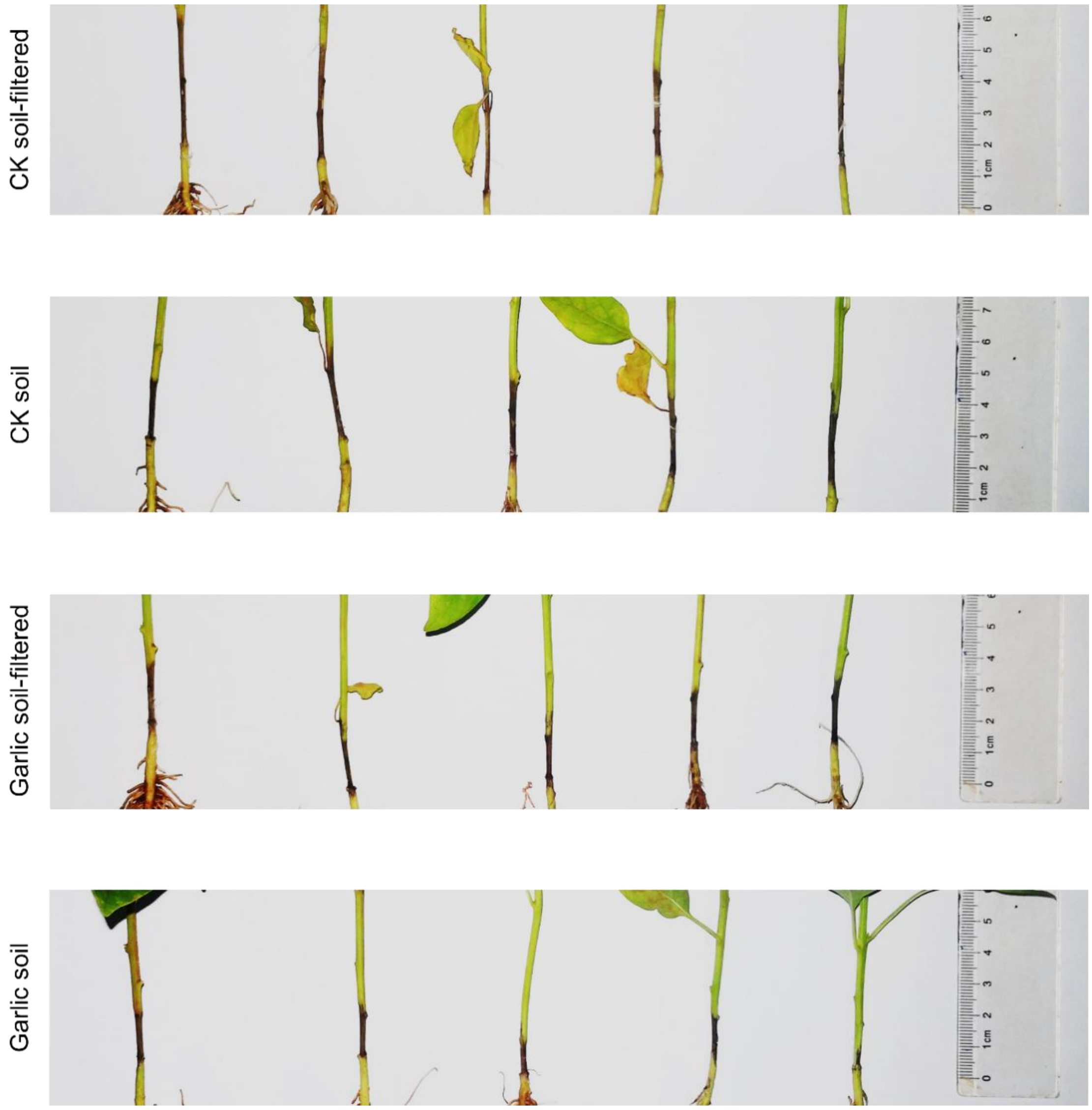

Supplemental Figure 2 Effects of garlic soil suspension and control soil suspension on the expansion of pepper blight lesions.

CK soil represents the blank treatment without garlic planting, and garlic soil represents the garlic-conditioned soil treatment. The soil suspensions were filtered through a 0.22 μm filter to remove microorganisms.

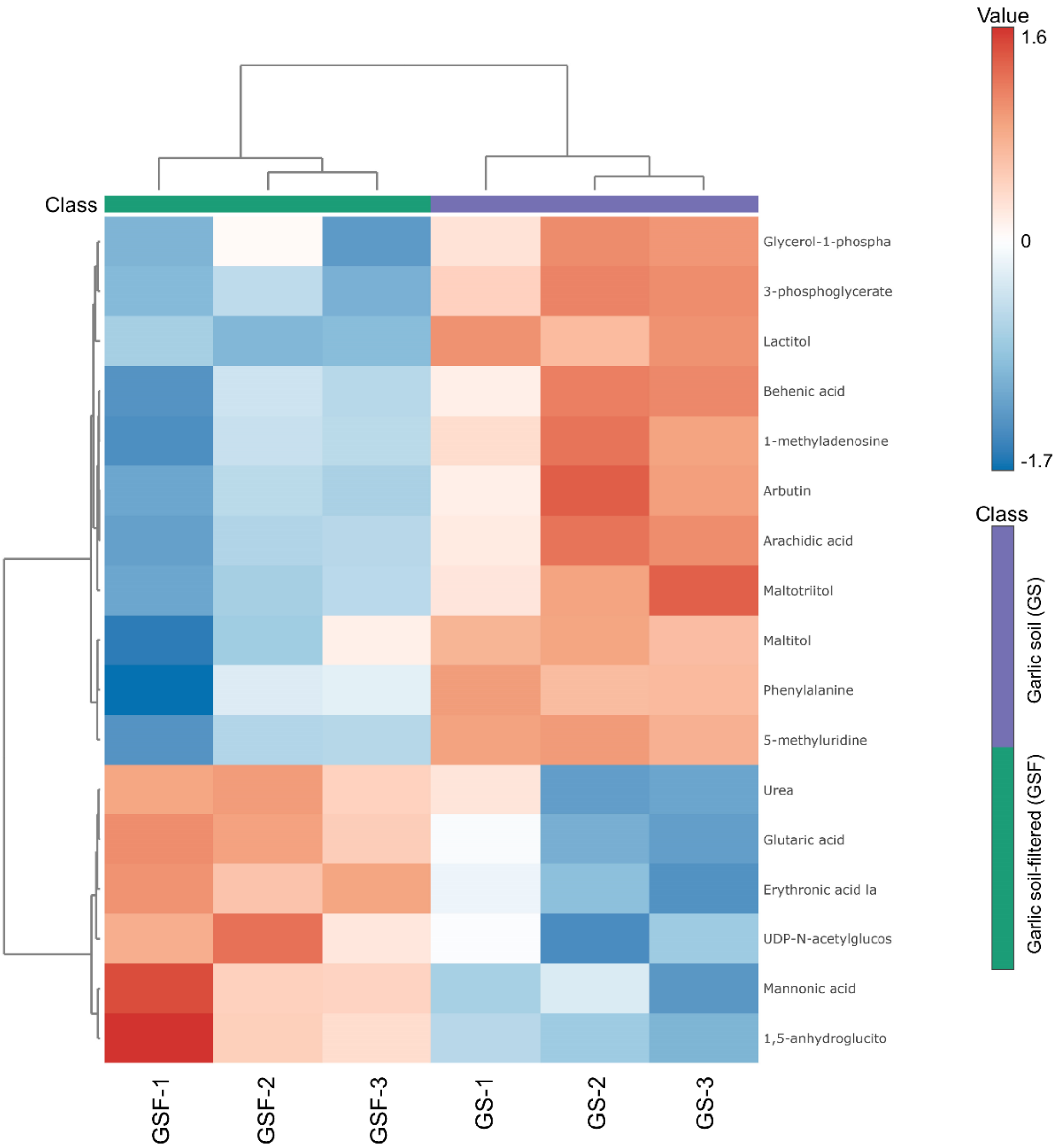

Supplemental Figure 3 Heat map of differential metabolites in the shoots of pepper after exposured to filtered and unfiltered garlic soil suspension.

GSF represents for filtered garlic-conditioned soil suspension treatment, and GS represents for unfiltered garlic-conditioned soil suspension treatment.

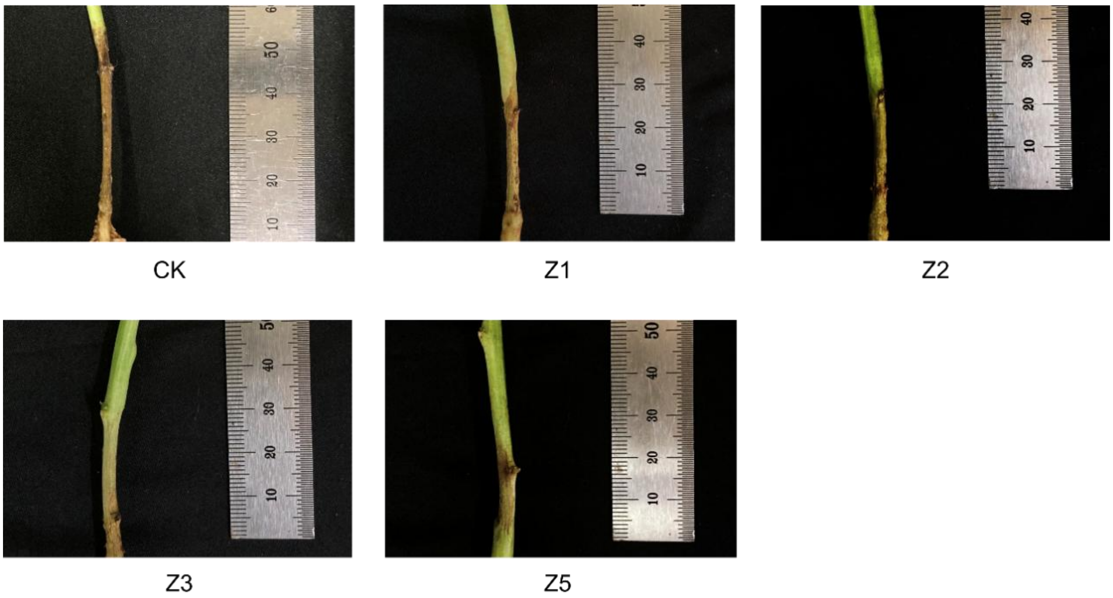

Supplemental Figure 4 Effects of soil suspension from the conditioned **soil with different garlic densities on the lesion expansion of pepper *Phytophthora* blight on stem.**

Z1, Z2, Z3, and Z5 represent 1, 2, 3, and 5 garlic plants per pot, respectively. CK represents control group

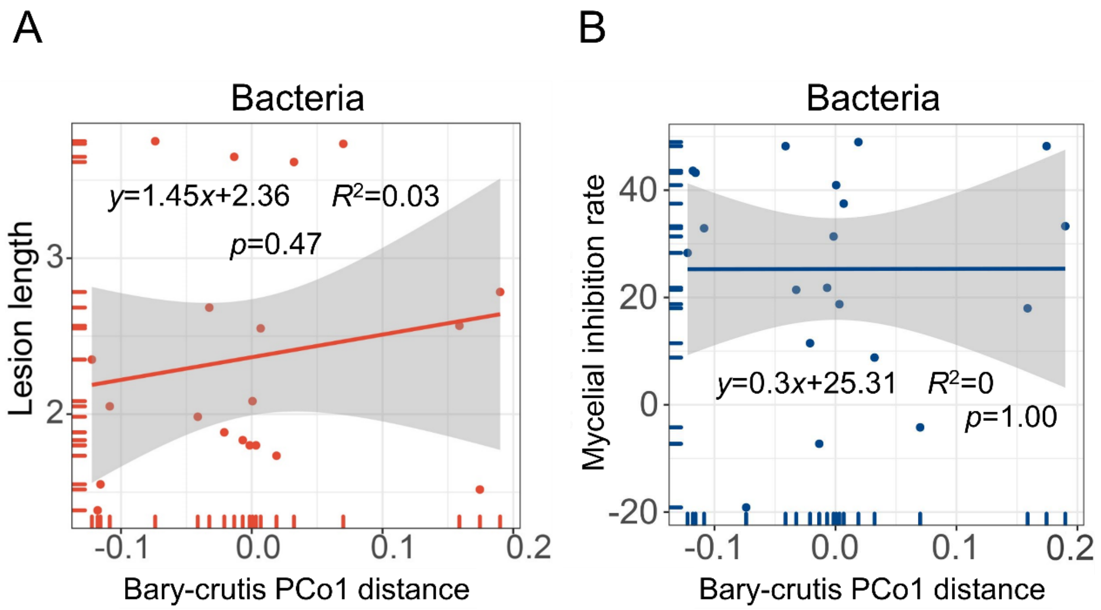

Supplemental Figure 5 Correlation analysis between bacterial community structure (PCo1 axis) at the genus level and lesion length (A) or mycelial inhibition rate (B).

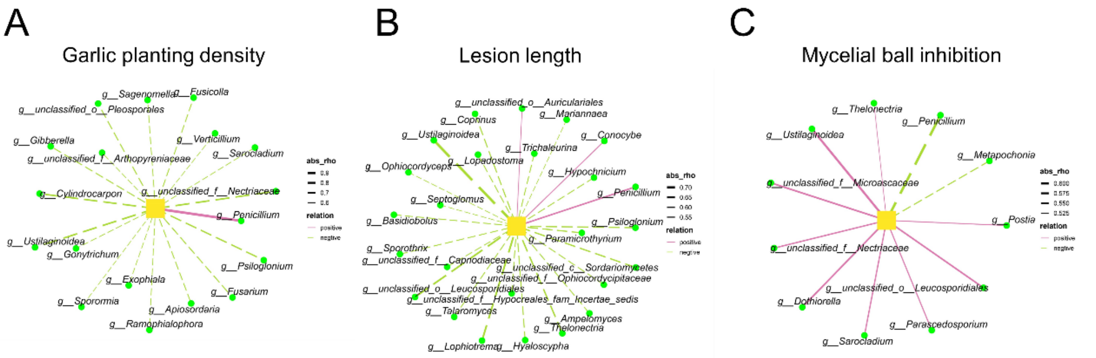

Supplemental Figure 6 Correlation analysis between garlic-conditioned soil fungi and planting density, pepper blight lesion length and *P. capsici* mycelial inhibition.

(A) Correlation analysis between garlic-conditioned soil microorganisms and planting density.

(B) Correlation analysis of garlic-conditioned soil microorganisms and *P. capsici* mycelial inhibition.

(C) Correlation analysis between garlic-conditioned soil microorganisms and pepper *Phytophthora* blight lesion length. The green dashed line represents a negative correlation, and the pink solid line represents a positive correlation

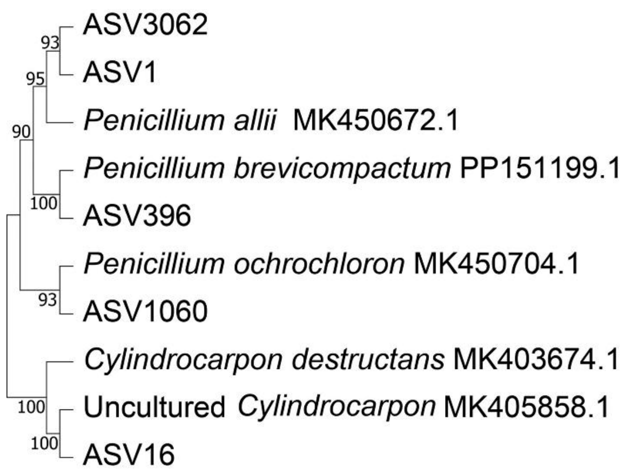

Supplemental Figure 7 Evolutionary tree of *Penicillium* species and ASV level based on ITS.

Bootstrap values based on 1000 replications are shown as percentages at each branch.

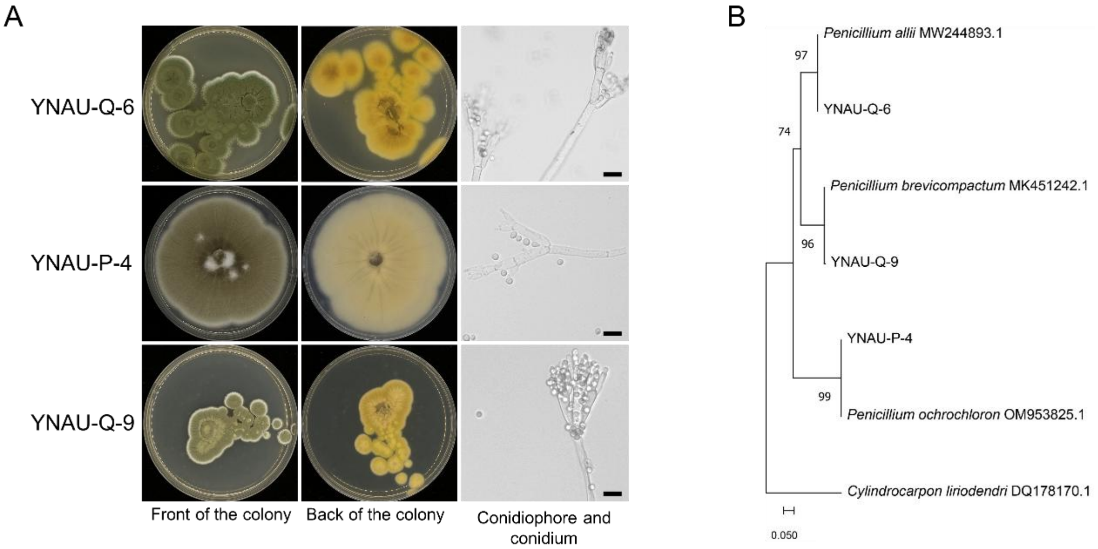

Supplemental Figure 8 Morphological and molecular biological identification of isolated *Penicillium*

(A) Morphological identification of *Penicillium*.

(B) Evolutionary tree of *Penicillium* based on β-tubulin.

Bootstrap values based on 1000 replications are shown as percentages at each branch. Bar = 10 μm.

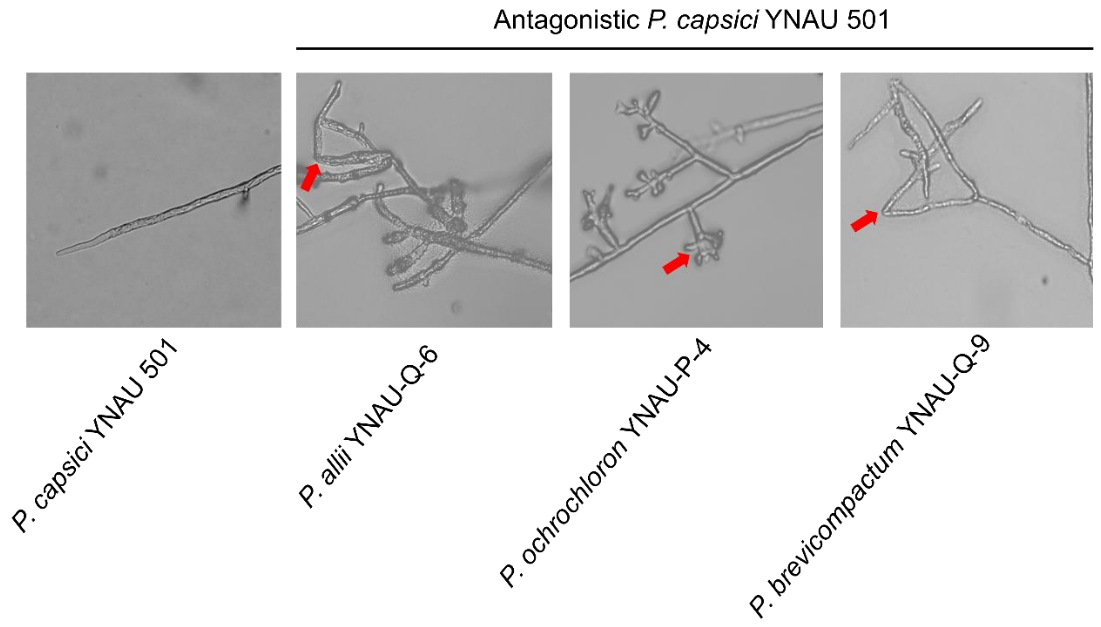

Supplemental Figure 9 Effect of isolated *Penicillium* species on mycelium morphology of *P. capsici.*

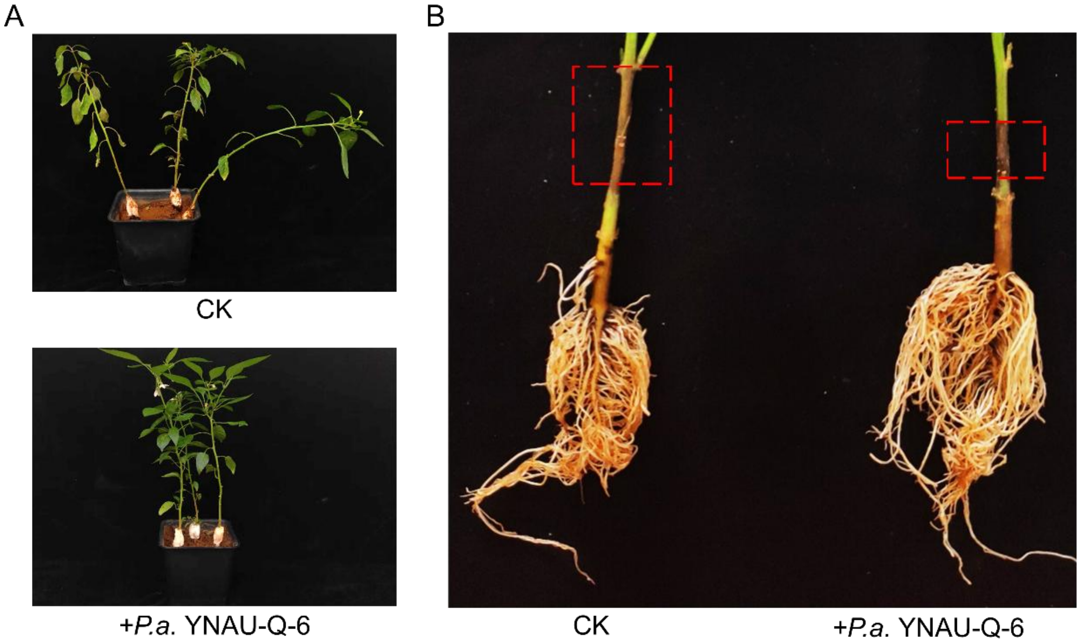

Supplemental Figure 10 Effects of *Penicillium allii* YNAU-Q-6 on pepper *Phytophthora* blight lesion length.

(A) Symptoms of pepper blight under YNAU-Q-6 treatment.

(B) Effects of YNAU-Q-6 on pepper blight lesion length.

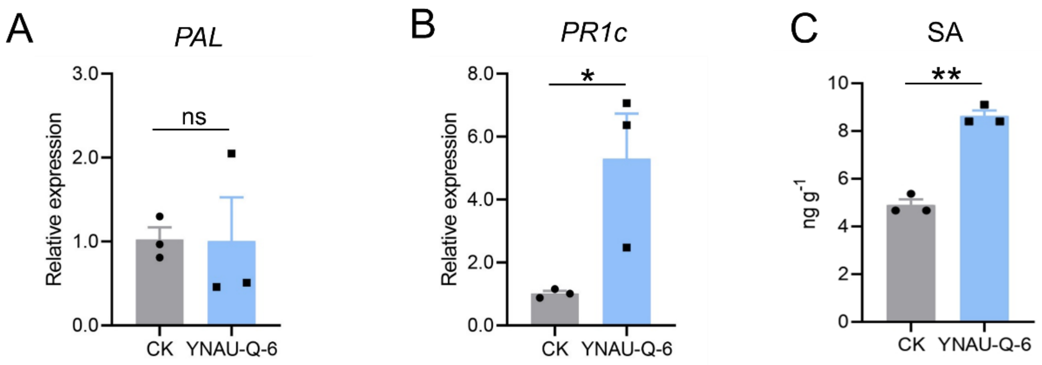

Supplemental Figure 11 The relative expression of resistance genes and salicylic acid contents in pepper shoots induced by *P. allii* YNAU-Q-6.

(A) The relative expression of phenylalanine ammonia-lyase (*PAL*) in pepper shoots.

(B) The relative expression of pathogenesis-related protein 1c (*PR1c*) in pepper shoots.

(C) The salicylic acid (SA) contents in pepper shoots.

Data are expressed as mean ± standard error. An independent sample *t*-test was used for data significance analysis. ** indicates $p < 0.01$, * indicates $p < 0.05$, ns means no significance.

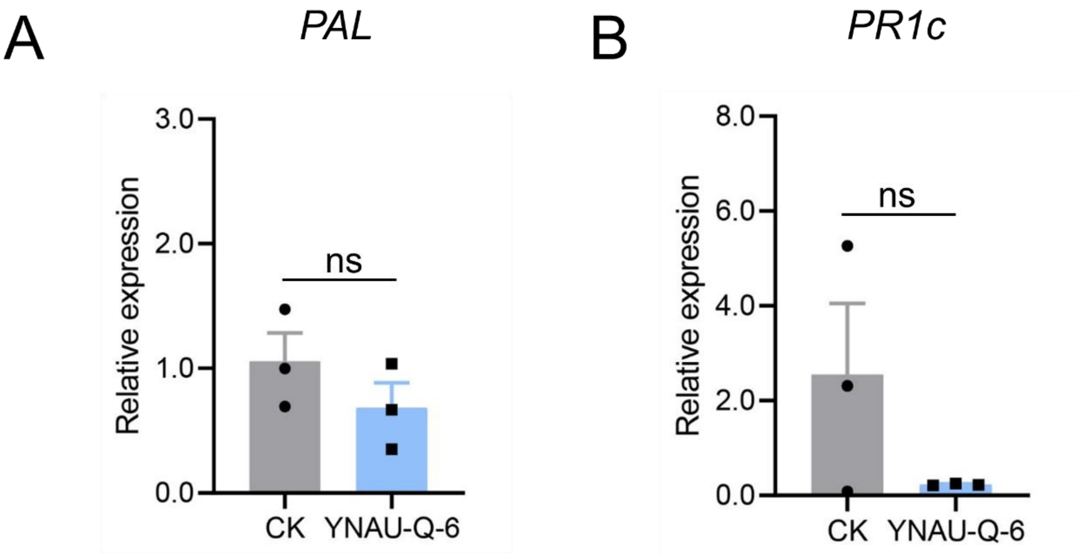

Supplemental Figure 12 The relative expression of resistance genes in garlic seedlings induced by *P. allii* YNAU-Q-6.

(A) The relative expression of phenylalanine ammonia-lyase (*PAL*) in garlic seedlings.

(B) The relative expression of pathogenesis-related protein 1c (*PR1c*) in garlic seedlings.

Data are expressed as mean ± standard error. An independent sample *t*-test was used for data significance analysis. ns means no significance.

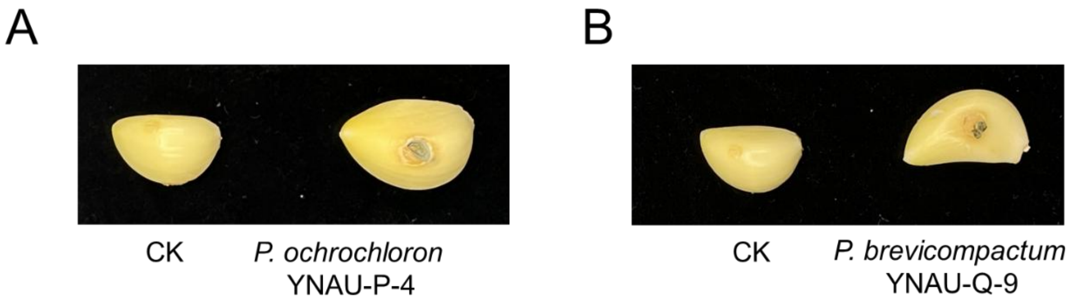

Supplemental Figure 13 Effects of *P. ochrochloron* YNAU-P-4 (A) and *P. brevicompactum* YNAU-Q-9 (B) on garlic cloves.

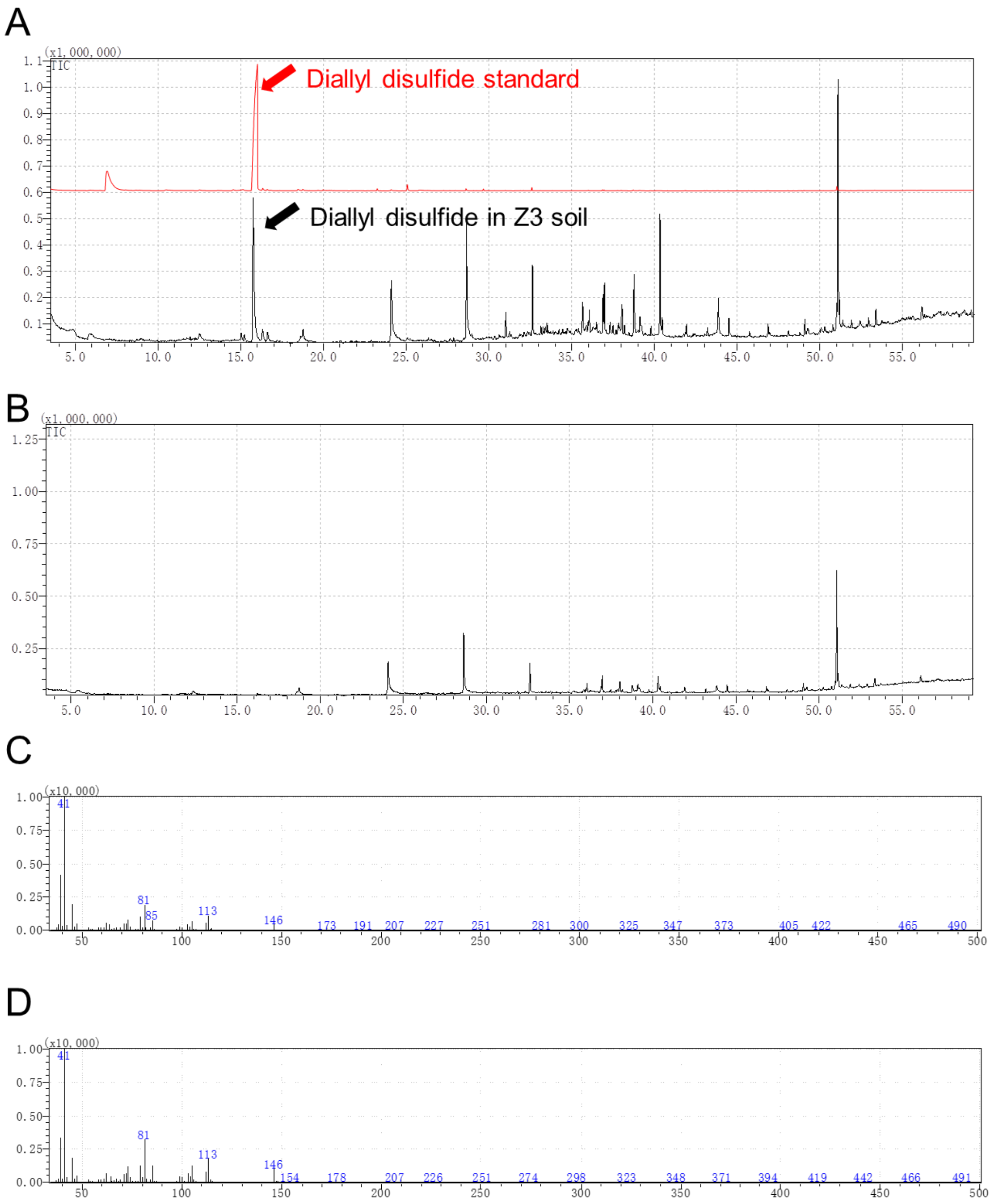

Supplemental Figure 14 Volatile compounds in pot-grown garlic-conditioned soil (Z3 treatment) by GC-MS.

(A) Total ion current (TIC) chromatograms of garlic-conditioned soil.

(B) Total ion current (TIC) chromatograms of CK soil.

(C) The mass spectrum of diallyl disulfide in garlic-conditioned soil.

(D) The mass spectrum of standard diallyl disulfide.

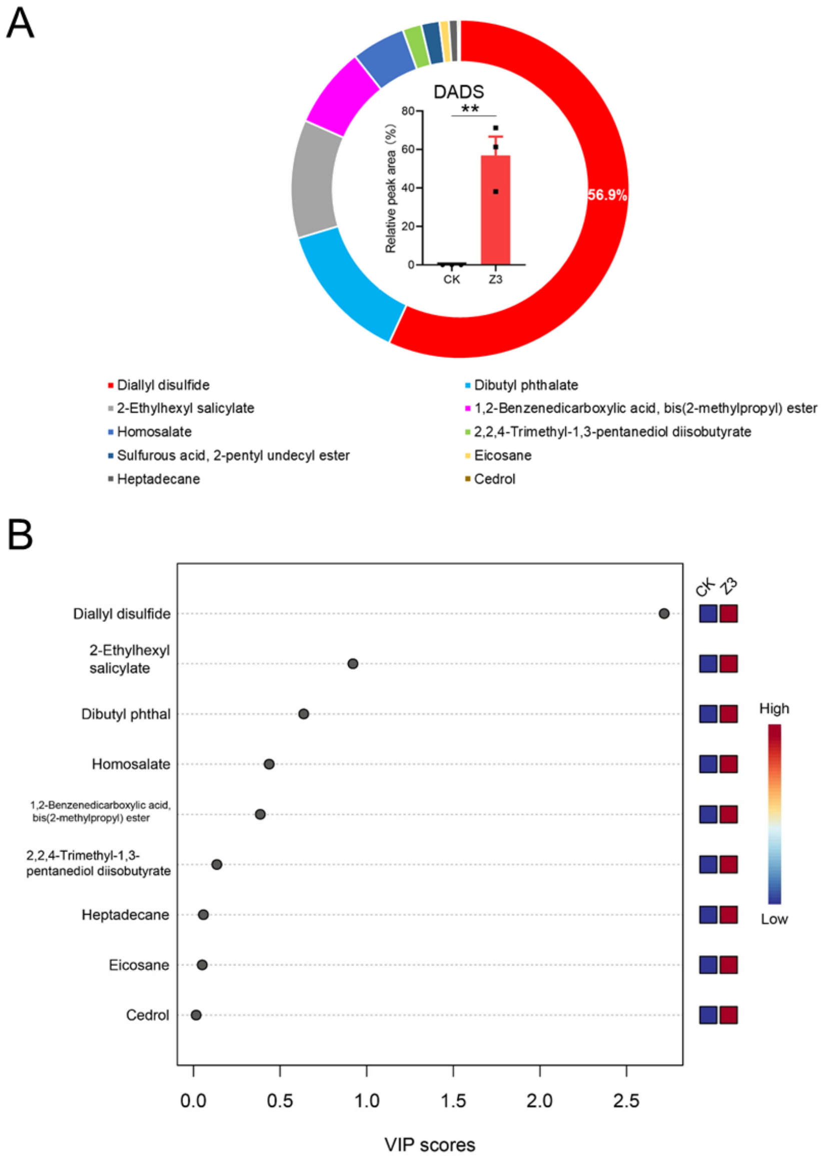

Supplemental Figure 15 Detection of volatile compounds from garlic-conditioned soil in the pot

(A) Relative peak area ratio of volatile compounds in Z3 treatment.

(B) Variable importance in projection analysis (VIP) of volatile compounds between CK and Z3 treatment.

Data are expressed as mean ± standard error. An independent sample *t*-test was used for data significance analysis. ** indicates $p < 0.01$.

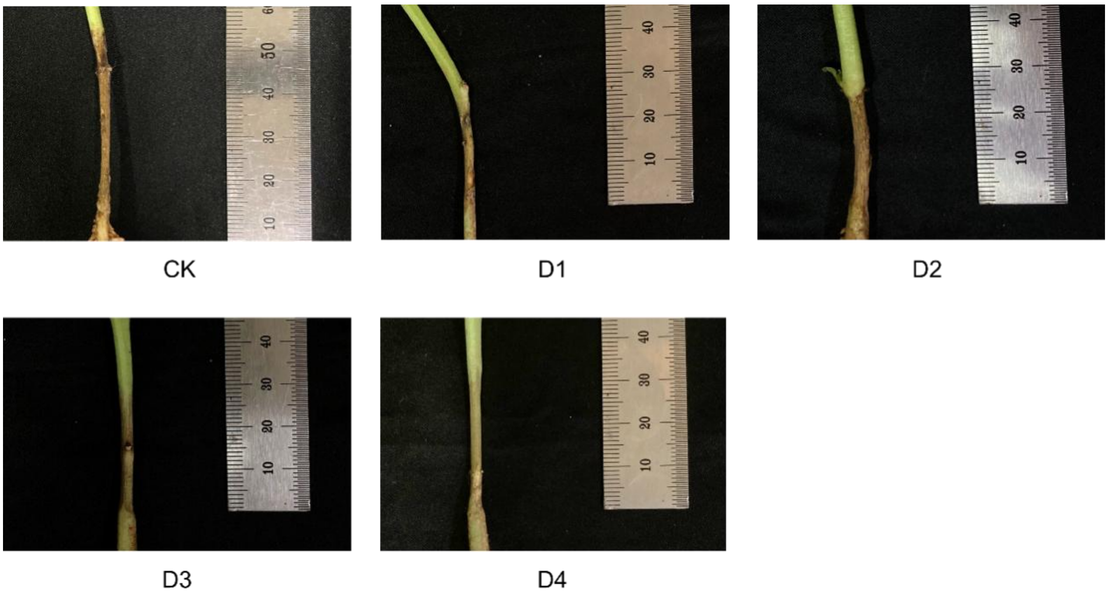

Supplemental Figure 16 Effects of soil microbiome shaped by exogenous DADS with different concentrations on pepper blight expansion.

D1, D2, D3, and D4 represent soil DADS concentrations of 13.7, 27.3, 54.7, and 109.4 µmol kg^{-1}, respectively. CK represents control group.

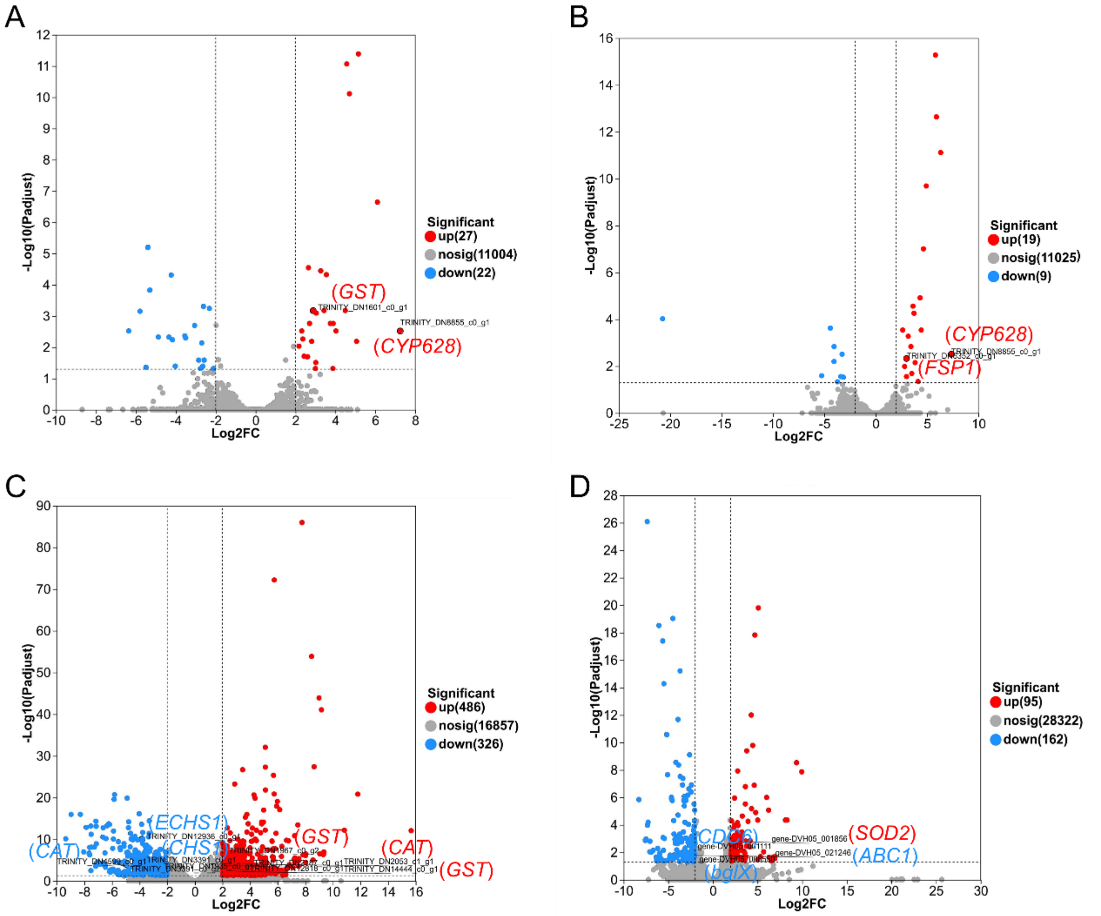

Supplementary Figure 17 Volcano plot of differentially expressed genes (DEGs) from different microorganisms after DADS treatment

(A) DEGs of ***P. allii*** YNAU-Q-6 treated with 13.7 μM DADS.

(B) DEGs of ***P. allii*** YNAU-Q-6 treated with 273.5 μM DADS.

(C) DEGs of ***C. destructans*** YNAU-RS-6 treated with 273.5 μM DADS.

(D) DEGs of ***P. capsici*** YNAU-501 treated with 683.7 μM DADS.

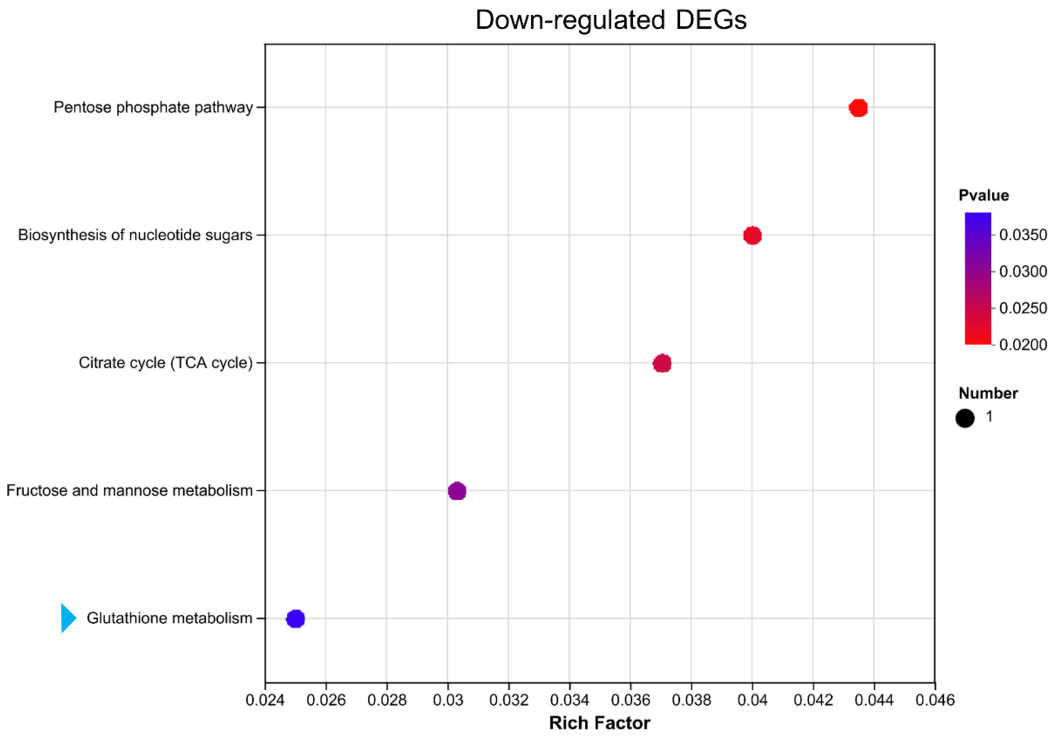

Supplemental Figure 18 KEGG pathway enrichment analysis of *P. allii* YNAU-Q-6 after 13.7 µM DADS treatment.

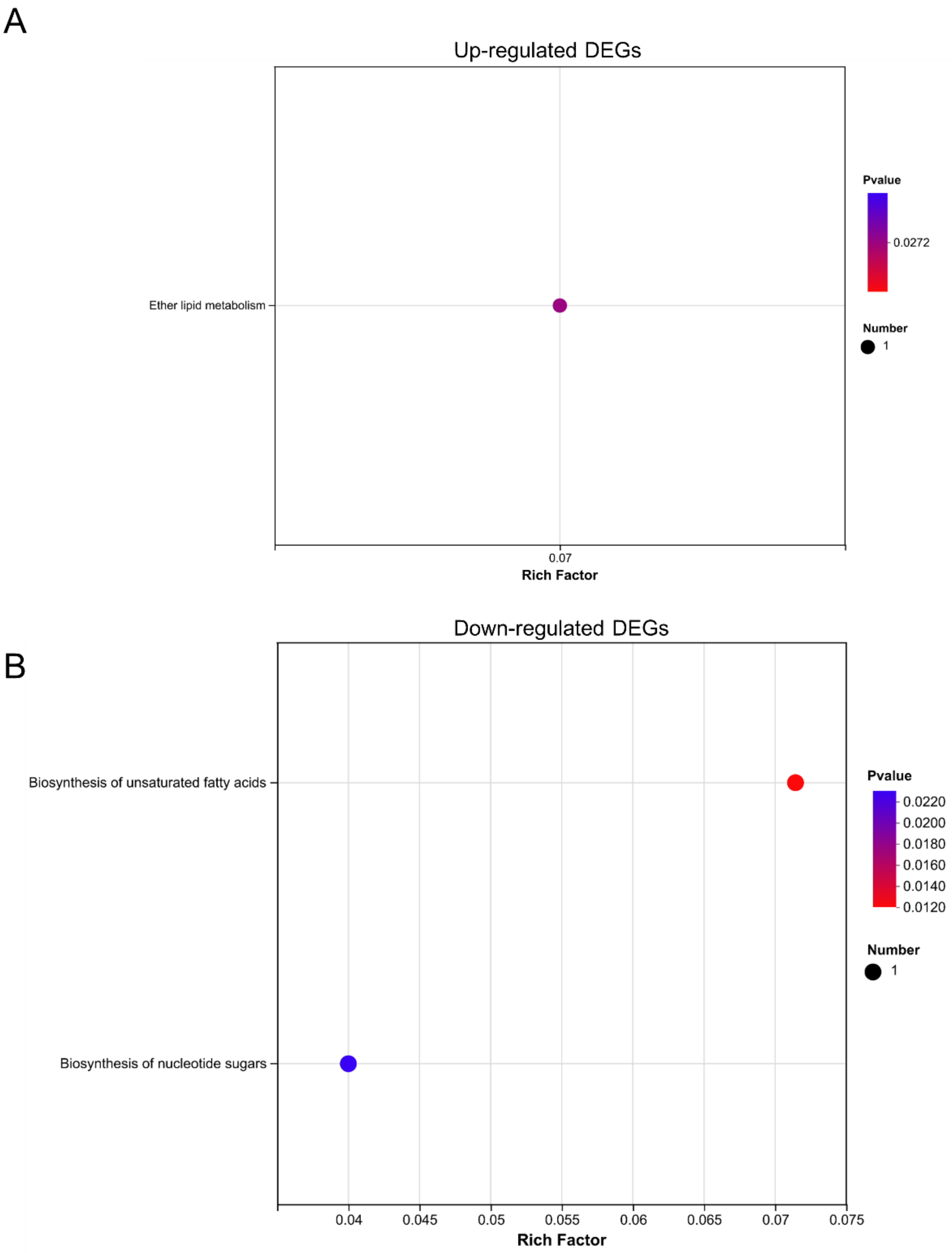

Supplemental Figure 19 KEGG pathway enrichment analysis of *P. allii* YNAU-Q-6 after 273.5 µM DADS treatment.

(A) KEGG pathway enrichment analysis of up-regulated DEGs.

(B) KEGG pathway enrichment analysis of down-regulated DEGs.

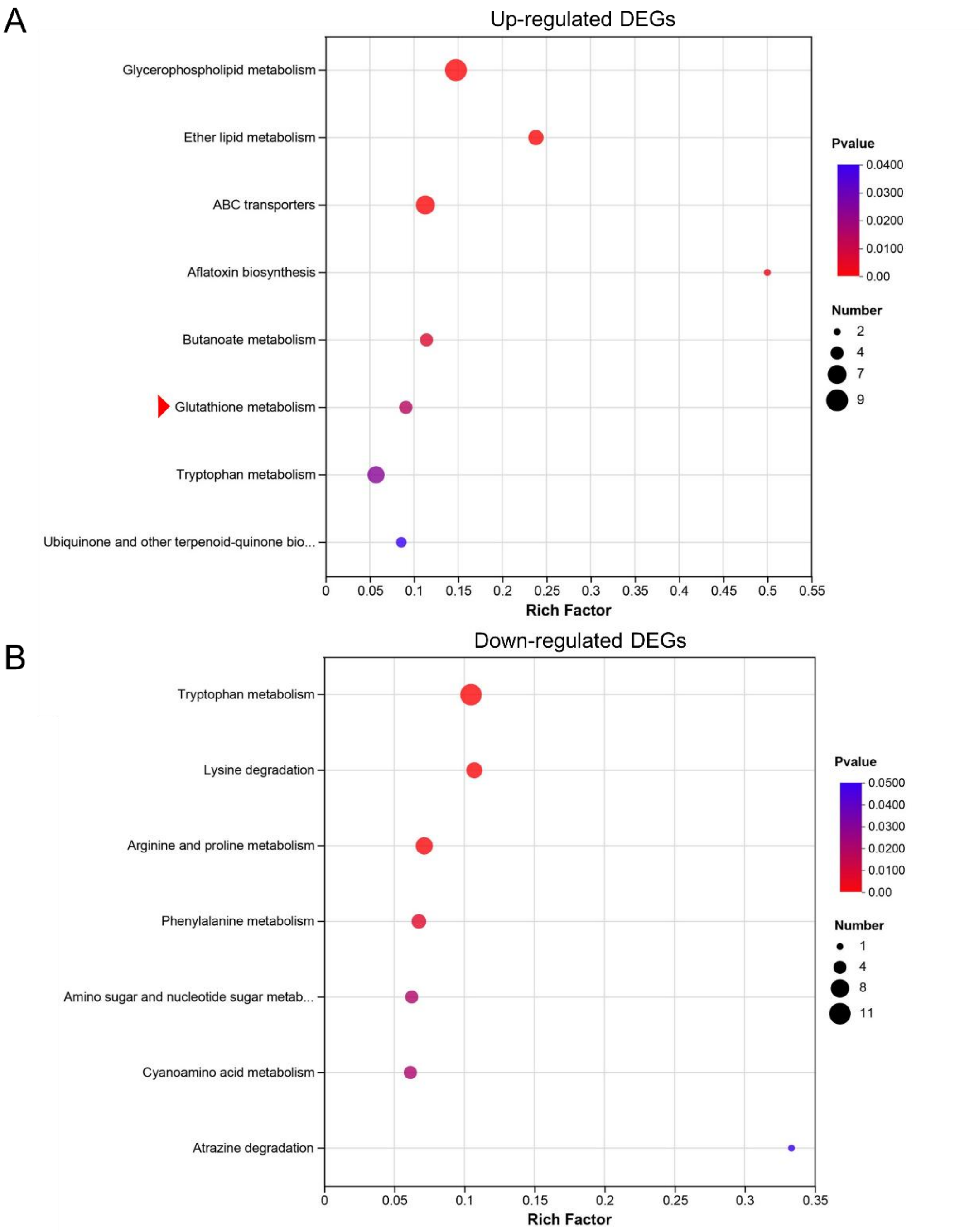

Supplemental Figure 20 KEGG pathway enrichment analysis of *C. destructans* YNAU-RS-6 after 273.5 μM DADS treatment.

(A) KEGG pathway enrichment analysis of up-regulated DEGs.

(B) KEGG pathway enrichment analysis of down-regulated DEGs.

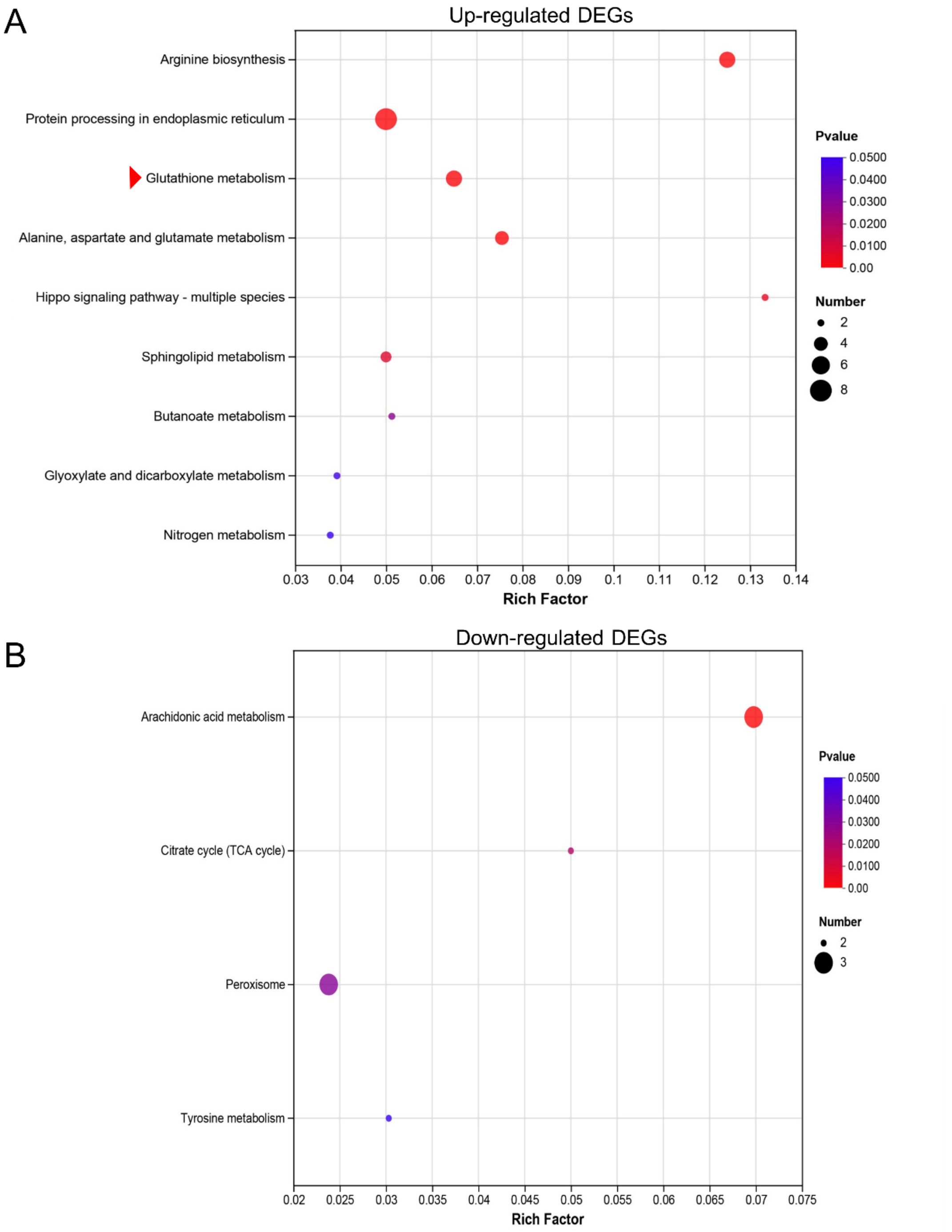

Supplementary Figure 21 KEGG pathway enrichment analysis of *P. capsici* YNAU-501 after 683.7 μM DADS treatment.

(A) KEGG pathway enrichment analysis of up-regulated DEGs.

(B) KEGG pathway enrichment analysis of down-regulated DEGs.

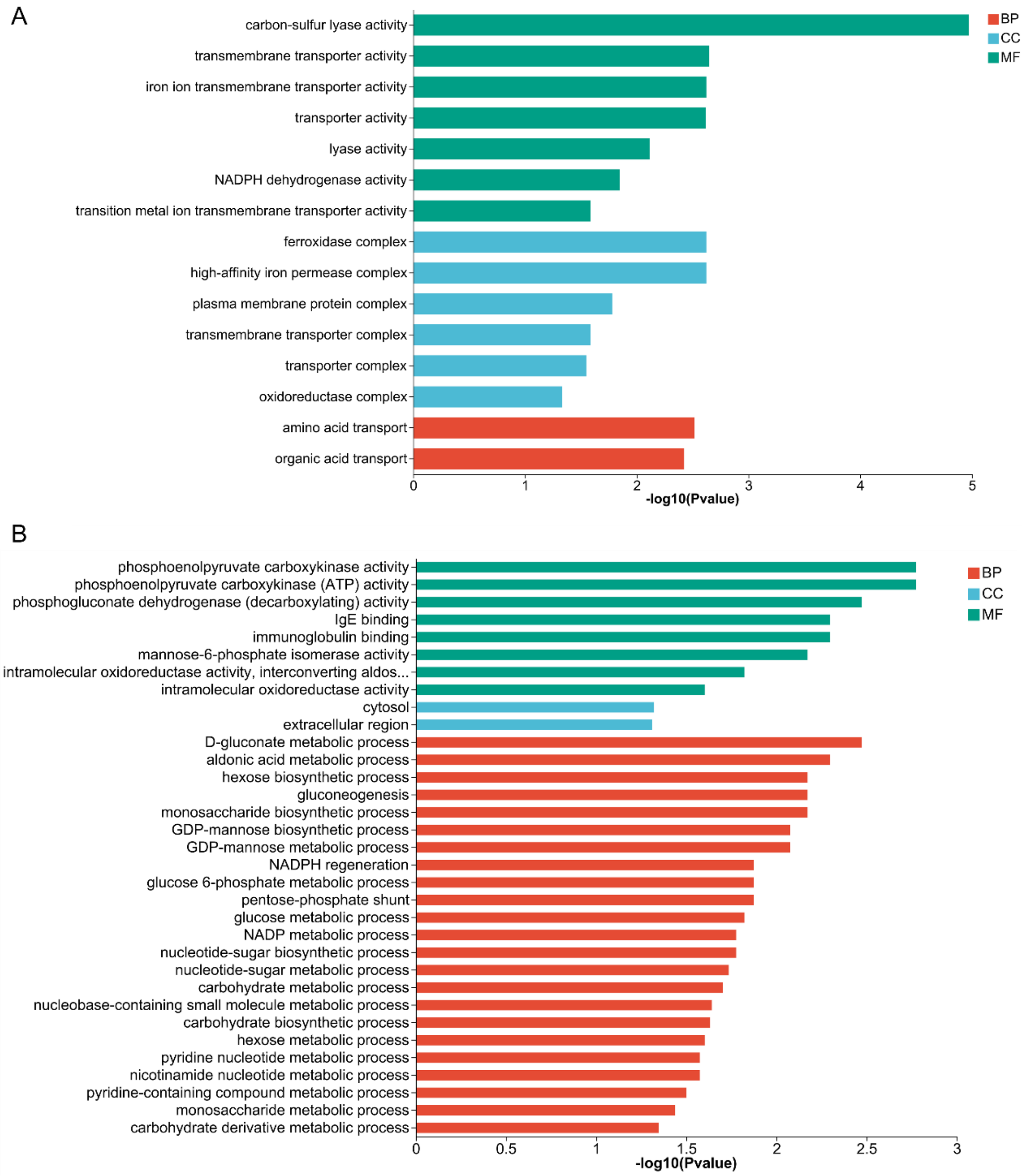

Supplemental Figure 22 GO pathway enrichment analysis of *P. allii* YNAU-Q-6 after 13.7 μM DADS treatment.

(A) GO pathway enrichment analysis of up-regulated DEGs.

(B) GO pathway enrichment analysis of down-regulated DEGs.

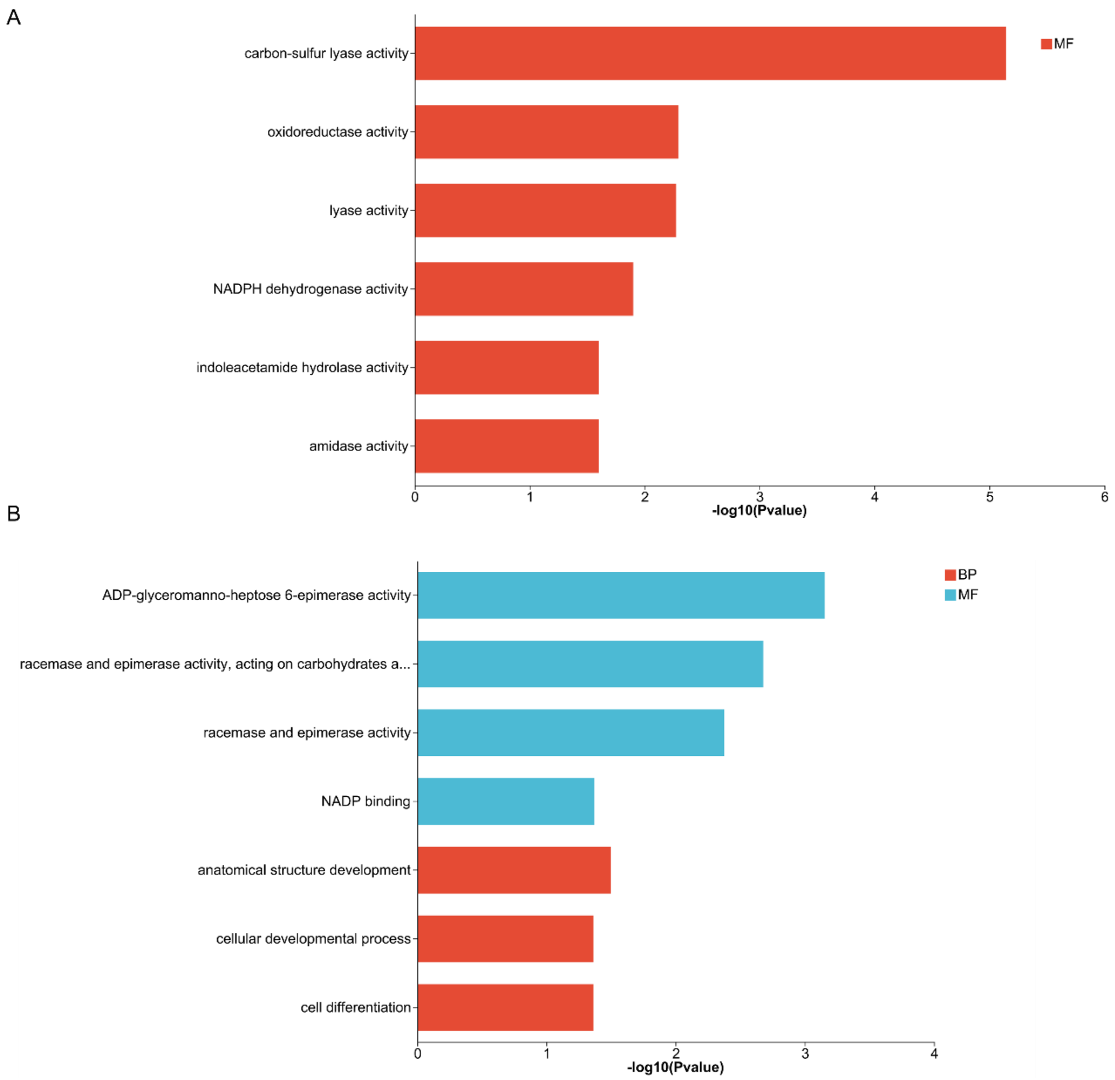

Supplemental Figure 23 GO pathway enrichment analysis of *P. allii* YNAU-Q-6 after 273.5 µM DADS treatment.

(A) GO pathway enrichment analysis of up-regulated DEGs.

(B) GO pathway enrichment analysis of down-regulated DEGs.

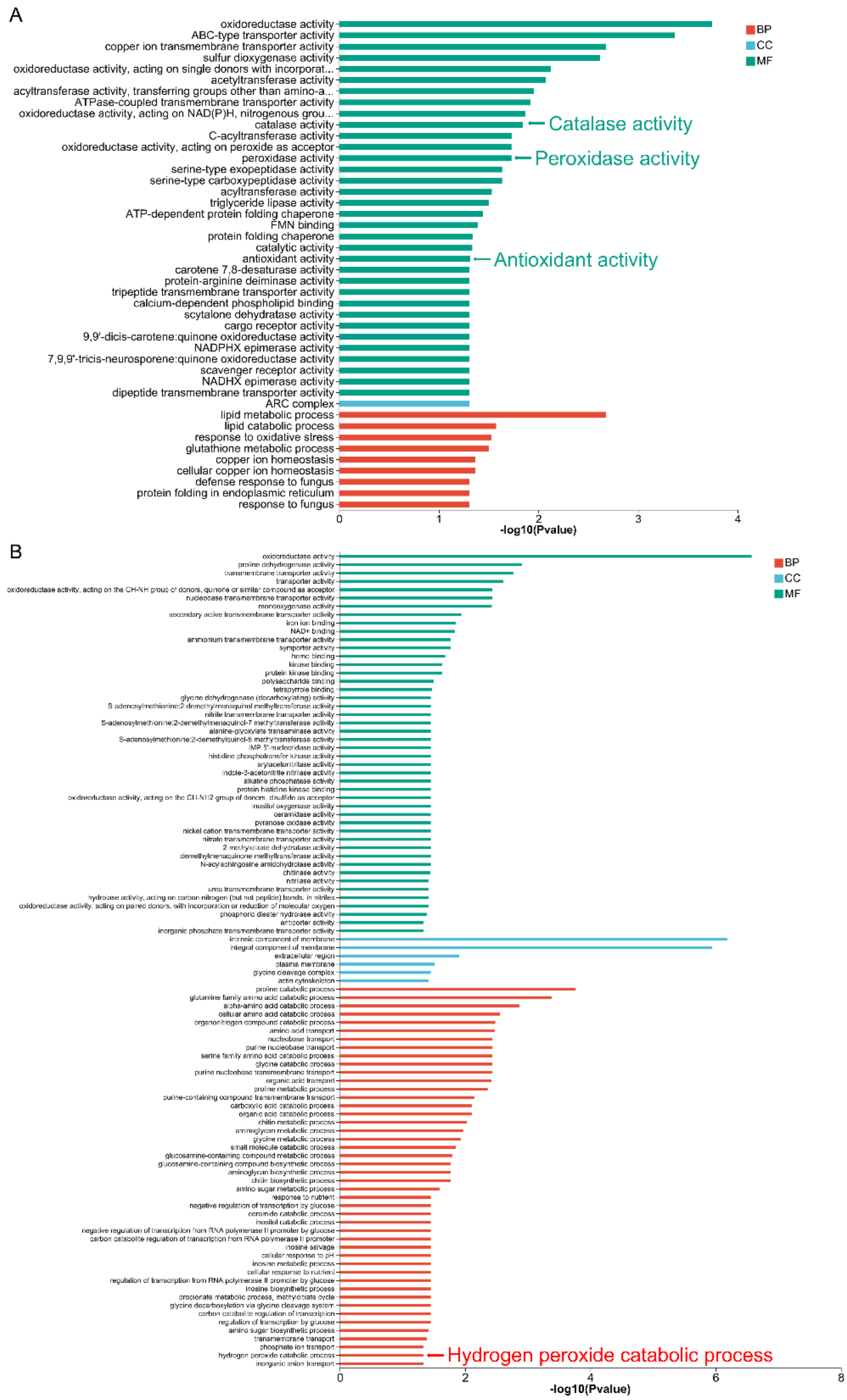

Supplemental Figure 24 GO pathway enrichment analysis of *C. destructans* YNAU-RS-6 after 273.5 µM DADS treatment.

(A) GO pathway enrichment analysis of up-regulated DEGs.

(B) GO pathway enrichment analysis of down-regulated DEGs.

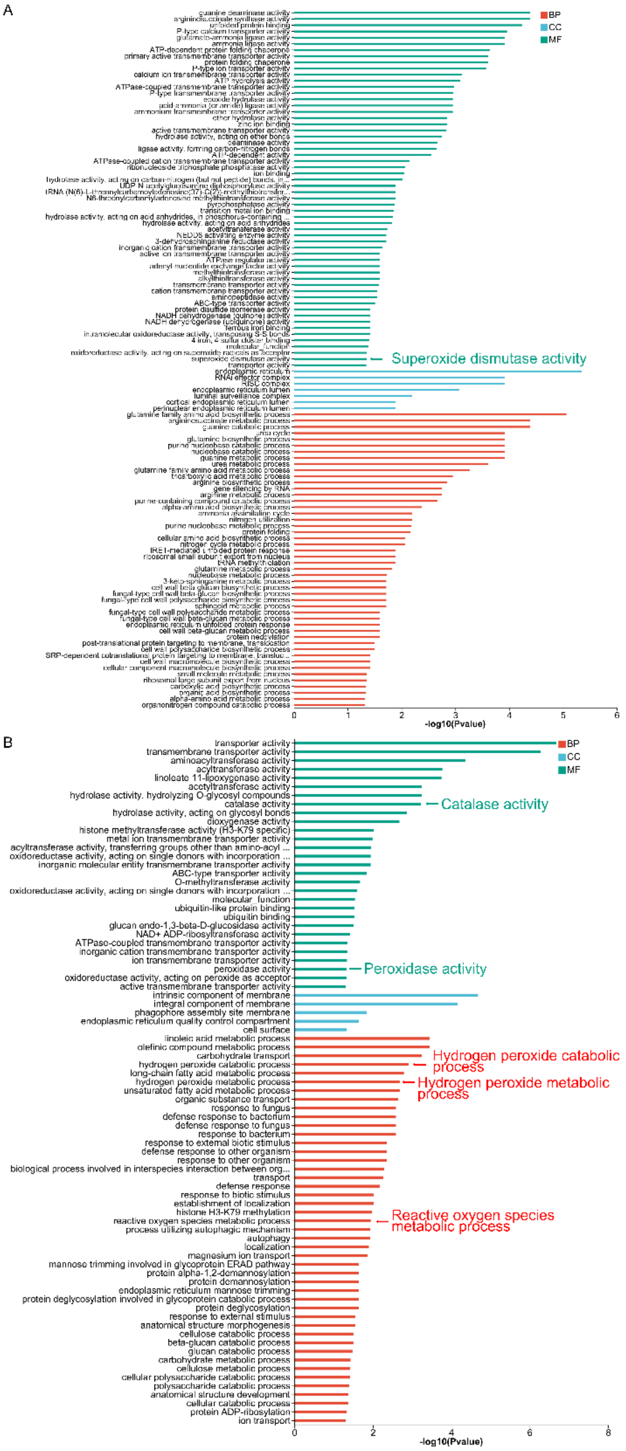

Supplementary Figure 25 GO pathway enrichment analysis of *P. capsici* YNAU-501 683.7 µM after DADS treatment.

(A) KEGG pathway enrichment analysis of up-regulated DEGs.

(B) KEGG pathway enrichment analysis of down-regulated DEGs.

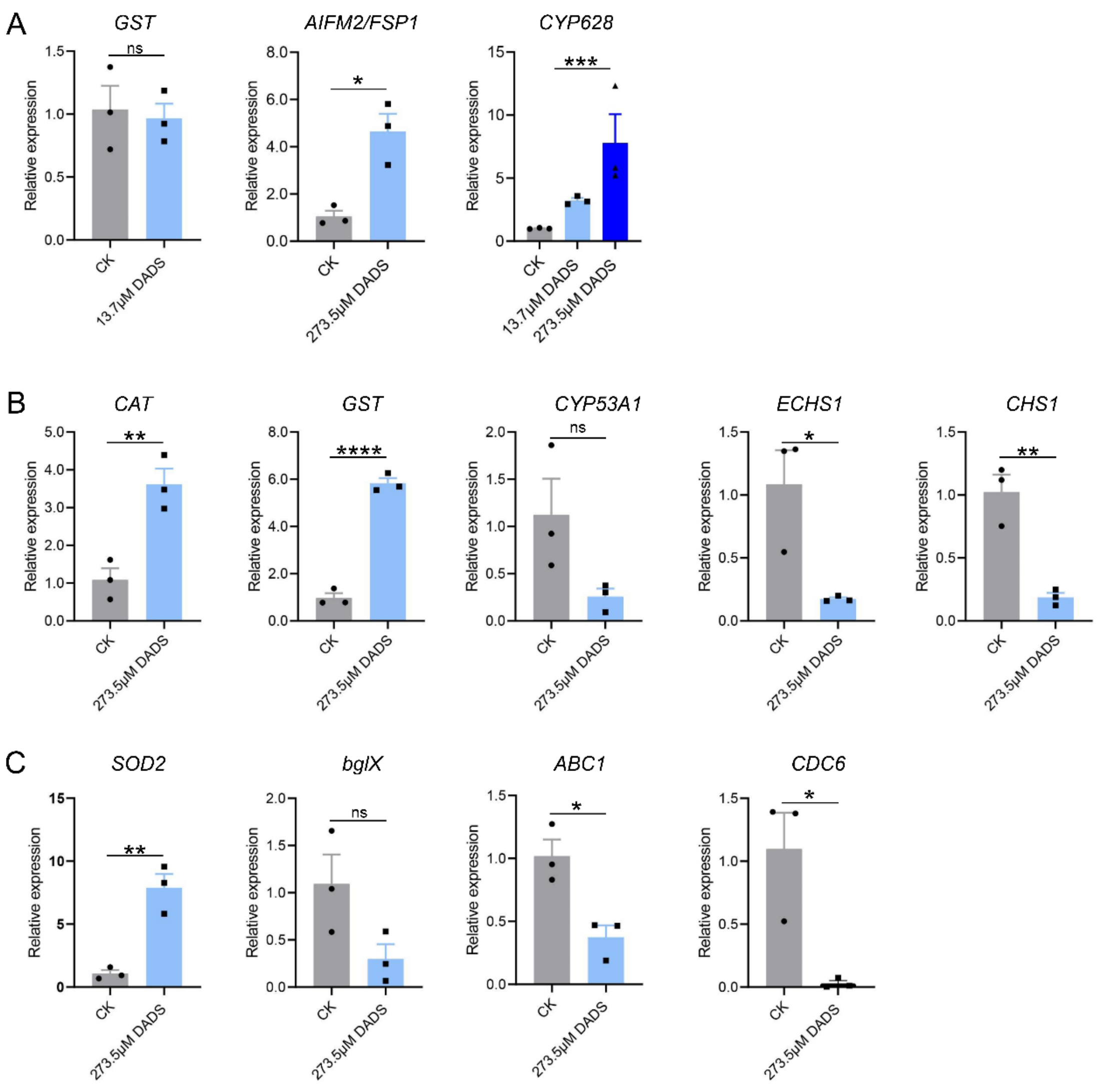

Supplemental Figure 26 Validation of differentially expressed genes using RT-qPCR.

(A) RT-qPCR detection of DEGs in *P. allii* YNAU-Q-6.

(B) RT-qPCR detection of DEGs in *C. destructans* YNAU-RS-6.

(C) RT-qPCR detection of DEGs in *P. capsici* YNAU-501.

Data are expressed as mean ± standard error. An independent sample *t*-test was used for data significance analysis. * indicates $p < 0.05$, ** indicates $p < 0.01$, *** indicates $p < 0.001$, **** indicates $p < 0.0001$, ns means no significance.

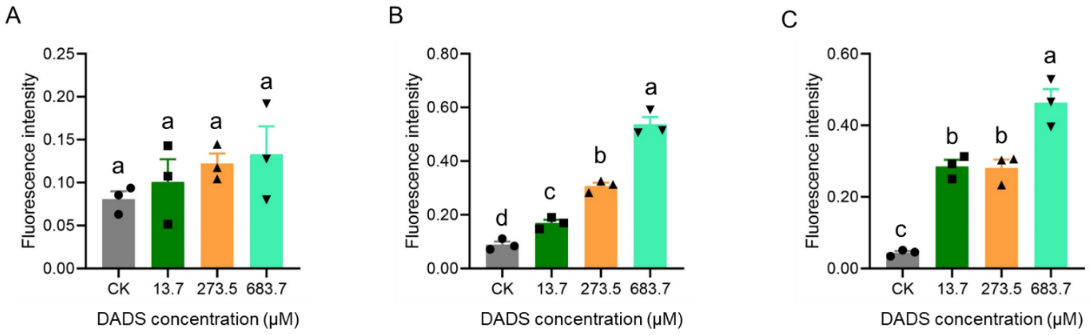

Supplemental Figure 27 ROS quantitative detection of three isolates induced by DADS

(A) Fluorescence intensity detection in *P. allii* YNAU-Q-6.

(B) Fluorescence intensity detection in *C. destructans* YNAU-RS-6.

(C) Fluorescence intensity detection in *P. capsici* YNAU-501.

Data are expressed as mean ± standard error. Different lowercase letters indicate significant differences between treatments ($p < 0.05$, according to ANOVA followed by Duncan's multiple range test).

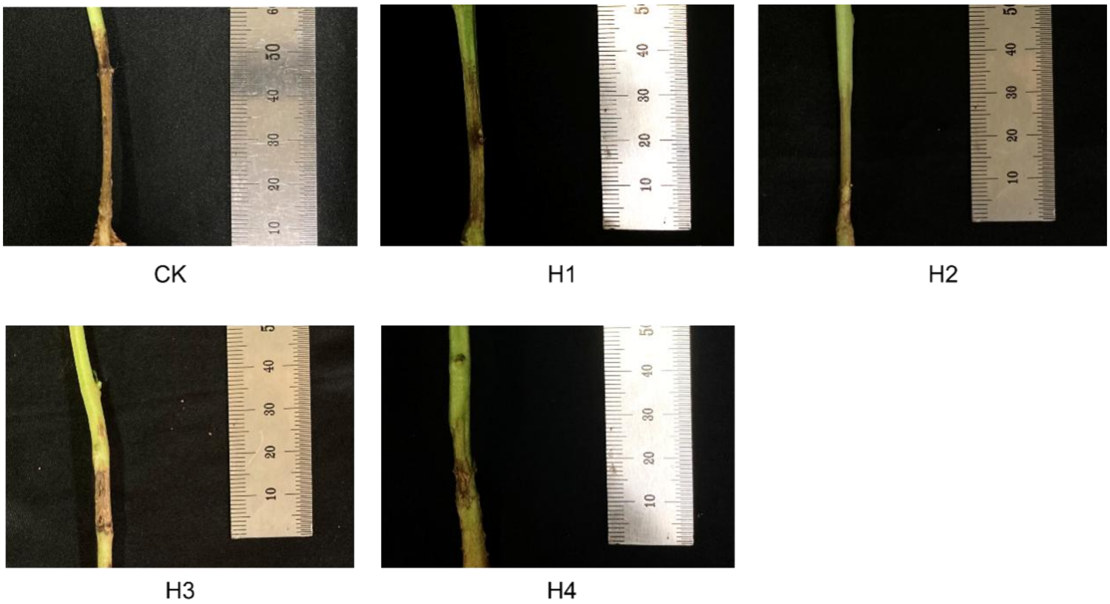

Supplemental Figure 28 Effects of soil microbiome shaped by exogenous H_2O_2 with different concentrations on pepper blight expansion.

H1, H2, H3, and H4 represent soil H_2O_2 concentrations of 85.9, 171.7, 343.4, and 686.9 μmol kg^{-1}, respectively. CK represents control group.

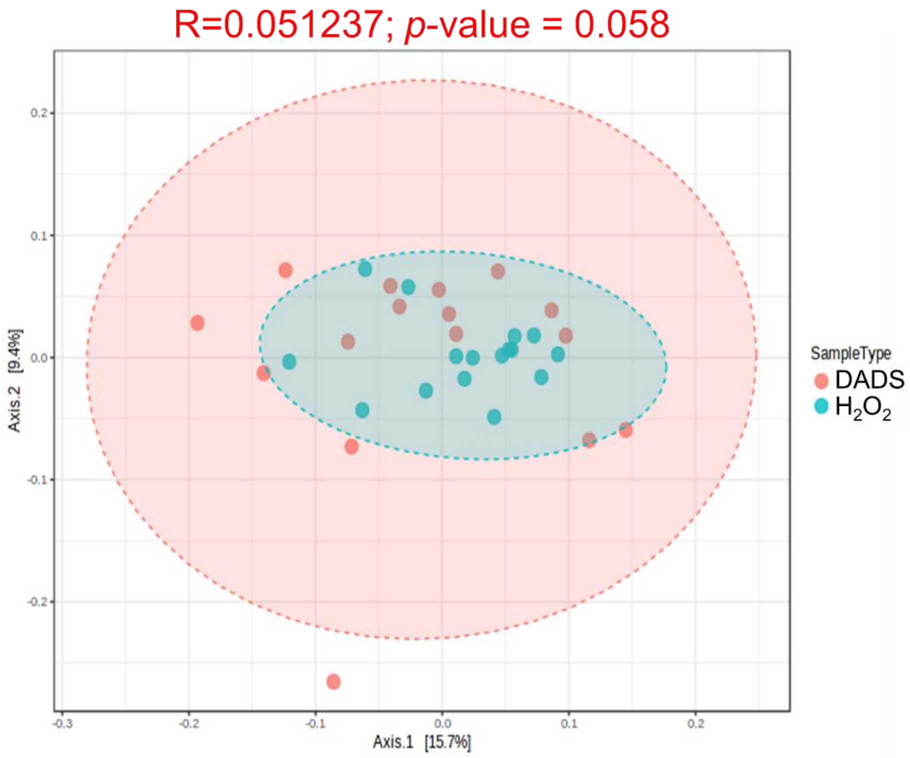

Supplemental Figure 29 ANOSIM analysis of fungal communities treated with DADS and H_2O_2.

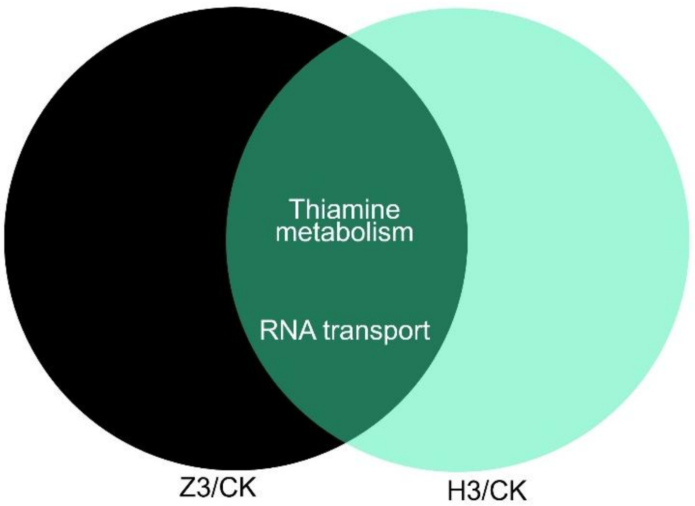

Supplemental Figure 30 Venn diagram of co-variant pathways in Z3 and H3 metagenomes

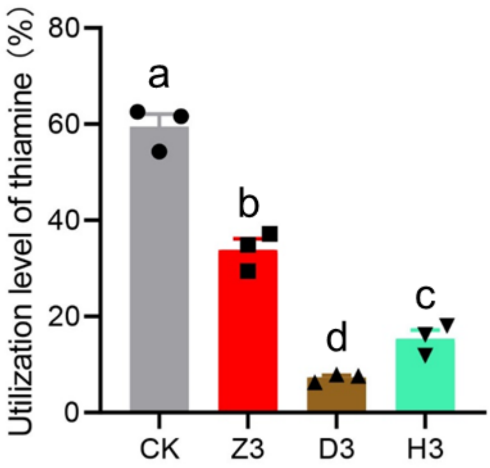

Supplemental Figure 31 Thiamine utilization level of soil microorganism in three treatments. Data are expressed as mean ± standard error. Different lowercase letters indicate significant differences between treatments ($p < 0.05$, according to ANOVA with Duncan's multiple range test). Z3 represents three garlic plants per pot**,** D3 represents DADS treatment at 54.7 μmol kg^{-1} soil, and H3 represents H_2O_2 treatment at 343.4 μmol kg^{-1}.

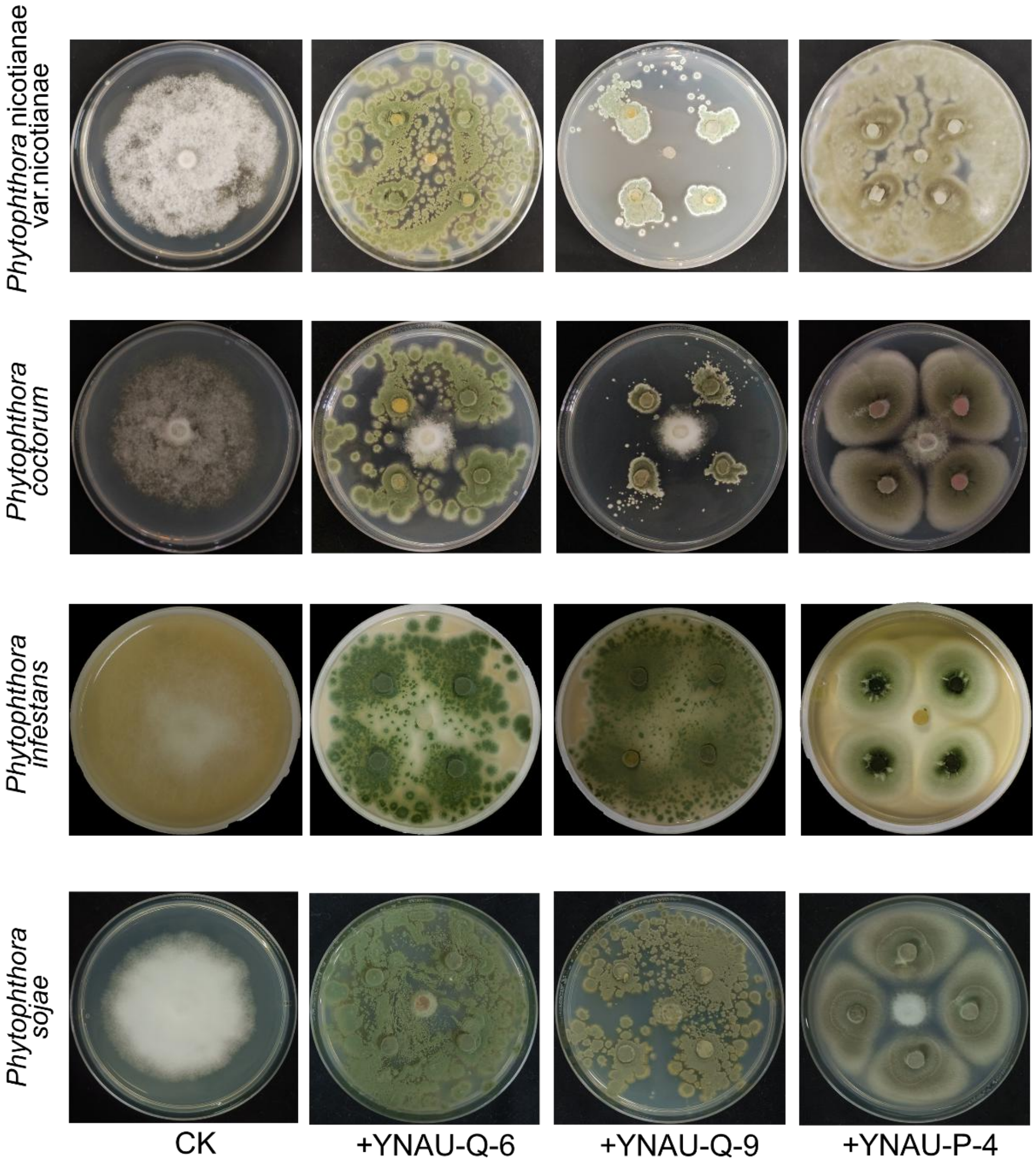

Supplemental Figure 32 Antagonistic effects of three *Penicillium* spp. isolated from the conditioned soil of garlic against different *Phytophthora* spp.

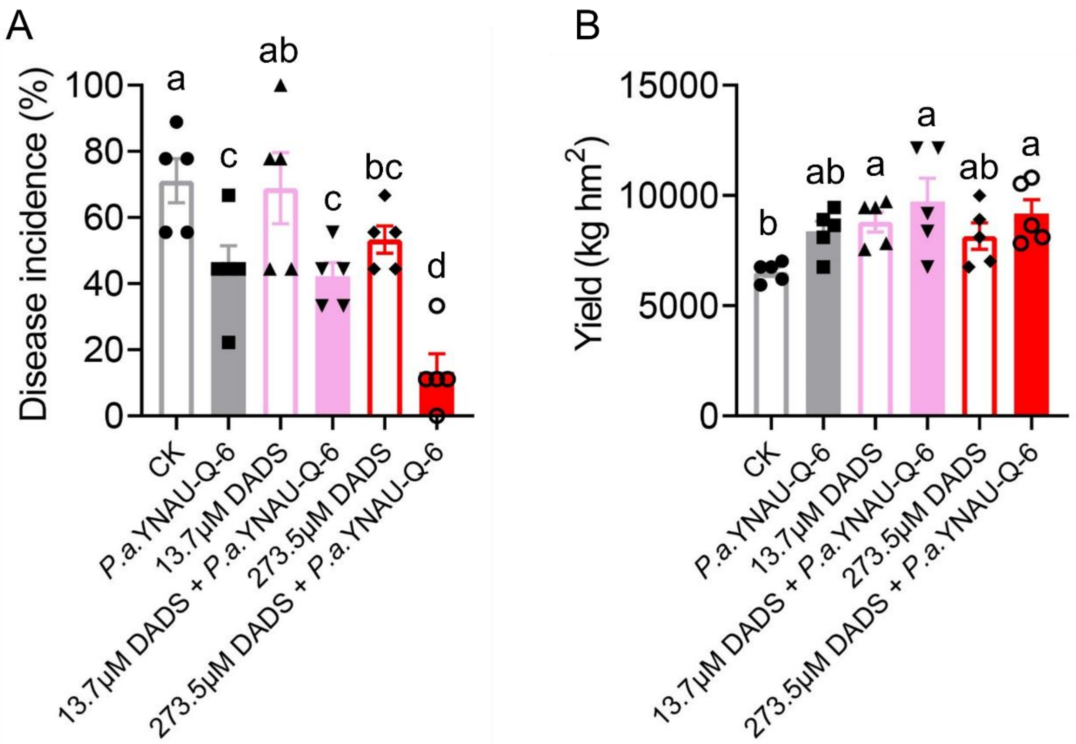

Supplemental Figure 33 Effects of application of *P. allii* YNAU-Q-6 and DADS on the incidence of tobacco black shank and soybean yield.

(A) Incidence of tobacco black shank.

(B) Soybean yield.

Data are expressed as mean ± standard error (n = 5). Different lowercase letters indicate significant differences between treatments ($p < 0.05$, according to ANOVA followed by Duncan's multiple range test).

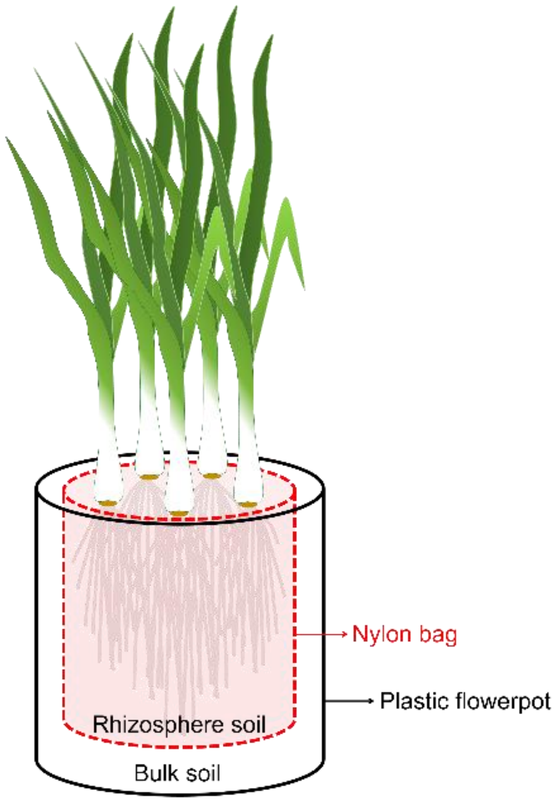

Supplemental Figure 34 Garlic root bag potting experiment device.

Supplemental References

Anisimova, O.K., Shchennikova, A.V., Kochieva, E.Z., and Filyushin, M.A. (2021). Pathogenesis-related genes of PR1, PR2, PR4, and PR5 families are involved in the response to Fusarium infection in garlic (*Allium sativum* L.). Int. J. Mol. Sci. **22**:6688. https://doi.org/10.3390/ijms22136688.

Diao, X.C., Wang, S.B., Diao, W.P., Pan, B.G., Ge, W., and Gao, Q.H. (2019). Cloninig and expression analysis of CaWRKY8 generin pepper under stresses. Acta Bot. Boreal.-Occident. Sin. **39**: 0210-0217. https://doi.org/10.7606/j.esn.1000-4025.

Ding, H.Y., Ali, A., and Cheng, Z.H. (2018). Dynamics of a soil fungal community in a three-year green garlic/cucumber crop rotation system in Northwest China. Sustainability **10**:1391. https://doi.org/10.3390/su10051391.

Guo, P.T., Wu, X.T., Lai, R.Q., Chen, Z.H., Chang, Y., and Bai, J.J. (2019) Allelopathic effects of garlic root exudates on different varieties of flue-cured tobacco. Wuyi Sci. J. **35**:97-102. https://doi: 10.15914 /j.cnki.wykx.2019.02.05.

Luo, L.F., Wang, Z.P., Yan, X.B., Ye, C., Hao, J.J., Liu, X.L., Zhu, S.S., and Yang, M. (2025). Diversified *Alternaria* pathogenicity alters plant-soil feedbacks through leaf-root-microbiome dynamics in agroforestry systems. Hortic. Res. **12**:uhaf137. https://doi.org/10.1093/hr/uhaf137.

Liu, H.J., Wu, J.Q., Su, Y.W., Li. Y,B., Zuo, D.H., Liu, H.B., Liu, Y.X., Mei, X.Y., Huang, H.C., Yang, M., et al. (2021). Allyl isothiocyanate in the volatiles of *Brassica juncea* inhibits the growth of root rot Pathogens of *Panax notoginseng* by inducing the accumulation of ROS. J. Agric. Food Chem. **69**:13713-13723. https://doi.org/10.1021/acs.jafc.1c05225.

Nie, H.L., Huang, S.H., Yang, J.F., Yang Y.M., Zhang J., Yang, H.M., Yang, W.F., Xing, S,L., Jia, L.L., and Yue, Z.L. (2023). Meta analysis of the effects of foliar Se application on grain yield, protein content, and Se accumulation of winter wheat. Chin. J. Eco-Agric. **31**:1997−2010. https://doi.org/10.12357/cjea.20230229.

Tuan, P.A., Park, N.I., Li, X., Xu, H., Kim, H.H., and Park, S.U. (2010). Molecular cloning and characterization of phenylalanine ammonia-lyase and cinnamate 4-hydroxylase in the phenylpropanoid biosynthesis pathway in garlic (*Allium sativum*). J. Agric. Food Chem. **58**:10911–10917. https://doi.org/10.1021/jf1021384.

Visagie, C.M., Houbraken, J., Frisvad, J.C., Hong, S.B., Klaassen, C.H. W., Perrone, G., and Samson, R.A. (2014). Identification and nomenclature of the genus *Penicillium*. Stud. Mycol. **78**:343-371. https://dx.doi.org/10.1016/j.simyco.2014.09.001.

Wu, J.Q., Liu, J.Y., Sun, J.W., Liu, Y.P., He, T., Zhao, J., Mei, X.Y., Liu, Y.X., Yang, M., and Zhu, S.S. (2024). Diallyl trisulfide acts as a soil disinfestation against the *Ilyonectria destructans* through inducing the burst of reactive oxygen species. J. Agric. Food Chem. **72**:9669-9679. https://doi.org/10.1021/acs.jafc.4c01422.

Yu, B.J., Li, J.L, G. Moussa, M.G., Wang, W.C., Song, S.S., Xu, Z.C., Shao, H.F., Huang, W.X., Yang, Y.X., Han, D., et al. (2024). Molybdenum inhibited the growth of *Phytophthora nicotiana* and improved the resistance of *Nicotiana tabacum* L. against tobacco black shank. Pestic. Biochem. Phys. **199**:105803-105815. https://doi.org/10.1016/j.pestbp.2024.105803.

Zhao, Z.X., Yan, W.R., Wang, Bao., Chen, Yuan, Wang, H.F., and Xiao, T.B. (2022). Increase of defense enzyme activity and expression of resistance-related genes in pepper induced by *Bacillus* sp. Ya-1. Mol. Plant Breed. **20**:2699-2706. https://doi.org/10.13271/j.mpb.020.002699.

Zhang, J., Lv, J, Xie J. M., Gan, Y.T., Coulter, J.A., Yu, J.H., Li, J., Wang, J.W., and Zhang, X.D. (2020). Nitrogen source affects the composition of metabolites in pepper (*Capsicum annuum* L.) and regulates the synthesis of capsaicinoids through the GOGAT-GS pathway. Prog. Artif. Intell. **9**:150. https://doi.org/10.3390/foods9020150.

Zhang, Y.J., Ye, C., Su, Y.W., Peng, W.C., Lu, R., Liu, Y.X., Huang, H.C., He, X.H., Yang, M., and Zhu, S.S. (2022). Soil acidification caused by excessive application of nitrogen fertilizer aggravates soil-borne diseases: Evidence from literature review and field trials. Agr. Ecosyst. Environ. **340**:108176. https://doi.org/10.1016/j.agee.2022.108176.

Zheng, J., Yan, F., Pan, Z. P., Feng, Z.J., and Li, X.Y. (2019). Meta analysis on the effect of biogas slurry on crop yield in China. China Biogas， **37**: 78-84.
